# Two Color Imaging of Different Hypoxia Levels in Cancer Cells

**DOI:** 10.1021/jacs.2c12493

**Published:** 2023-01-19

**Authors:** Antoine L. D. Wallabregue, Hannah Bolland, Stephen Faulkner, Ester M. Hammond, Stuart J. Conway

**Affiliations:** † Department of Chemistry, Chemistry Research Laboratory, University of Oxford, Mansfield Road, Oxford OX1 3TA, U.K.; ‡ Oxford Institute for Radiation Oncology, Department of Oncology, University of Oxford, Old Road Campus Research Building, Oxford OX3 7DQ, U.K.

## Abstract

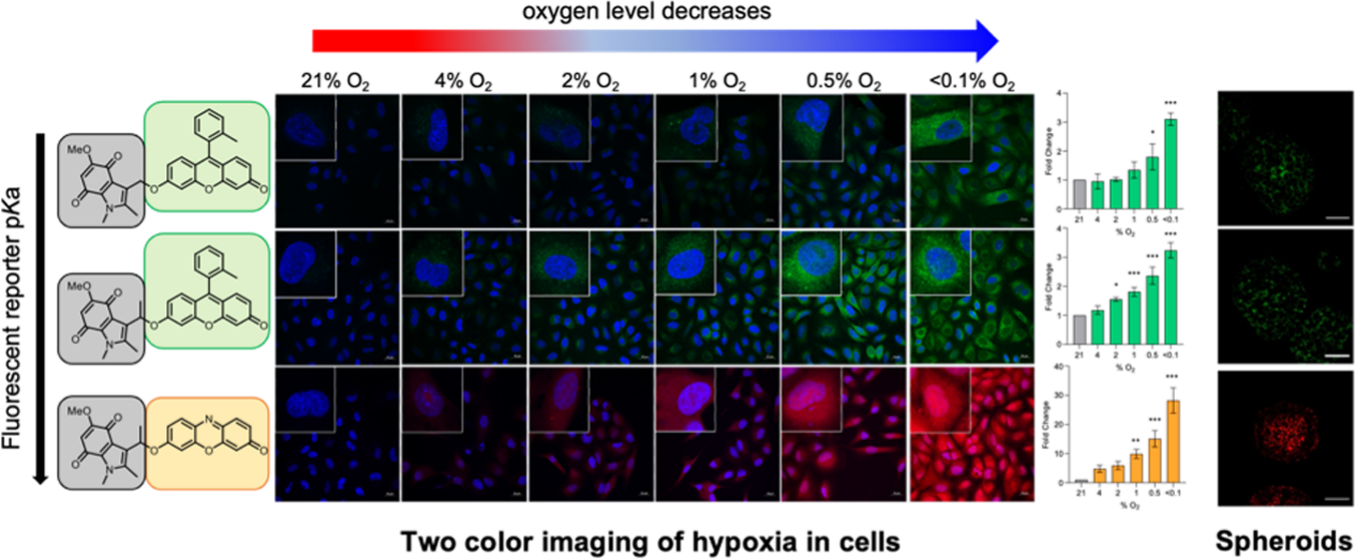

Hypoxia (low oxygen levels) occurs in a range of biological contexts, including plants, bacterial biofilms, and solid tumors; it elicits responses from these biological systems that impact their survival. For example, conditions of low oxygen make treating tumors more difficult and have a negative impact on patient prognosis. Therefore, chemical probes that enable the study of biological hypoxia are valuable tools to increase the understanding of disease-related conditions that involve low oxygen levels, ultimately leading to improved diagnosis and treatment. While small-molecule hypoxia-sensing probes exist, the majority of these image only very severe hypoxia (<1% O_2_) and therefore do not give a full picture of heterogeneous biological hypoxia. Commonly used antibody-based imaging tools for hypoxia are less convenient than small molecules, as secondary detection steps involving immunostaining are required. Here, we report the synthesis, electrochemical properties, photophysical analysis, and biological validation of a range of indolequinone-based bioreductive fluorescent probes. We show that these compounds image different levels of hypoxia in 2D and 3D cell cultures. The resorufin-based probe **2** was activated in conditions of 4% O_2_ and lower, while the Me-Tokyo Green-based probe **4** was only activated in severe hypoxia—0.5% O_2_ and less. Simultaneous application of these compounds in spheroids revealed that compound **2** images similar levels of hypoxia to pimonidazole, while compound **4** images more extreme hypoxia in a manner analogous to EF5. Compounds **2** and **4** are therefore useful tools to study hypoxia in a cellular setting and represent convenient alternatives to antibody-based imaging approaches.

## Introduction

Hypoxia, defined as insufficient oxygen, plays a key role in the pathogenesis of many diseases, including myocardial ischemia, cardiovascular disease, chronic kidney disease, arthritis, and cancer.^1^ Hypoxia is a common feature of solid tumors and is associated with resistance to standard therapies, increased metastasis, and poor patient prognosis.^2^ Regions of chronic hypoxia occur beyond the diffusion limit of oxygen in metabolically active cancer cells, typically 100 μm, while acute hypoxia can occur within a tumor resulting from changes in red blood cell flux and vascular remodeling. Notably, cycling or intermittent changes in oxygen levels also occurs in tumors and can be exacerbated during therapy.^3^ Typically, physiological levels of oxygen (physoxia) are defined as 3–7% O_2_, tumor hypoxia is defined as <2% O_2_, and extreme levels of hypoxia (<0.1% O_2_) termed radiobiological hypoxia, are those most associated with resistance to radiotherapy.^4,5^ Importantly, the biological response differs in an oxygen-dependent manner, for example, while the hypoxia-inducible factors (HIFs) are induced at both 2 and <0.1% O_2_, the DNA damage response or unfolded protein response is restricted to the lower oxygen tension.^6^ There is an unmet need for imaging tools that not only detect hypoxia but also inform on the level of oxygen present because median oxygen concentrations within a tumor can range from 4.2–0.1% O_2_.^4^

**Figure 1:**
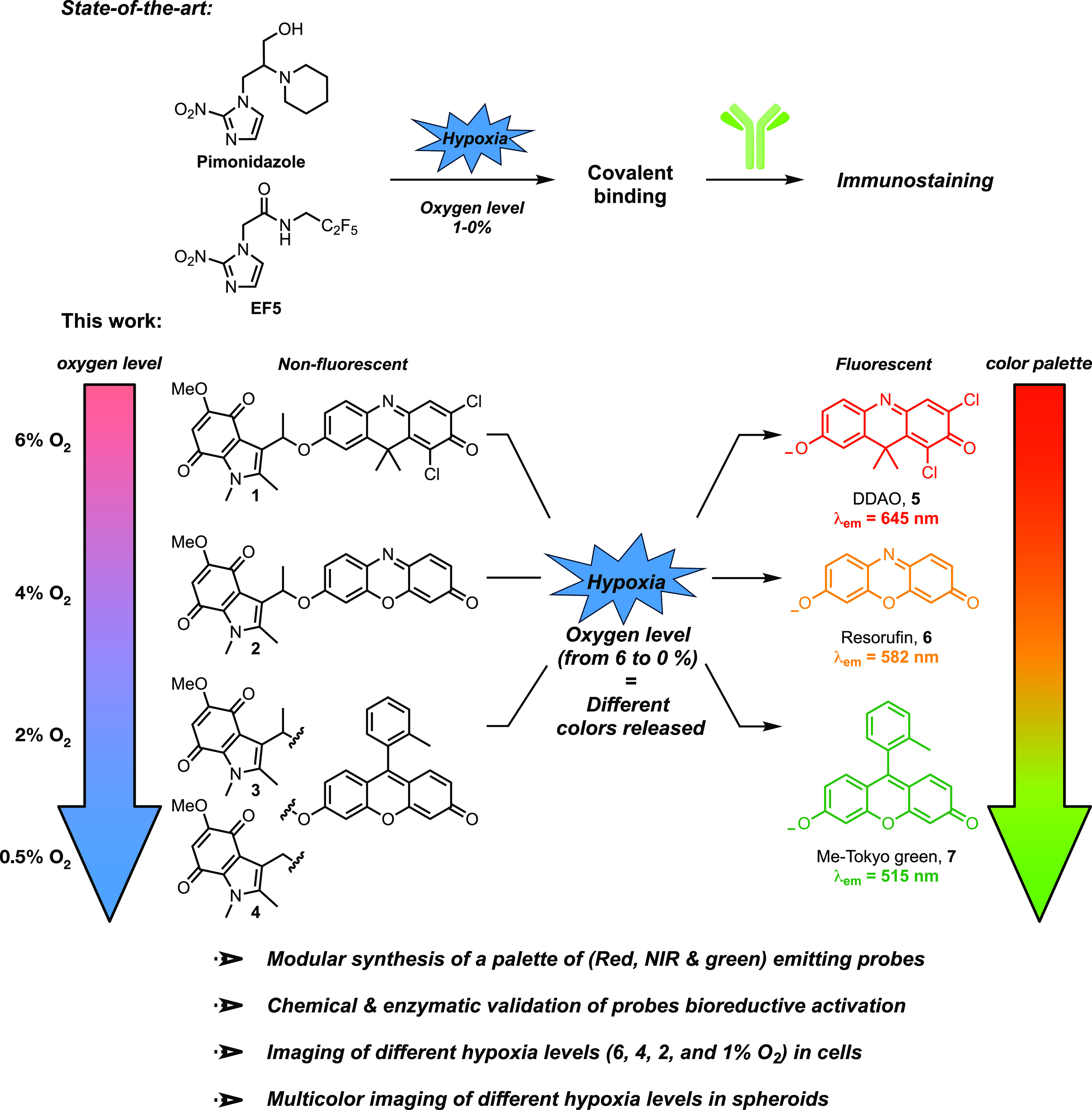
Design of hypoxia-sensing fluorescent probes based on the indolequinone bioreductive group.

One biological response to hypoxia is the production of reductase enzymes (cytochrome P450 reductase and nitroreductase) that catalyze oxygen-sensitive bioreductive reactions. These enzymes have been exploited to develop hypoxia-sensing prodrugs^7−14^ and imaging agents, including pimonidazole and EF5 that are routinely used in preclinical studies as indicators of hypoxia (Figure 1).^15−17^ Both pimonidazole and EF5 contain nitroimidazole groups that are reduced by reductases in hypoxia to generate products that are covalently trapped and can be detected using immunostaining. In contrast, hypoxia-sensing fluorescent probes^18^ are powerful tools that allow the detection and imaging of hypoxia with high sensitivity and spatiotemporal resolution with no need for subsequent detection steps involving immunostaining. In general, such probes function by having their emission properties suppressed by addition of a bioreductive group, examples of which include nitroarenes,^8,18^ quinones,^19^ azo,^20−23^ or azide^24^ groups, that undergo reductase-catalyzed reactions to reveal the fluorophore in an oxygen-dependent manner. Of the hypoxia-sensing probes developed to date, only the azo-based probes reported by Piao et al. and Zhang et al. can detect milder levels of hypoxia.^21,25^ These probes are both based on the arylazo motif, which releases positively charged amine motifs that localize in the mitochondria or lysosomes.

Here, we report a complementary approach, based on the^4,5^ indolequinone scaffold, to develop hypoxia-sensing probes that detect different levels of hypoxia in 2D and 3D cell cultures. We show that the indolequinone groups are readily amenable to chemical modification through ether bond formation with the commonly used fluorophores 9*H*-(1,3-dichloro-9,9-dimethylacridin-2-one-7-yl) (DDAO),^26−28^ resorufin,^29^ and Me-Tokyo green,^30^ affording a range of hypoxia-sensing fluorescent probes **1–4** (Figure 1). We demonstrate that these probes are enzymatically reduced in an oxygen-dependent manner to reveal a palette of fluorophores with emission maxima ranging through red, orange, and green. Varying the p*K*_a_ of the dye and the substitution on the methylene unit of the indolequinone results in compounds that are activated at different oxygen concentrations, ranging from ≤6, ≤4, ≤2, and ≤1% and at different rates. Validation of these probes in 2D and 3D cell cultures demonstrates that they are powerful and convenient tools for sensing a range of different hypoxia levels.

## Results and Discussion

**Figure 2:**
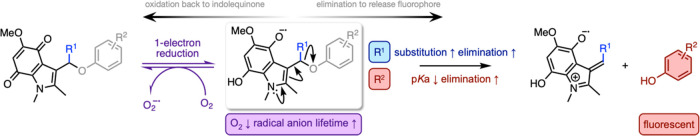
Amount of fluorescent probe released from the pro-fluorophore depends on the lifetime of the radical anion and the rate of elimination of the fluorophore from this species. The lifetime of the radical anion is increased as O_2_ decreases (i.e., in hypoxia), and the rate of fluorophore elimination is increased by addition of a substituent *R*^1^ and a decrease in the fluorophore p*K*_a_ (*R*^2^).

The indolequinone bioreductive group has previously been used as the basis for hypoxia-activated prodrugs that release phosphoramidate-based DNA crosslinkers,^31^ 5-fluorodeoxyuridine,^32^ or the topoisomerase I inhibitor SN38.^33^ It has also been used in the development of a ^19^F NMR probe to detect hypoxia^34^ and fluorescence-based hypoxia imaging agents.^19,35−37^ Studies by Everett et al.^19^ and Swann et al.^38^ showed that this system works as an excellent mechanism for sensing hypoxia, as not only the indolequinone undergoes rapid reduction to form a radical anion but the back oxidation is also fast. Therefore, the radical anion is poised to either oxidize back to the quinone or be eliminated to release the fluorescent product.^19^ The amount of fluorescent product formed is related to both the lifetime of the radical anion (and therefore O_2_ concentration) and the rate of the elimination step. The rate of the elimination step can be accelerated by introduction of a substituent at the benzylic position of the indolequinone (*R*^1^Figure 2), which stabilizes the developing carbocation, and by lowering the p*K*_a_ of the fluorophore to make it a better leaving group. To design fluorescent probes that sense moderate hypoxia, we reasoned that we must employ fluorophores with a lower p*K*_a_ to enable rapid elimination from a short-lived radical anion. To develop probes for extreme hypoxia, we used a fluorophore with a higher p*K*_a_, slowing the rate of elimination, meaning that it is only released from a longer-lived radical anion that only occurs in very low oxygen conditions. We hypothesized that combining the indolequinone derivatives with fluorophores that have a range of p*K*_a_ values would provide a range of probes that release fluorophores with different emission wavelengths at different levels of oxygen. Pro-fluorophores **1**–**4** are based on the DDAO, resorufin, and Me-Tokyo green fluorophores, which have p*K*_a_ values of 5, 5.8, and 6.7, respectively (Figure 1). We reasoned that the wide range of fluorophore p*K*_a_ values and addition of a methyl group on the benzylic unit of the indolequinone would result in fluorophore release across a wider range of oxygen concentrations than observed by Everett et al. (Figure 2).

**Scheme 1:**
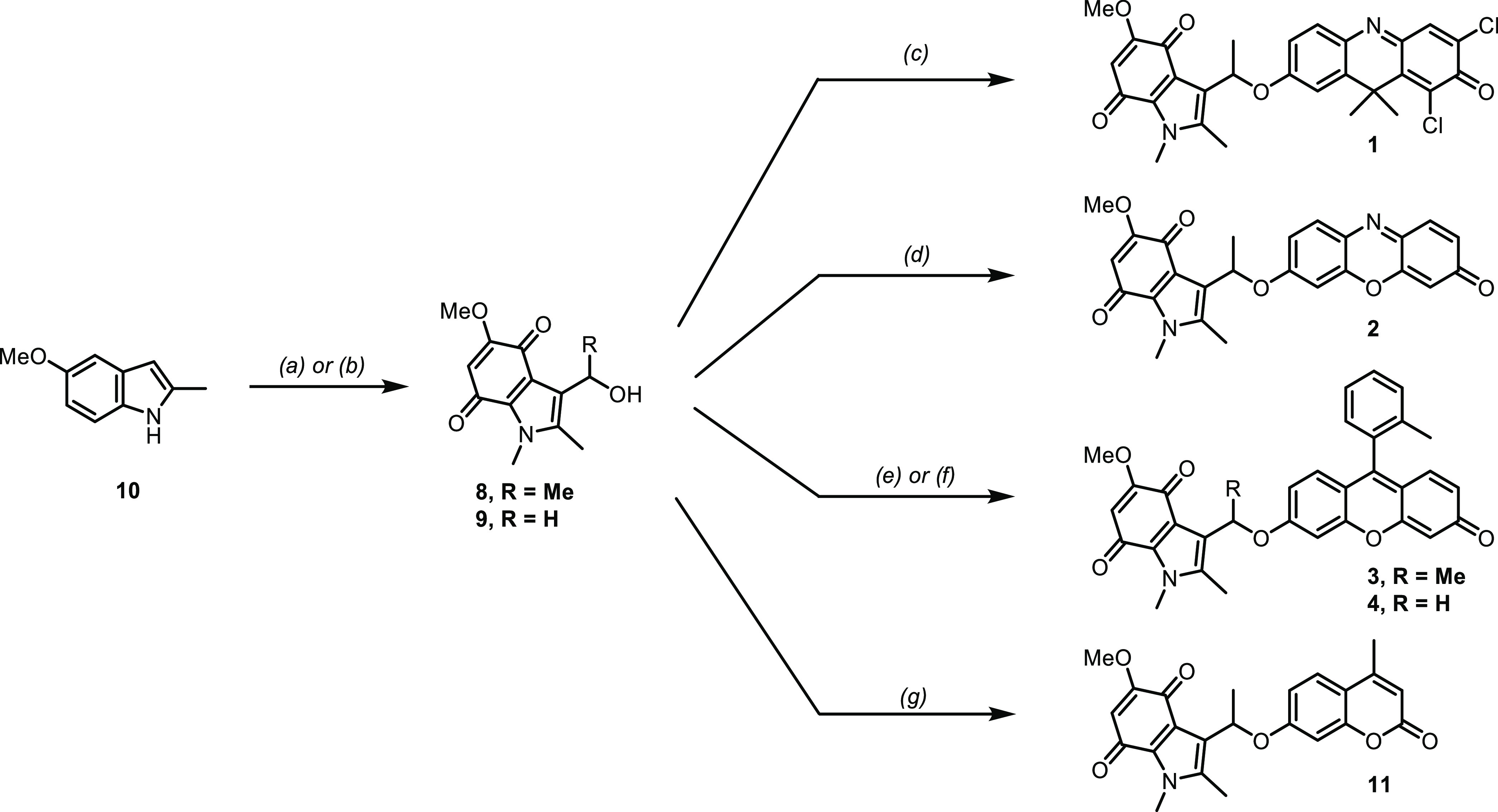
Synthesis of the Indolequinone Derivatives **1–4, 8, 9**, and **11** For **8** (a) (i) **10** (1.0 equiv), EtMgBr (1.9 equiv), Et_2_O, reflux, 30 min, then AcCl (6.0 equiv), 1 h; (ii) NaH (2.1 equiv), THF, 50 °C, 30 min, then MeI (8.5 equiv), THF, reflux, 1 h; (iii) HNO_3_, AcOH, −10 °C, 1 h, then rt, 1 h; (iv) Sn, HCl, EtOH, 80 °C, 1 h; (v) (K_2_[NO(SO_3_)_2_]) (5.0 equiv), phosphate buffer pH 6 rt, 1 h; (vi) NaBH_4_, MeOH, 0 °C, 1 h, 37% over 6 steps; for **8** (b) (i) DMF, POCl_3_, 0 °C, 10 min then **10** (1.0 equiv) −10 °C, 30 min; (ii) NaH (1.5 equiv), DMF, rt, 2 h, then MeI (1.2 equiv), rt, 2 h; (iii) HNO_3_ (22.5 equiv), AcOH, 0 °C, 45 min, then rt, 3 h; (iv) Sn (5.2 equiv), HCl, EtOH, 80 °C, 1 h; (v) (K_2_[NO(SO_3_)_2_]) (5.0 equiv), phosphate buffer pH 6, rt, 1 h; (vi) NaBH_4_ (5.0 equiv), MeOH/THF, 0 °C, 1 h, 26% over 6 steps for **9**; (c) **8** (1.0 equiv) DDAO (2.5 equiv), DIAD (3.0 equiv) PPh_3_ (3.0 equiv), THF, rt, 42 h, 15%; (d) **8** (1.0 equiv) resorufin (2.5 equiv), DIAD (3.0 equiv) PPh_3_ (2.5 equiv), THF, 50 °C, 18 h, 42%; (e) **8**, (1.0 equiv) Me-Tokyo green (2.0 equiv), DIAD (3.0 equiv) PPh_3_ (3.0 equiv), THF, 50 °C, 24 h, 50%; (f) (i) **9** (1.0 equiv), SOCl_2_ (34 equiv), CH_2_Cl_2_, rt, 1 h, 62%; (ii) Me-Tokyo green (1.0 equiv), Cs_2_CO_3_ (2.0 equiv) TBAI (1.0 equiv), DMF, rt, 20 h, 68%; (g) **8**, (1.0 equiv) 7-hydroxy-4-methylcoumarin **12** (2.5 equiv), DIAD (3 equiv) PPh_3_ (2.5 equiv), THF, rt, Ar, 2.5 h, 33%.

The indolequinone precursors **8** and **9** were synthesized in 37 and 26% overall yields, respectively, from the commercially available 5-methoxy-2-methylindole **7** (Scheme 1) using modified literature protocols.^19,38^ The DDAO (**5**)^26^ and Me-Tokyo green (**7**)^30^ fluorophores were then prepared in yields of 86 and 88%, respectively (see the Supporting Information). With the required building blocks in hand, we selected the Mitsunobu reaction to alkylate the phenol groups of the reporters and prepare compounds **1**, **2**, and **3**. The indolequinone **8** was conjugated to DDAO (**5**), resorufin, and Me-Tokyo green (**7**) using the conditions shown in Scheme 1. A clear relationship between the yield of the reactions and the p*K*_a_ of the phenol groups was observed. DDAO (p*K*_a_ 5)^27^ and resorufin (5.8) reacted to afford compounds **1** and **2** in 15 and 42% yields, respectively, while Me-Tokyo green (p*K*_a_ 6.7)^39^ reacted to give compound **3** in 50% yield. This trend observed in the yields is attributed to the low nucleophilicity of the DDAO and resorufin phenolates, and similar low reactivity has been previously observed for resorufin.^9^ Compound **4** was prepared *via* the chloride **S8**, which was generated in 62% yield by treatment of **9** with thionyl chloride. This chloride was then reacted with Me-Tokyo green in the presence of cesium carbonate to afford the desired product **4** in 68% yield. Finally, to have a relevant reference for the assessment of compounds **1–4** photophysical and electrochemical properties, the previously reported indolequinone-based coumarin **11** was prepared in 33% yield using 7-hydroxy-4-methylcoumarin **12** and the Mitsunobu reaction conditions depicted in Scheme 1. Full details of the fluorescent probe synthesis can be found in the Supporting Information.

With compounds **1–4** in hand, we compared their photophysical properties to the parent fluorophores (Figure S1). The attachment of indolequinone **8** or **9** to the dye led to an almost complete reduction in fluorescence intensity for all three dyes. Probe **1** displays a more than 8-fold decrease of its emission intensity compared to DDAO, while probes **2**, **3**, **4**, and **11** exhibit no detectable emission signal, as expected.^19,36,40^

To investigate the electrochemical properties of the probes and compare these to their parent dyes, we next conducted cyclic voltammetry (CV) experiments (Supporting Information).^41^ Compounds **11**, **3**, and **4** have similar voltammograms, which show a single irreversible peak at a reduction potential of −1.21 V (*vs* Ag/AgNO_3_) (Figure S2). This is consistent with the reduction of the indolequinone component of these molecules, followed by fragmentation (i.e., an irreversible chemical reaction) and indicates that the bioreduction of these compounds should occur under similar conditions (Figure S2).^19^ Compounds **1** and **2** have more complex voltammograms that contain multiple reduction peaks. As these peaks are not present in the parent indolequinone or dye voltammograms, this indicates that coupling of the compounds together affects their electrochemical and redox properties, in part by removing the ability of the phenolate to delocalize into the conjugated π-system of the dyes.^42^ Compound **1** displays two reversible reduction peaks at −0.8 and −1.27 V (*vs* Ag/AgNO_3_), while **2** displays two pseudo-reversible reductions at −1.06 and −1.27 V (*vs* Ag/AgNO_3_). The least negative reduction peaks of **1** and **2** likely correspond to reduction of the indolequinone and indicate that both of these compounds should undergo more facile bioreduction than **11**, **3**, or **4**. The presence of additional reductions in the voltammograms for compounds **1** and **2** indicates that their redox chemistry is more complicated and that this could impact on their behavior in a biological setting. Given the similar redox properties of probes **3**, **4**, and **11**, we decided to focus on **3** and **4**, as the fluorescence properties of Me-Tokyo Green fluorophore they release are more suitable for use in cells and spheroids than the 7-hydroxy-4-methylcoumarin released by **11**.

**Figure 3:**
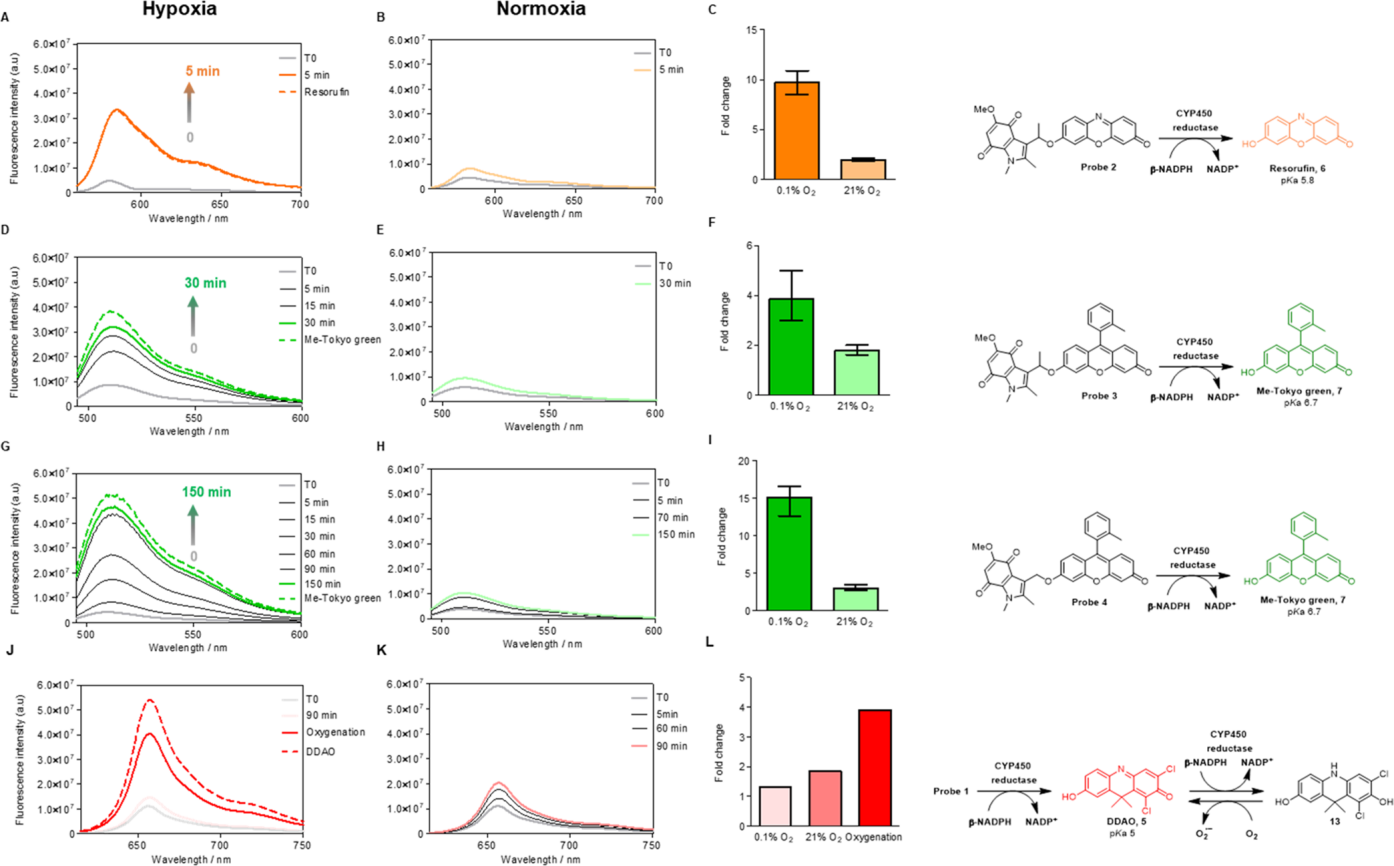
Indolequinone-based probes **2**–**4** undergo oxygen-dependent cytochrome P450 reductase (PH51)-catalyzed reduction to give the corresponding fluorophore. Compound **2** (1 μM) was treated with 0.72 ng/μL cytochrome P450 reductase (PH51) and β-NADPH (20 mM) in hypoxic (0.1% O_2_, A) or normoxia (21% O_2_, B) over 5 min, as described in the Supporting Information. Plain line fluorescence intensity data were collected after the time indicated with an excitation wavelength of 545 nm. Slits 3, 3 nm (representative graphs are shown, *n* = 3). The dashed line shows the fluorescence intensity of resorufin (1 μM) under the same conditions. (C) Quantification of the fluorescence increase shown by compound **2**. Error bars represent SD. *n* = 3. (D) Compound **3** (1 μM) was treated with 0.72 ng/μL cytochrome P450 reductase (PH51) and β-NADPH (20 mM) in hypoxic (0.1% O_2_), (D) or normoxia (21% O_2_), (E) over 30 min, as described in the Supporting Information. Fluorescence intensity data were collected after the time indicated in the figure with an excitation wavelength of 480 nm. Slits 2, 2 nm (representative graphs are shown, *n* = 3). The dashed line shows the fluorescence intensity of Me-Tokyo green (1 μM) under the same conditions. (F) Quantification of compound **3** fluorescence increase. Error bars represent SD. *n* = 3. (G) Compound **4** (1 μM) was treated with 0.72 ng/μL cytochrome P450 reductase (PH51) and β-NADPH (20 mM) in hypoxic (0.1% O_2_), (G) or normoxia (21% O_2_), (H) over 150 min, as described in the Supporting Information. Fluorescence intensity data were collected after the time indicated in the figure with an excitation wavelength of 480 nm. Slits 2, 2 nm (representative graphs are shown, *n* = 3). The dashed line shows the fluorescence intensity of Me-Tokyo green (1 μM) under the same conditions. (I) Quantification of compound **4** fluorescence increase. Error bars represent SD. *n* = 3. (J) Compound **1** (4 μM) was treated with cytochrome P450 reductase (PH51, *c* = 0.72 ng/μL) and β-NADPH (20 mM) under hypoxia (0.1% O_2_), (J) or normoxia (21% O_2_), (K) over 90 min, as described in Supporting Information. Oxygenation: the product of enzymatic reduction under hypoxia (0.1% O_2_, 90 min) was submitted to normoxic conditions (21% O_2_) for 30 min. Plain line fluorescence intensity data were collected after the time indicated in the figure with excitation at 600 nm. Slits 3, 3 nm. The dashed line shows the fluorescence intensity of DDAO (4 μM). (L) Quantification of the fluorescence increase shown by compound **1**. *n* = 1.

To examine whether probes **1–4** fragment under reductive conditions to release the corresponding fluorescent dyes (**5–7**), both chemical and enzyme-mediated reductions were investigated. Treatment of compounds **1**–**4** with sodium dithionite (Supporting Information)^19,38^ resulted in all probes releasing the corresponding dyes and the hydroquinone derivatives (Figures S3–S6). In contrast, negligible fragmentation of compounds **1–4** was observed in the absence of sodium dithionite, demonstrating that reduction of the indolequinone is required for the release of the fluorescent dye and that the probes are stable to hydrolysis under these conditions. Next, we determined the ability of the fluorogenic probes to undergo bioreduction when treated with NADPH-cytochrome P450 reductase (PH51) (Supporting Information). This class of enzymes is known to metabolize indolequinone derivatives and therefore provides a useful predictive model system for cellular bioreduction of the probes.^19,36^ Compounds **2–4** were treated with NADPH-cytochrome P450 reductase in the presence of β-NADPH, and the fluorescence spectra of the solutions were recorded at the time points shown (Supporting Information and Figure 3). In all cases, a substantial increase in fluorescence was observed, but the time taken for fluorescence recovery (compared to the parent fluorophore) differed between probes. In hypoxia (0.1% O_2_), the resorufin-based probe **2** showed a 10-fold increase in fluorescence and recovery to the same fluorescence level (100%) as the parent resorufin in only 5 min, indicating that the enzymatic bioreduction was complete (Figures 3A–C and S7). The Me-Tokyo Green-based probe **3** showed a 4-fold increase in fluorescence after 30 min, recovering 95% of the Me-Tokyo Green fluorescence (Figures 3D–F and S8). Compound **4** took 150 min to show a 15-fold increase in fluorescence and recovery of 86% of the Me-Tokyo green fluorescence (Figures 3G–I and S9). The higher enzymatic reduction rate of compound **2** compared to compound **3** confirms that the lower p*K*a and less negative reduction potential (*vide supra*) of **2** result in more rapid bioreduction. A comparison of the reaction rates of **3** and **4** demonstrates that the presence of the benzylic methyl group also accelerates the reaction rate, as expected.^19,38^

We were surprised to find that compound **1** only showed a weak increase in fluorescence after 90 min when treated at <0.1% O_2_ with the NADPH-cytochrome P450 reductase and β-NADPH (Figure S10). Given the cyclic voltammetry data for this compound (*vide supra*), we proposed that the cytochrome P450 reductase was not only reducing the indolequinone but also the DDAO, resulting in a nonfluorescent product. HPLC analysis of the enzymatic reduction (Figure S11) confirmed this hypothesis, with the reaction shown to produce both DDAO and its nonfluorescent reduced derivative (**13**). To investigate whether DDAO was susceptible to NADPH-cytochrome P450-mediated reduction, we treated this compound with the enzyme and β-NADPH in hypoxia (<0.1% O_2_) for 65 min and observed the loss of fluorescence, consistent with bioreduction occurring. Interestingly, reoxygenating the reaction media under normoxic conditions (21% O_2_) for 30 min led to 82% fluorescence recovery compared to the parent DDAO (Figure S12). The reduction of the DDAO did not occur under normoxic conditions (Figure S12C) and was not promoted by β-NADPH alone (Figure S12D). These data indicate that compound **1** can undergo bioreduction to produce DDAO, but the dye is also reduced to **13** under the same conditions. However, the reversibility of this reaction allows recovery of fluorescence in normoxia, meaning that this probe might be useful in certain circumstances.

Treatment of probes **1**–**4** with a high concentration of l-glutathione (GSH; 5 mM) had minimal effect on the resorufin-based probe **2** (Figure S15; 18% increase in fluorescence compared to free resorufin after 2 h) and the Me-Tokyo Green-based probe **4** (Figure S17; 6% increase in fluorescence compared to free Me-Tokyo Green after 2 h). The Me-Tokyo Green-based probe **3** (Figure S16) was more substantially affected, showing a 29% increase in fluorescence compared to free Me-Tokyo Green. The DDAO-based probe **1** (Figure S14) showed negligible fluorescence increase (1% compared to free DDAO), which was surprising, given the low p*K*_a_ of the released dye. Based on our results in the enzyme assay and previous work on GSH conjugates,^43−46^ we investigated whether GSH was reducing DDAO or reacting with it. Treatment of DDAO with GSH (5 mM, 2 h) showed almost complete (99.5%) loss of fluorescence. Mass spectrometry analysis showed the presence of some leuco-DDAO, but the DDAO conjugate with GSH was more prevalent, suggesting that DDAO undergoes conjugate addition with GSH, at least at high concentrations. Treatment of resorufin with GSH (5 mM) under the same conditions demonstrated that resorufin retained 82% of fluorescence. Neither the leuco-resorufin nor the GSH conjugate was observed using mass spectrometry, indicating that probe **2** and resorufin are predominantly stable to treatment with GSH.

**Figure 4:**
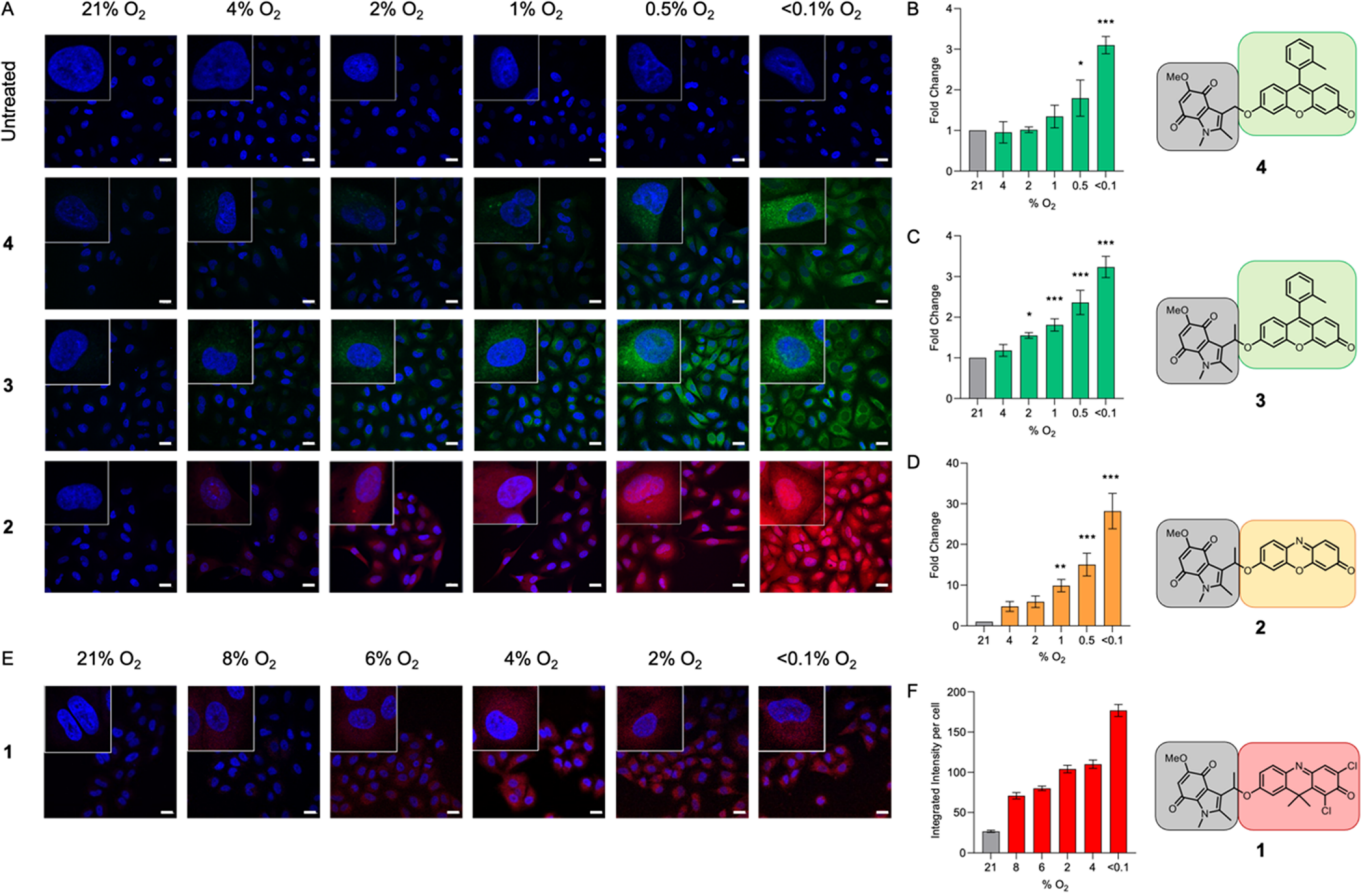
Oxygen-dependent activation of compounds **2**, **3**, or **4** in A549 cells visualized using confocal microscopy. A549 cells were treated with the stated probe (40 μM) and exposed to the indicated concentration of oxygen for 2 h. (A) Representative images of cells incubated with probes **2**, **3**, and **4**. Untreated cells are also shown. Cells were stained with DAPI (blue) to visualize the nucleus. Changes in fluorescence intensity were determined and presented as fluorescence intensity fold change compared to 21% O_2_ for (B) probe **4** (*n* = 3), (C) probe **3** (*n* = 3), and (D) probe **2** (*n* = 3). (E) Representative images of cells incubated with probe **1** with 10 min reoxygenation step. (F) Changes in fluorescence intensity of probe **1** plotted as integrated intensity per cell (*n* = 1). Scale bar = 20 μm. Error bars represent SD.* *p* < 0.05, ** *p* < 0.01, and *** *p* < 0.001.

**Figure 5:**
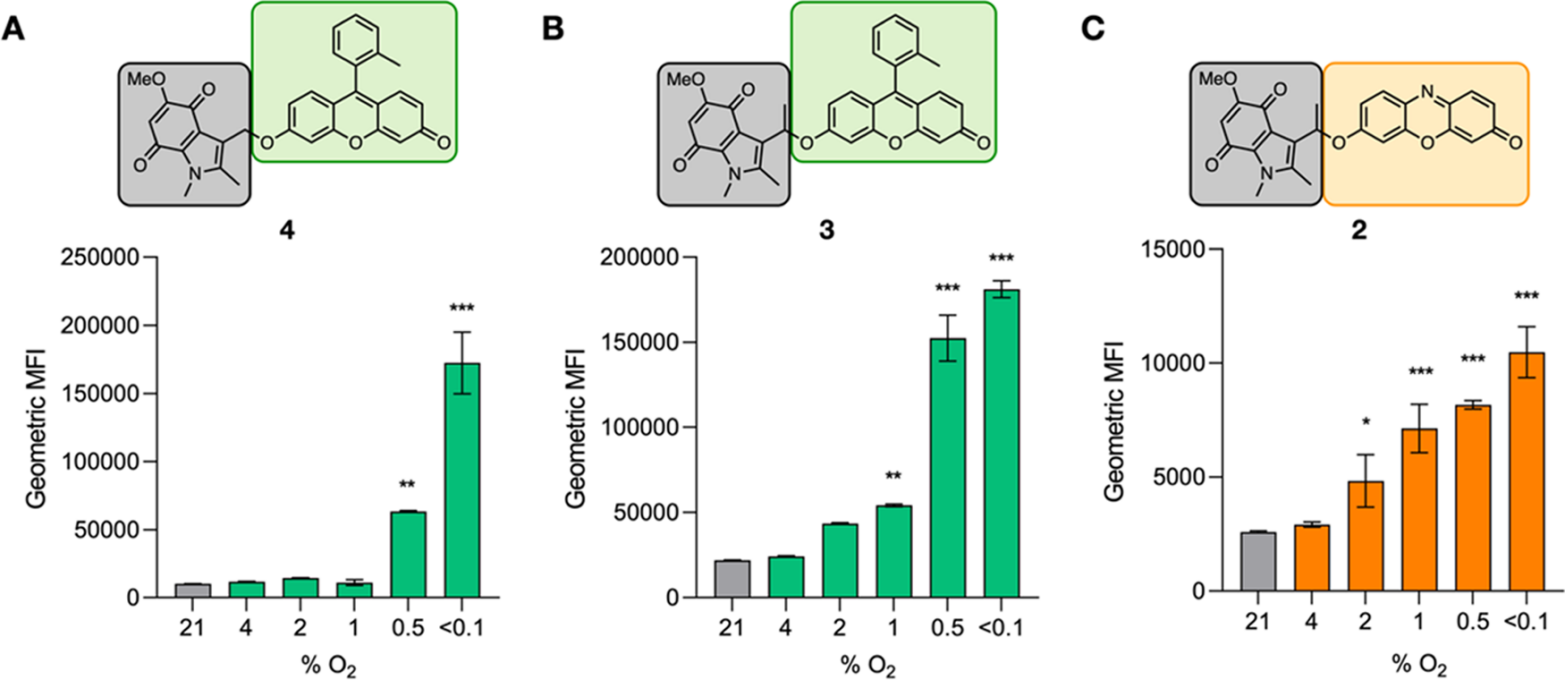
Oxygen-dependent activation of compounds **2**, **3**, or **4** measured using flow cytometry. A549 cells were treated with the indicated probe (40 μM) and exposed to the oxygen concentration shown for 2 h. Cells were processed using flow cytometry, and the geometric mean of the measured fluorescent intensity is shown. (A) probe **4**. (B) probe **2**. (C) probe **3**. Resorufin (PC5.5, ex561/em690/50 nm), Me-Tokyo Green (FITC, ex488/em525/40). Data presented are geometric mean fluorescence intensity. 10,000 cells per condition were analyzed. * *p* < 0.05, ** *p* < 0.01, and *** *p* < 0.001. *n* = 3.

Before evaluating the bioreduction of the probes in cells, we determined that none of the probes **1**–**4** nor their parent fluorophores **5–7** were toxic at concentrations of up to 40 μM in an MTT assay conducted in A549 lung cancer cells over 24 h (Figure S18). We also showed that the parent fluorophores (**5–7**) could be observed using confocal microscopy in A549 cells at concentrations ranging from 10–40 μM (Figure S19). To assess the cellular bioreduction of probes **2**–**4**, A549 cells were treated with the probe (40 μM) at oxygen concentrations of 21% O_2_, 4, 2, 1, 0.5% or <0.1% O_2_ for 2 h. Using confocal microscopy, all compounds were observed to show a hypoxia-dependent increase in fluorescence, with maximum fluorescence observed at <0.1% O_2_ (Figure 4). However, the oxygen level at which fluorescence was initially detected differs between the compounds and correlates with our cyclic voltammetry and enzyme assay data (*vide supra*). Compound **4** displayed increasing fluorescence intensity starting at 1% O_2_, with a ∼1.8-fold increase in intensity when exposed to 0.5% O_2_ (*p* ≤ 0.05), and a maximal 3-fold increase when exposed to <0.1% O_2_ (*p* < 0.001) compared to normoxic controls. No significant increase in fluorescent intensity was observed at the higher oxygen concentrations (2, 4% O_2_) tested (Figure 4A,B). Compound **3** displayed a significant increase in fluorescence intensity starting at 2% O_2_ (*p* < 0.5) and at 1% (*p* < 0.001), 0.5% (*p* < 0.001), and <0.1% O_2_ (*p* < 0.001). Maximum intensity was observed at <0.1% O_2_, with a 3.5-fold increase in fluorescence compared to the normoxic control observed (Figure 4C). A 5-fold increase in fluorescence intensity was observed when compound **2** was exposed to 4% O_2_; the fluorescence increase was similar at 2% O_2_ but increased further, with a 10-fold increase at 1% O_2_ (*p* < 0.01), a 15-fold increase at 0.5% O_2_ (*p* < 0.001), and a 30-fold increase when exposed to <0.1% O_2_ (*p* < 0.001) compared to normoxic controls (Figure 4D). To corroborate the confocal microscopy data, we used flow cytometry as an alternative method to analyze the increase in fluorescence produced by the probes in the same range of oxygen concentrations (21% O_2_, 4, 2, 1, 0.5% or <0.1% O_2_; Figure 5). The flow cytometry data show the same trends as the confocal microscopy results, with compound **2** releasing resorufin at higher oxygen concentrations than compound **4** releases Me-Tokyo green, while compound **3** has intermediate oxygen sensitivity.

Given the unexpected behavior of compound **1** in the enzyme assay and when treated with GSH, we wanted to investigate whether compounds **1** and the DDAO fluorophore were bioreduced in a cellular setting. A549 cells were incubated with compound **1** (40 μM) and exposed to <0.1% O_2_ for 2 h. Then, cells were either fixed inside the hypoxia chamber or exposed to 5, 10, 20, 30, or 60 min reoxygenation to 21% O_2_. Minimal fluorescent intensity was observed when cells were fixed inside the hypoxia chamber or after 5 min reoxygenation. In contrast, maximal fluorescence intensity was reached following reoxygenation for just 10 min, with a reduction in fluorescence intensity observed after this time. This observation indicates that DDAO is reduced in cells at <0.1% O_2_ but, as in the enzyme assay, is rapidly reoxidized upon exposure to 21% O_2_ (Figure S21).

Consequently, a 10 min reoxygenation step was incorporated into experiments to assess the hypoxia-dependent reduction of compound **1** in cells. A549 cells were treated with compound **1** (40 μM) exposed to 8, 6, 4, 2%, or <0.1% O_2_ for 2 h, followed by fixing inside the hypoxia chamber or exposure to 10 min reoxygenation at 21% O_2_ followed by fixation. Like the other probes, compound **1** showed maximum fluorescence intensity at <0.1% O_2_, with a 6.7-fold increase in fluorescence observed. A 2.5-fold fluorescence increase was observed at 8% O_2_, indicating that the lower p*K*a value of DDAO enables this probe to image very mild levels of hypoxia (Figure 4E,F). However, the need for a reoxygenation step means that, while this compound can be used in a cellular setting, it is not suitable for application to more complex systems such as spheroids.

**Figure 6:**
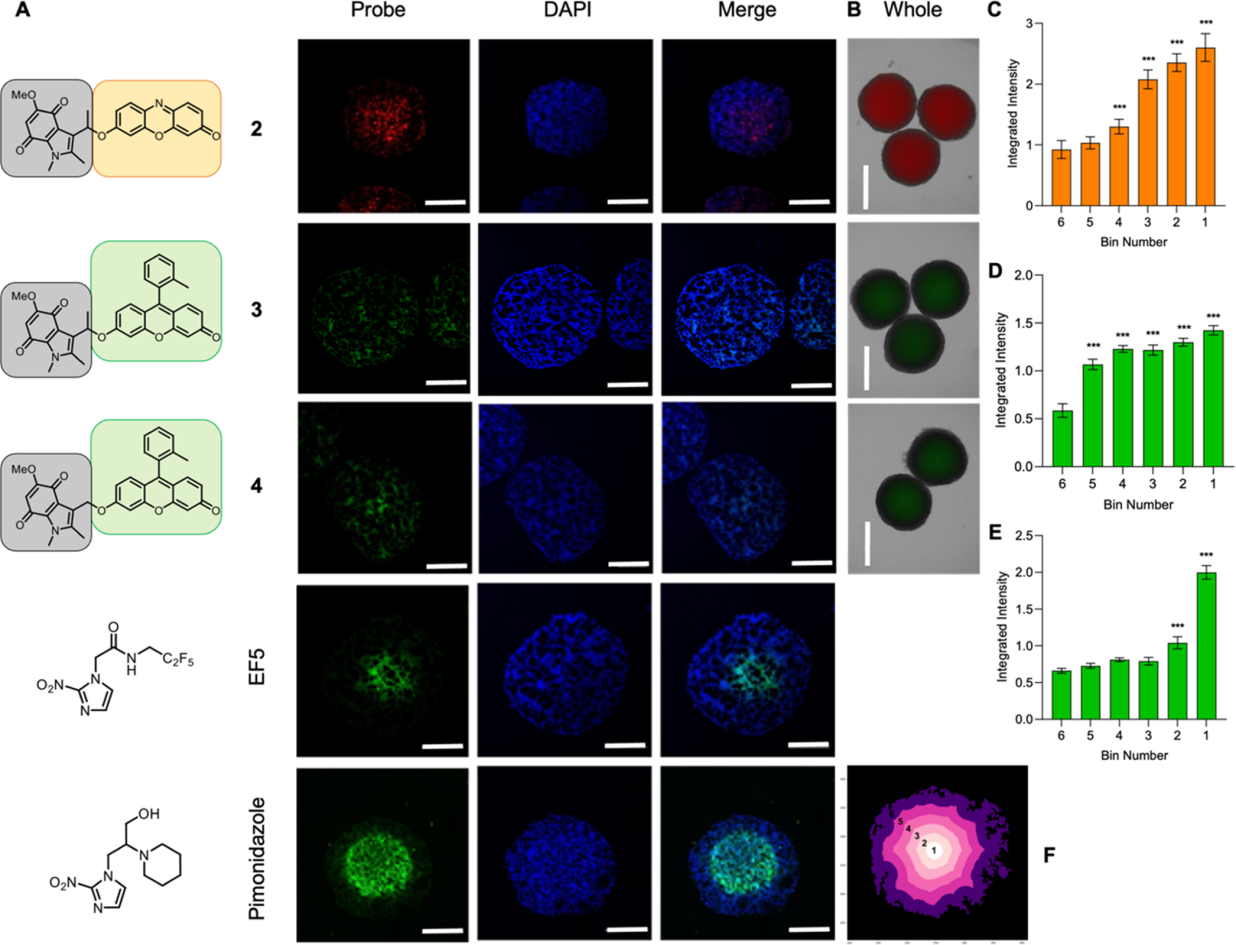
Use of probes **2**, **3**, and **4** in 3D cell culture spheroid models. HCT116 spheroids were incubated with the indicated probe (40 μM), pimonidazole (100 μM), or EF5 (200 μM) for 1.5 h. Scale bar = 200 μm. (A) Representative images of cryo-sectioned spheroids incubated with the indicated probes showing an increase in fluorescence intensity toward the hypoxic center of the spheroids. (B) Live cell imaging of intact whole spheroids treated with the indicated probe. Scale bar = 600 μm. (C) Quantification of the fluorescence intensity of compound **2** in panel A (*n* = 7). (D) Quantification of the fluorescence intensity of compound **3** in panel A (*n* = 7). (E) Quantification of the fluorescence intensity of compound **4** in panel A (*n* = 7). (**F**) Representative image of the quantification method used to quantify fluorescence intensity of probe distribution within the spheroid. Significance compared against region 6. Error bars represent SD. ***p < 0.001.

Having established the suitability of compounds **2**, **3**, and **4** to measure different levels of oxygen in a 2D cellular setting, we investigated the ability of these probes to measure oxygen levels in the more complex and physiologically relevant spheroid 3D cell model. HCT116 colorectal cancer cells were selected based on their propensity to form spheroids.^24,47^ The presence of hypoxic cores in HCT116 spheroids was confirmed using positive pimonidazole and EF5 staining (Figure 6A). We first treated the spheroids with the parent dyes, resorufin or Me-Tokyo Green, to establish that the release dyes could diffuse evenly throughout the spheroids (Figure S20). This confirmed that there is no impediment to dye diffusion in the spheroids. We then investigated the bioreduction of compounds in this setting; spheroids were incubated with compounds **2**, **3**, or **4** (40 μM) for 1.5 h. This length of incubation was chosen as it is comparable to the time required to visualize a pimonidazole signal (Figure 6A). To calculate the mean fluorescence intensity at different depths within the spheroid, CellProfiler was used to identify individual cells and determine their distance from the center of the spheroid.^48^ Region 1 represents the central core of the spheroid, and region 6 represents the periphery of the spheroid (Figure 6E). As compound **2** showed a turn-on response at <4% O_2_, spheroids were grown to an average size of 300 μm in diameter. Compound **2** displayed significant fluorescence intensity starting from the peripheral region 4 and increasing toward the center of the spheroid—region 1. Region 1 exhibited a 3.7-fold increase in fluorescence intensity compared to peripheral region 6 (Figure 6B). As compound **3** had a turn-on response at <2% O_2_, spheroids were grown to a diameter of ∼700 μm to obtain a larger hypoxic core and give an increased likelihood of a visible signal. Compound **3** displayed a less linear increase in fluorescence toward the center of the spheroids, but a 1.5-fold increase was measured in region 1 compared to the peripheral region of spheroid 6 (Figure 5C). As compound **4** was seen to have a turn-on response at the most severe levels of hypoxia, spheroids were again grown to a diameter of ∼700 μm. Compound **4** showed a steady increase in fluorescence intensity toward the center of the spheroid—region 1, with a maximum 1.7-fold increase observed in the central region of the spheroid (Figure 6D). These data demonstrate compounds **2**, **3**, and **4** can detect regions of hypoxia within spheroids, with compounds **2** and **4** able to image different levels of hypoxia in this setting. Compound **2** can detect milder hypoxia, similar to that imaged by pimonidazole, while compound **4** detects only extreme hypoxia as is also the case for EF5.

**Figure 7:**
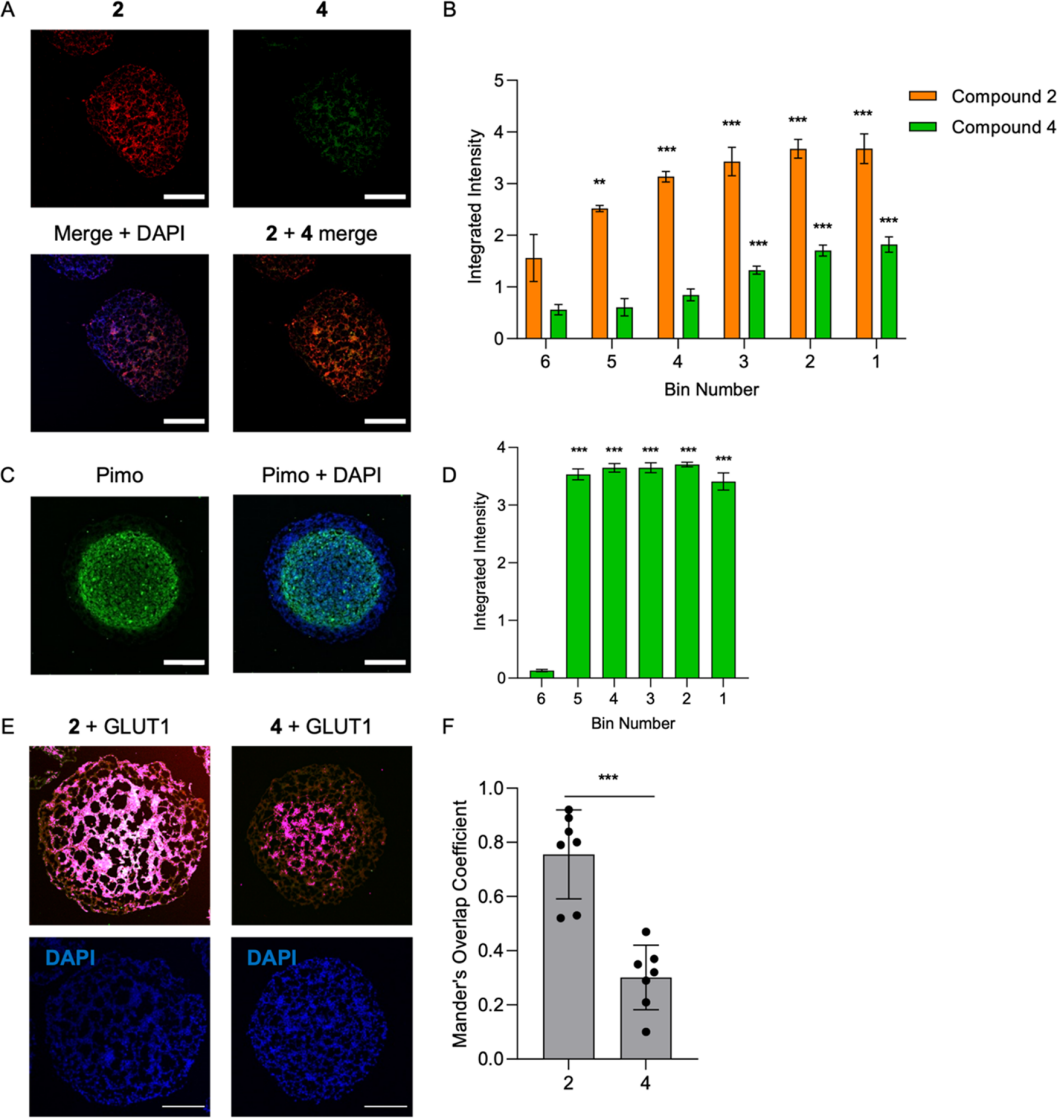
Combined use of probes **2** and **4** in 3D cell culture spheroid models. Spheroids grown from HCT116 cells were incubated with compounds **2** and **4** (40 μM) or pimonidazole (100 μM) for 1.5 h. Scale bar = 200 μm. (A) Representative cryo-sectioned spheroid incubated with compounds **2** and **4** is shown, demonstrating an increase in fluorescence intensity toward the hypoxic center of the spheroids. (B) Quantification of the fluorescence intensity of compounds **2** and **4** in spheroids treated as in panel A (*n* = 7). (C) Representative image of pimonidazole staining. (D) Quantification of the fluorescence intensity of panel C (*n* = 7). Error bars represent SD. *p < 0.05, **p < 0.01, and ***p < 0.001. (E) Representative images showing areas of overlap between GLUT1 and the probe indicated, with overlap indicated highlighted in pink and white. (F) Probe **2** has a Mander’s overlap coefficient value of 0.84 ± 0.16 with GLUT1, while probe **4** has a Mander’s overlap coefficient value of 0.34 ± 0.12 with GLUT1 (*n* = 7). Error bars represent SD. *p < 0.05, **p < 0.01, and ***p < 0.001.

Having demonstrated that compounds **2** and **4** can be used to image different levels of oxygen in both 2D and 3D cell culture systems individually, the probes were used in combination on spheroids to further demonstrate their ability to measure different levels of oxygen. Spheroids were grown to an average diameter of 600 μm. Compound **2** displayed significant fluorescence intensity starting from the peripheral region 5 and increasing toward the center of the spheroid—region 1. Compound **4** showed a steady increase in fluorescence intensity starting from the peripheral region 3 toward the center of the spheroid—region 1 (Figure 7A,B). For reference, staining with pimonidazole was also carried out (Figure 7C,D). Pimonidazole staining of HCT116 spheroids shows a large central region of hypoxia, suggesting that in this model, regions of <2% O_2_ are not restricted to a region within the core of the spheroid and extend far out into the peripheral regions (Figure 7C). To further confirm that probes **2** and **4** image different levels of hypoxia, we also conducted colocalization studies of each probe with glucose transporter 1 (GLUT1) expression imaged using antibodies (Figures 7E and S23). GLUT1 is a hypoxia-inducible factor 1 (HIF-1) target gene that is a marker for hypoxia with upregulation starting in moderate hypoxia and becoming more pronounced in more extreme hypoxia.^49^ Image analyses to determine the overlap of the indicated released dye and the GLUT1 antibody are shown in light pink and white (Figure 7E). The resorufin released by probe **2** has a Mander’s overlap coefficient of 0.84 ± 0.16, indicating significantly higher overlap with GLUT1 than the Me-Tokyo Green released by probe **4**, which has a Mander’s overlap coefficient of 0.37 ± 0.12 (Figure 7F).^50,51^ These results are consistent with probe **2** imaging milder levels of hypoxia, in which GLUT1 levels start to increase, while probe **4** only images more extreme hypoxia and consequently has less overlap with GLUT1.

## Conclusions

In summary, we have developed an operationally simple protocol to prepare a palette of hypoxia-sensing fluorescent probes (**1–4**) with a range of emission colors through red, orange, and green. These probes are activated reductively under either chemical or enzymatic conditions to release their fluorescent reporters. The rates at which the fluorescent reporters are released correlate with the p*K*_a_ of the reporters and are accelerated by the addition of a benzylic methyl group on the indolequinone bioreductive group. Compound **1** displayed unexpected redox properties resulting from the parent dye DDAO undergoing enzymatic reduction in hypoxia. However, the reduced form of DDAO (**13**) can be reoxidized when exposed to 21% O_2_, meaning that this probe is useful for detecting mild hypoxia in certain settings. The reversibility displayed by DDAO could form the basis for the design of more dynamic dyes capable of imaging fluctuations in hypoxia in real time. The resorufin-based probe **2** was activated in conditions of 4% O_2_ and lower, while the Me-Tokyo Green-based probe **4** was only activated in severe hypoxia—0.5% O_2_ and less. Application of these compounds in spheroids revealed that compound **2** images similar levels of hypoxia to pimonidazole, while compound **4** images more extreme hypoxia in a manner analogous to EF5. Therefore, compounds **2** and **4** represent convenient small-molecule alternatives to image hypoxia in cells and spheroids without the need for secondary detection steps involving immunostaining. Most importantly, probes **2** and **4** could be used in combination to enable two color imaging of the heterogeneous hypoxic environments within spheroids. As the indolequinone group has previously been used as a component of hypoxia-activated prodrugs *in vivo*,^52^ it is possible that this technology could be applied to imaging of hypoxia in an *in vivo* setting. However, this is likely to require optimization of the photophysical properties of the attached fluorophores or combination with another imaging modality that enables great depth penetration.

## Supporting Information Available

UV/vis spectra, cyclic voltammetry, procedures and HPLC data for chemical or enzymatic reduction of the probes, stability analysis of the probes, the biological methods, the chemical experimental details, NMR spectra, and HPLC traces (PDF)
