# Supplementary material for: Two Color Imaging of Different Hypoxia Levels in Cancer Cells
Source: J Am Chem Soc. 2023 Jan 19;145(4):2572–83. doi: 10.1021/jacs.2c12493 (PMC9896549; doi:10.1021/jacs.2c12493)
Supplement: Supplementary file 1 — ja2c12493_si_001.pdf [file ja2c12493_si_001.pdf]

Two color imaging of different hypoxia levels in cancer cells

Antoine L. D. Wallabregue,[1†] Hannah Bolland,[2†] Stephen Faulkner,[1] Ester M. Hammond,*[2] and Stuart J. Conway*[1@]

[1]Department of Chemistry, Chemistry Research Laboratory, University of Oxford, Mansfield Road, Oxford, OX1 3TA, UK.

[2]Oxford Institute for Radiation Oncology, Department of Oncology, University of Oxford, Old Road Campus Research Building, Oxford, OX3 7DQ, UK.

Supporting Information

[†]These authors contributed equally to this work.

[*]Corresponding authors

Stuart J. Conway

stuart.conway@chem.ox.ac.uk

ORCID – 0000-0002-5148-117X

Ester M. Hammond

ester.hammond@oncology.ox.ac.uk

ORCID – 0000-0002-2335-3146

Antoine L. D. Wallabregue

ORCID – 0000-0002-3181-8636

Hannah Bolland

ORCID – 0000-0003-3740-1046

Stephen Faulkner

ORCID – 0000-0003-1878-5857

[@]Lead contact

Contents

1. UV-visible and fluorescence spectra of indolequinone-based probes 3
2. Cyclic voltammetry 5
3. Chemical reduction of indolequinone-based probes 6
General procedure 6
Positive control procedure 6
Control 1 procedure 6
Control 2 procedure 6
4. Enzymatic or chemical reduction of the indolequinone-based probes 11
General procedure for fluorescence-based assay 11
Fluorescence-based assay positive control procedure 11
Fluorescence-based assay normoxia control procedure 11
General procedure for HPLC-based assay 11
HPLC-based assay positive control procedure 12
HPLC-based assay normoxia control procedure 12
Analysis of GSH action on the fluorescent probes 12
5. Stability and selectivity of the indolequinone-based probes (compounds 1-4) 21
6. Biological methods 24
Cell Lines and Reagents 24
Hypoxic Treatments 24
Immunofluorescence 24
Spheroid Culture 24
Spheroid Immunofluorescence Microscopy 25
Spheroid Analysis 25
7. Chemistry experimental section 30
8. NMR spectra 42
9. HPLC Traces 47

Supplementary Information

1. UV-visible and fluorescence spectra of indolequinone-based probes

The absorption spectra of the reporters (DDAO, resorufin, Me-Tokyo green and 7-hydroxy-4-methylcoumarin) display significant changes upon alkylation with indolequinones (Figure S1). While DDAO exhibits absorption maxima at 275 (ε=7730 $M^{-1}cm^{-1}$), 471 (ε=14084 $M^{-1}cm^{-1}$), and 631 nm (ε=5000 $M^{-1}cm^{-1}$), compound **1** exhibits a bathochromic (red) shift of the first absorption peak to 279 nm along with an increase in absorbance (hyperchromic shift) (ε=24700 $M^{-1}cm^{-1}$), a hypsochromic (blue) shift of the second absorption peak to 468 nm along with an hyperchromic shift (ε=21800 $M^{-1}cm^{-1}$) and notably, the loss of the absorption peak at 631 nm (Figure S1A). A peak at 307 nm (ε=22750 $M^{-1}cm^{-1}$) and a broad peak at 587 nm (ε=112895 $M^{-1}cm^{-1}$) were observed for resorufin. Probe **2** exhibits two moderately strong peaks at 279 nm (ε=24700 $M^{-1}cm^{-1}$) and 455 nm (ε=31309 $M^{-1}cm^{-1}$) and the loss of the absorption maximum at 587 nm (Figure S1C**)**. Me-Tokyo green shows a peak at 300 nm (ε=6290 $M^{-1}cm^{-1}$) and a broad peak at 455 nm (ε=29800 $M^{-1}cm^{-1}$), and the introduction of indolequinones **5** or **6** led to the same effects and the absorption spectra are virtually superimposable (Figure S1E&G**)**. Both probes **3** and **4** exhibit a peak at 277 nm (ε=34300 $M^{-1}cm^{-1}$), a peak at 354 nm (ε=34300 $M^{-1}cm^{-1}$) and a broad and complex peak displaying three maxima at 430 nm (ε=21930 $M^{-1}cm^{-1}$), 455 nm (ε=29800 $M^{-1}cm^{-1}$) and 480 nm (ε=16581 $M^{-1}cm^{-1}$). Overall, these data indicate that the spectroscopic properties of probes **1**-**4** and their corresponding reporters are different, and these differences result from the presence of the indolequinone groups.

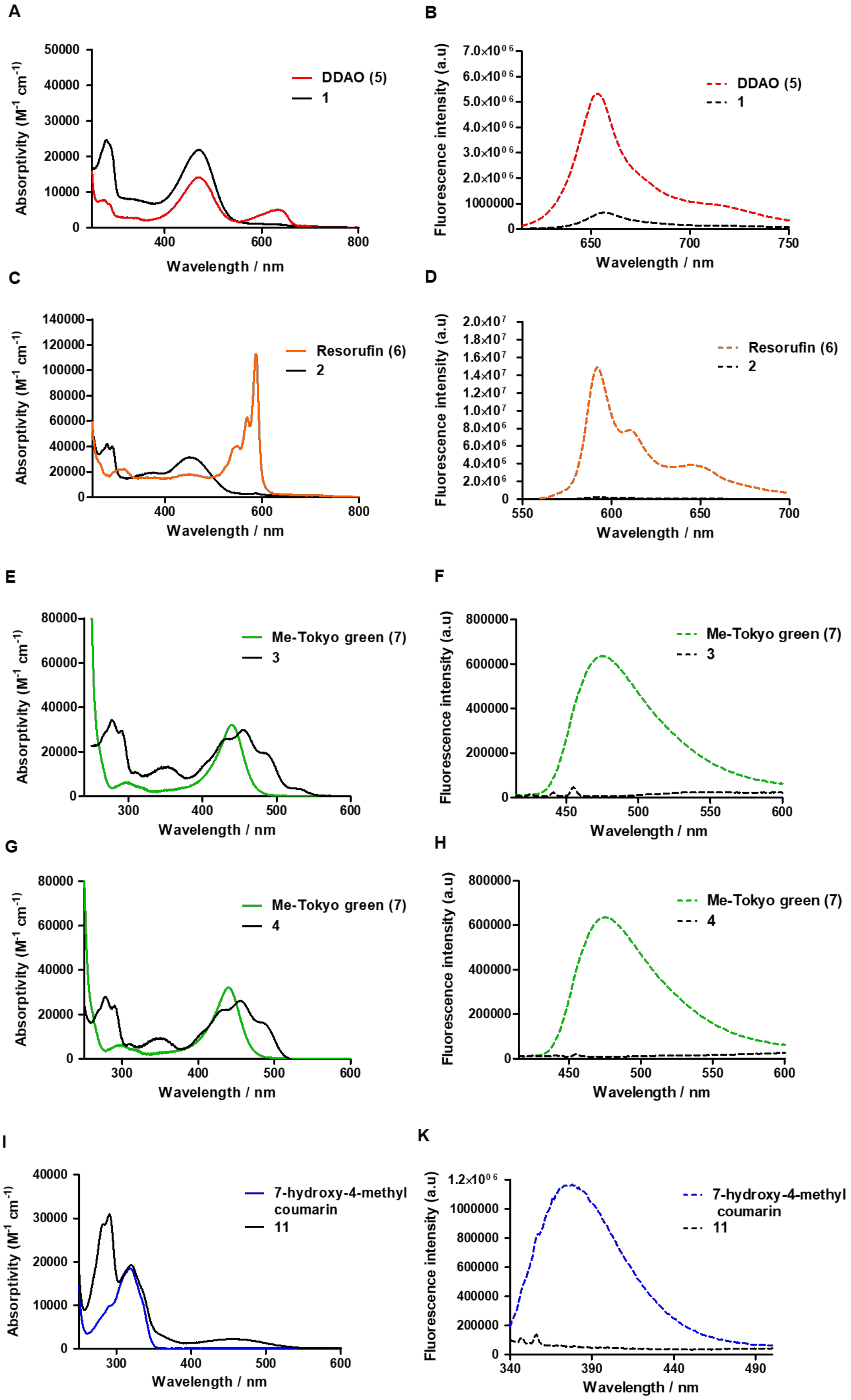

Figure S1. UV-visible (**A**, **C**, **E**, **G** and **I**) and fluorescence (**B**, **D**, **F**, **H** and **K**) spectra of indolequinone-based derivatives **1-4** and **11** and their corresponding 'free' fluorophores [DDAO (**5**), resorufin (**6**), Me-Tokyo (**7**) green, and 7-hydroxy-4-methyl coumarin, respectively] in acetonitrile under ambient conditions. c = 4 µM in **A** and **B**, c = 1 µM for **C** to **H** and c = 5 µM in **I** and **K**. Fluorescence intensity data were collected after excitation at 600 nm, slits 5, 5 nm (**B**); excitation at 545 nm, slits 3, 3 nm (**D**), after excitation at 400 nm, slits 2, 2 nm (**F** and **H**), and excitation at 322 nm slits 1, 1 nm (**K**)

2. Cyclic voltammetry

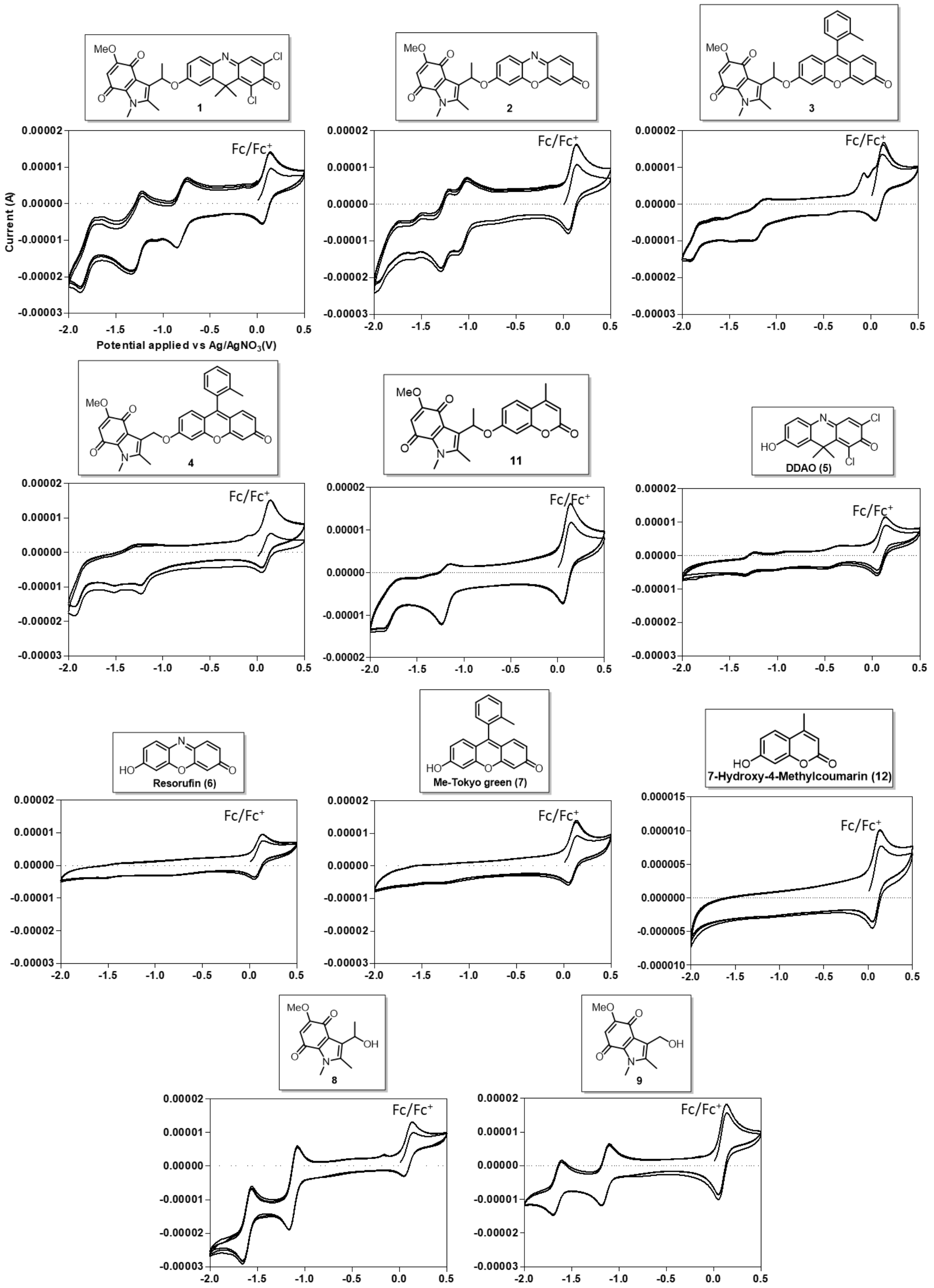

Figure S2. Cyclic voltammograms of indolequinone-based derivatives **1-4** and **11**, fluorophores (**5**-**7** and **12**) and quinones **8** and **9** recorded in CH_3CN solutions with 0.1 M tetrabutylammonium hexafluorophosphate ($TBAPF_6$) as supporting electrolyte under argon and recorded at a scan rate of 0.1 V s^{-1} against silver chloride. Ferrocene is used as an internal standard (Fc/Fc^+). Chemical structures of the measured indolequinone-based probes are shown.

3. Chemical reduction of indolequinone-based probes

General procedure: To a round-bottom flask containing MilliQ water (800 µL, pH 7.0) and acetonitrile (200 µL), was added a solution of indolequinone-based probe (200 µL, final concentration 0.167 mM) under argon. An aliquot (400 µL) was collected from the reaction solution and filtered using a syringe filter (Gilson PTFE-4-4, size × porosity: 13 mm × 0.20 µm). The filter was washed with acetonitrile (200 µL) and the flow-through was combined with the aliquot and analyzed by HPLC (this sample serves as $T = 0$ (where T = time). Next, sodium dithionite (522 µg, 3 µmol) was added to the solution and the mixture was stirred at room temperature under argon. An aliquot (400 µL) was collected at the indicated time, filtered using a syringe filter and the filter washed with acetonitrile (200 µL). The flow-through was combined with the aliquot and analyzed using HPLC or LCMS.

Positive control procedure: To a round-bottom flask containing MilliQ water (800 µL, pH 7.0) and acetonitrile (200 µL), was added a solution of the fluorescent probe (200 µL, final concentration 0.167 mM) under argon. An aliquot (400 µL) was collected from the reaction solution and filtered using a syringe filter. The filter was washed with acetonitrile (200 µL) and the flow-through was combined with the aliquot and analyzed using HPLC (this serves as $T = 0$ (where T = time). After sodium dithionite (522 µg, 3 µmol) was added to the solution and the mixture was stirred at room temperature under argon. An aliquot (400 µL) was collected at the indicated time, filtered using a syringe filter and the filter was washed with acetonitrile (200 µL). The flow-through was combined with the aliquot and analyzed using HPLC or LCMS.

Control 1 procedure: To a round-bottom flask under argon containing MilliQ water (900 µL, pH 7.0) and acetonitrile (200 µL) was added a solution of quinone (100 µL, final concentration 0.167 mM). After sodium dithionite (522 µg, 3 µmol) was added and the reaction mixture was stirred at room temperature under argon. An aliquot (400 µL) was collected at the indicated time and filtered using a syringe filter. The filter was washed with acetonitrile (200 µL) and the flow-through was combined with the aliquot and analyzed using HPLC or LCMS.

Control 2 procedure: To a round-bottom flask under argon containing MilliQ water (800 µL, pH 7.0) and acetonitrile (200 µL) was added a solution of indolequinone-based probe (200 µL, final concentration 0.167 mM). The reaction solution was stirred at room temperature under argon. An aliquot (400 µL) was collected at the indicated time and filtered using a syringe filter. The filter was washed with acetonitrile (200 µL) and the flow-through was combined with the aliquot and analyzed using HPLC or LCMS.

A

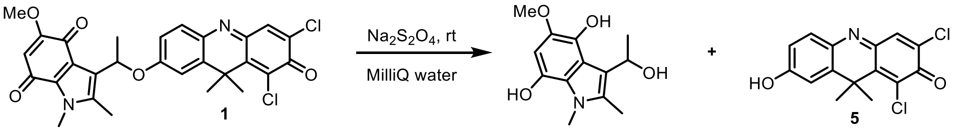

B

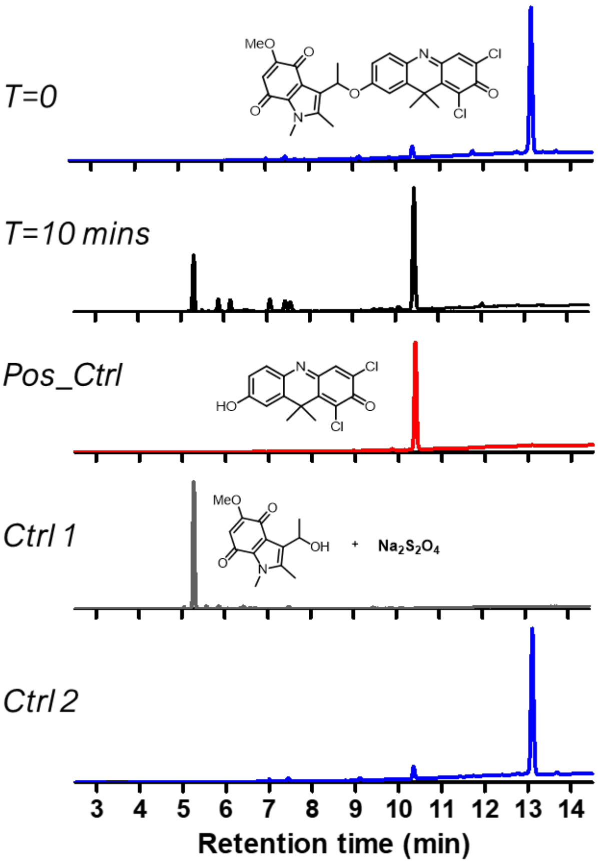

Figure S3. Compound 1 can be reduced to its corresponding fluorescent reporter DDAO (5). (**A**) Chemical reduction of compound **1** to **5** using sodium dithionite. (**B**) Compound **1** was treated with sodium dithionite in MilliQ water under an argon atmosphere (see general procedure) for 1 hour. Aliquots were taken at the time indicated and analyzed using HPLC: Perkin Elmer Dionex Acclaim™ 120 C18 column [5 µm, 120 Å, 4.6 × 150 mm]; 95:5 H_2O: MeCN → 5:95 H_2O: MeCN: H_2O with 0.1% TFA modifier, 10 min; 5 min hold; 1.0 mL min^{-1}]. Absorbance was recorded at 254 nm. The injection peak was omitted, and the chromatogram starts at 2.5 mins for clarity. Aliquot was taken at $T = 0$. Aliquot was taken at $T = 10$ mins. Positive control: DDAO (**5**) was treated with sodium dithionite (see procedure) for 10 mins after an aliquot was taken. Control 1: compound **8** was treated with sodium dithionite (see procedure) for 30 mins after an aliquot was taken. Control 2: compound **1** was incubated in MilliQ water (see procedure) for 30 mins after an aliquot was taken. n=3.

A

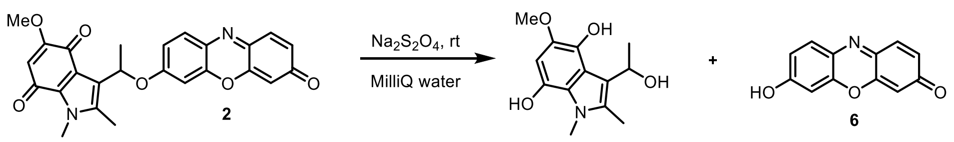

B

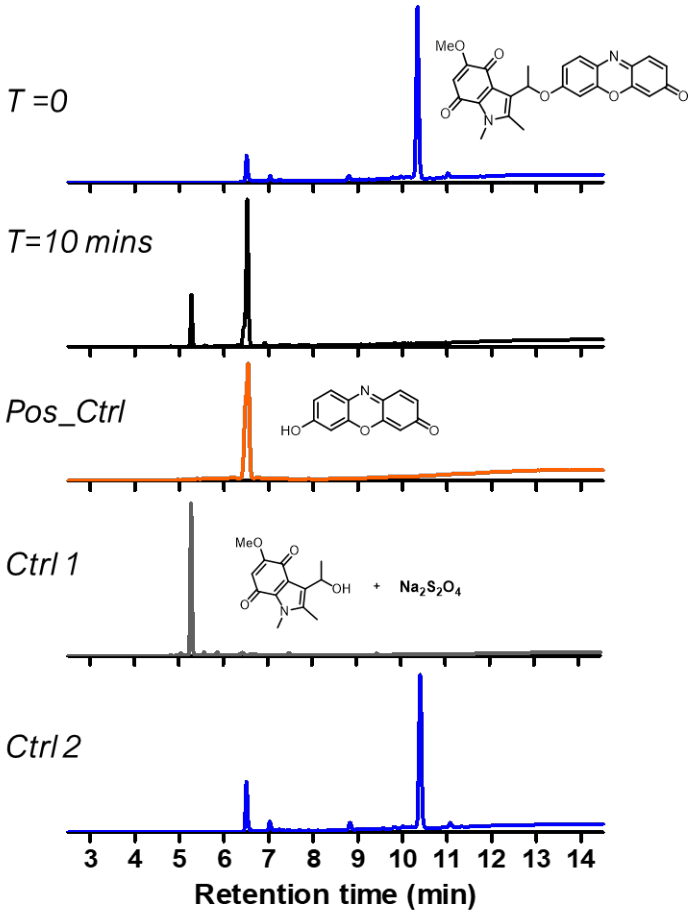

Figure S4. Compound 2 can be reduced to its corresponding fluorescent reporter resorufin (6). (**A**) Chemical reduction of compound **2** to **6** using sodium dithionite. (**B**) Compound **2** was treated with sodium dithionite in MilliQ water under an argon atmosphere (see general procedure) for 1 hour. Aliquots were taken at the time indicated and analyzed using HPLC: Perkin Elmer Dionex Acclaim™ 120 C18 column [5 µm, 120 Å, 4.6 × 150 mm]; [95:5 H_2O: MeCN → 5:95 H_2O: MeCN: H_2O with 0.1% TFA modifier, 10 min; 5 min hold; 1.0 mL min^{-1}]. Absorbance was recorded at 254 nm. The injection peak was omitted, and the chromatogram starts at 2.5 mins for clarity. Aliquot was taken at $T = 0$. Aliquot was taken at $T = 10$ mins. Positive control: Resorufin (**6**) was treated with sodium dithionite (see procedure) for 10 mins after an aliquot was taken. Control 1: Compound **8** was treated with sodium dithionite (see procedure) for 30 mins after an aliquot was taken. Control 2: Compound **2** was incubated in MilliQ water (see procedure) for 30 mins after an aliquot was taken. n=3.

A

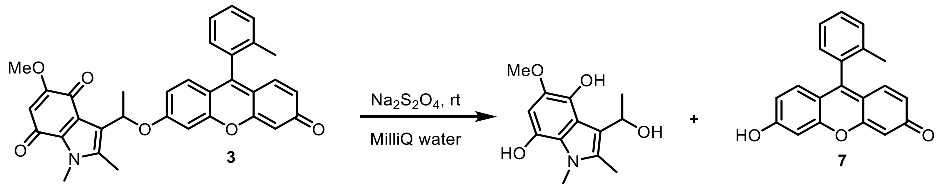

B

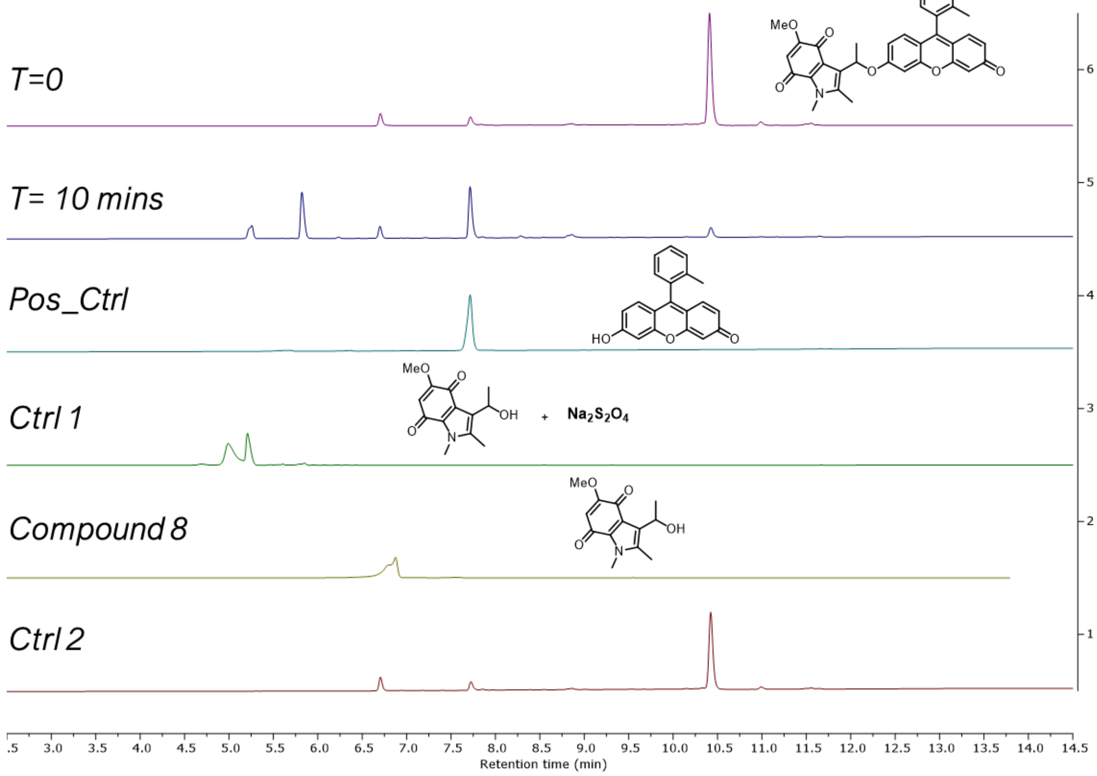

Figure S5. Compound 3 can be reduced to its corresponding fluorescent reporter Me-Tokyo green (7). (**A**) Chemical reduction of compound **3** to **7** using sodium dithionite. (**B**) Compound **3** was treated with sodium dithionite in MilliQ water under an argon atmosphere (see general procedure) for 1 hour. Aliquots were taken at the time indicated and analyzed using HPLC: Agilent 1260 Infinity II® Poroshell 120 EC-C18 column [4 µm, 4.6 × 100 mm]; [95:5 H_2O: MeCN → 5:95 H_2O: MeCN: H_2O with 0.1% FA modifier, 10 min; 5 min hold; 1 mL min^{-1}]. Absorbance was recorded at 280 nm. The injection peak was omitted, and the chromatogram starts at 2.5 mins for clarity. (**A**) Aliquot was taken at *T* = 0. Aliquot was taken at *T* = 10 mins. Positive control: Me-Tokyo green (**7**) was treated with sodium dithionite (see procedure) for 10 mins after an aliquot was taken. Control 1: Compound **8** was treated with sodium dithionite (see procedure) for 30 mins after what an aliquot was taken. Compound **8**. Control 2: Compound **3** was incubated in MilliQ water (see procedure) for over 10 mins after an aliquot was taken. n=3.

A

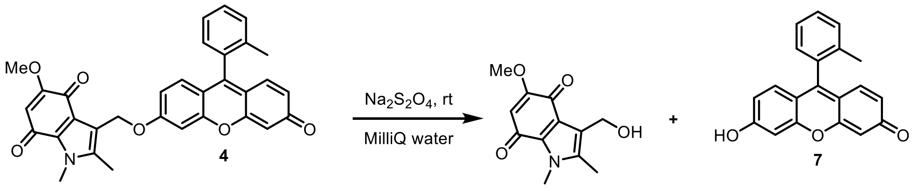

B

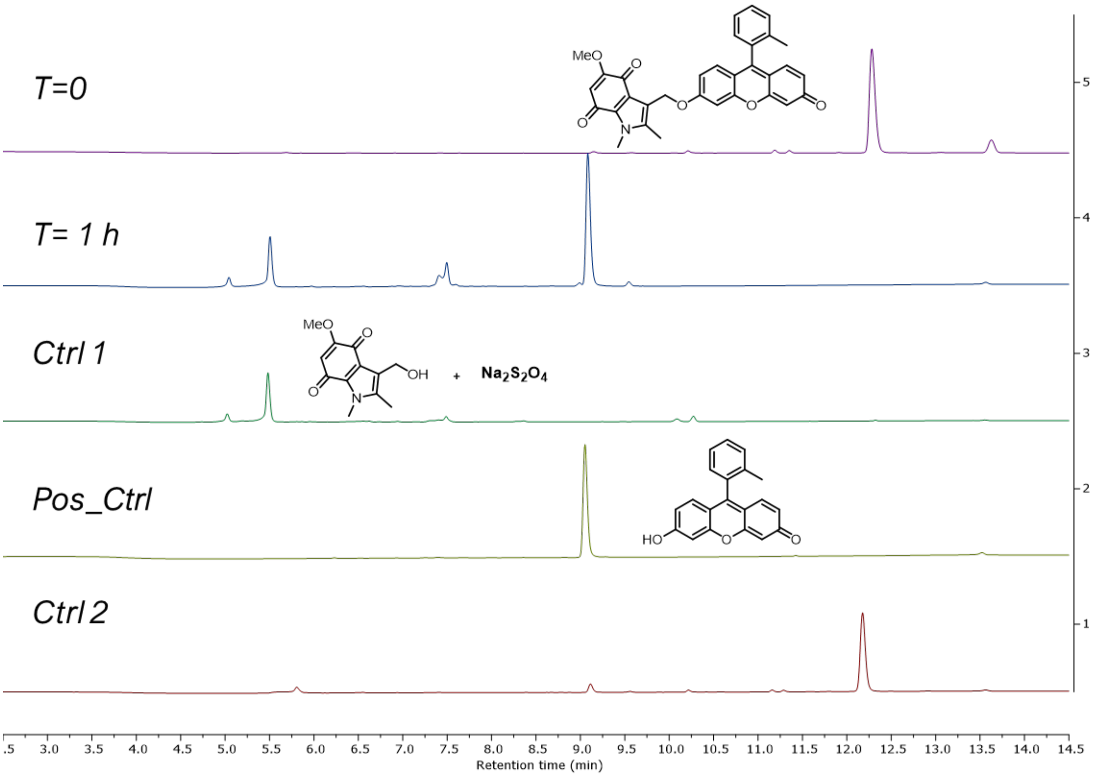

Figure S6. Compound 4 can be reduced to its corresponding fluorescent reporter Me-Tokyo green (7). (**A**) Chemical reduction of compound **4** to **7** using sodium dithionite. (**B**) Compound **4** was treated with sodium dithionite in MilliQ water under an argon atmosphere (see general procedure) for 1 hour. Aliquots were taken at the time indicated and analyzed using LCMS: Agilent 1260 Infinity II® ZORBAX SB C18 column [1.8 µm, 2.1 × 50 mm]; [95:5 H_2O: MeCN → 5:95 H_2O: MeCN: H_2O with 0.1% FA modifier, 10 min; 5 min hold; 0.4 mL min^{-1}]. Absorbance was recorded at 280 nm. The injection peak was omitted, and the chromatogram started at 2.5 mins for clarity. Aliquot was taken at *T* = 0. Aliquot was taken at *T* = 1 hour. Control 1: Compound **9** was treated with sodium dithionite (see procedure) for 30 mins after what an aliquot was taken. Positive control: Me-Tokyo green (**7**) was treated with sodium dithionite (see procedure) for 1 hour after what an aliquot was taken. Control 2: Compound **4** was incubated in MilliQ water (see procedure) for 1 hour after an aliquot was taken. n=3.

4. Enzymatic or chemical reduction of the indolequinone-based probes

The oxygen-dependent enzymatic reduction assays were run in a RUSKINN InvivO2® 400 hypoxic Workstation at 37 °C with 0.1% oxygen and 99.9% nitrogen concentrations unless otherwise stated. NADPH-cytochrome P450 reductase (PH51, expressed in *E. coli* containing cDNA for recombinant NADPH-P450 reductase, concentration 0.602 mg/mL; purity ≥90%) was purchased from 2Be Scientific and β-nicotinamide adenine dinucleotide 2′-phosphate reduced tetrasodium salt hydrate (β-NADPH) from Sigma Aldrich. The buffers used in the assays were equilibrated under the conditions stated above in the hypoxic Workstation for 18 hours before use. HPLC and/or fluorimetry were used as readouts for reaction analysis.

General procedure for fluorescence-based assay: To a 3 mL quartz cuvette on ice (0-4 °C) containing phosphate buffer (5 mM, pH 7.4, 2960 µL) and a solution of β-NADPH (30 µL, of a 1 mM stock solution in phosphate buffer), was added a solution of indolequinone-based probe (3 µL, of a 1 mM stock solution in acetonitrile). The cuvette was sealed, and the UV-visible and fluorescence spectra were measured according to the indicated parameters, this serves as $T = 0$ (where T = time). Next, a solution of NADPH-cytochrome P450 reductase in phosphate buffer (5 mM, pH 7.4, final concentration 0.72 ng/µL) was added to the reaction mixture in the hypoxia chamber, and the cuvette was left in the hypoxia chamber (at this point, the final concentrations of β-NADPH and indolequinone-based probe were 20 µM and 1 µM, respectively). The UV-visible and fluorescence spectra of the reaction were recorded at the given time point.

Fluorescence-based assay positive control procedure: To a 3 mL quartz cuvette on ice (0-4 °C) containing phosphate buffer (5 mM, pH 7.4, 2960 µL) and a solution of β-NADPH (30 µL, of a 1mM stock solution in phosphate buffer), was added a solution of fluorescent probe (3 µL, of a 1 mM stock solution in acetonitrile). The cuvette was sealed, and the UV-visible and fluorescence spectra were measured according to the indicated parameters, this serves as $T = 0$ (where T = time). Next, a solution of NADPH-cytochrome P450 reductase in 5 mM phosphate buffer pH 7.4 (final concentration 0.72 ng/µL) was added to the reaction mixture in the hypoxia chamber, and the cuvette was left in the hypoxia chamber (at this point, the final concentrations of β-NADPH and fluorescent probe were 20 µM and 1 µM, respectively). The UV-visible and fluorescence spectra of the reaction were recorded at the given time point.

Fluorescence-based assay normoxia control procedure: To a 3 mL quartz cuvette on ice (0-4 °C) containing phosphate buffer (5 mM, pH 7.4, 2960 µL) and a solution of β-NADPH (30 µL, of a 1 mM stock solution in phosphate buffer), was added a solution of fluorescent probe (3 µL, of a 1 mM stock solution in acetonitrile). The cuvette was sealed, and the UV-visible and fluorescence spectra were measured according to the indicated parameters, this serves as $T = 0$ (where T = time). After a solution of NADPH-cytochrome P450 reductase in phosphate buffer (5 mM, pH 7.4, final concentration 0.72 ng/µL) was added to the reaction mixture, and the cuvette was left at room temperature and under ambient conditions (at this point, the final concentrations of β-NADPH and indolequinone-based probe were 20 µM and 1 µM, respectively). The UV-visible and fluorescence spectra of the reaction were recorded at a defined time point

General procedure for HPLC-based assay: To a 2 mL Eppendorf tube on ice (0-4 °C) containing phosphate buffer (5 mM, pH 7.4, 1919 µL) and NADPH-cytochrome P450 reductase (31 µL, final concentration 3.2 ng/µL),

was added a solution of indolequinone-based probe (50 µL, final concentration 25 µM). An aliquot (500 µL) was collected from the reaction solution and filtered using a syringe filter (Gilson PTFE-4-4, size × porosity: 13 mm × 0.20 µm). The syringe filter was washed with acetonitrile (50 µL) and the flow-through was combined with the aliquot and analyzed using HPLC, this serves as $T = 0$ (where T = time). After β-NADPH (1 mg, 1.1 µmol) was added and the mixture was left in the hypoxia chamber. An aliquot (500 µL) was collected at the indicated time, filtered using a syringe filter and the syringe filter was washed with acetonitrile (50 µL). The flow-through was combined with the aliquot and analyzed using HPLC.

HPLC-based assay positive control procedure: To a 2 mL Eppendorf tube on ice (0-4 °C) containing phosphate buffer (5 mM, pH 7.4, 1919 µL) and NADPH-cytochrome P450 reductase (31 µL, final concentration 3.2 ng/µL), was added a solution of fluorescent probe (50 µL, final concentration 25 µM). An aliquot (500 µL) was collected from the reaction solution and filtered using a syringe filter (Gilson PTFE-4-4, size × porosity: 13 mm × 0.20 µm). The syringe filter was washed with acetonitrile (50 µL) and the flow-through was combined with the aliquot and analyzed using HPLC, this serves as $T = 0$ (where T = time). After β-NADPH (1 mg, 1.1 µmol) was added and the mixture was left in the hypoxia chamber. An aliquot (500 µL) was collected at the indicated time, filtered using a syringe filter and the syringe filter was washed with acetonitrile (50 µL). The flow-through was combined with the aliquot and analyzed using HPLC.

HPLC-based assay normoxia control procedure: To a 2 mL Eppendorf tube on ice (0-4 °C) containing phosphate buffer (5 mM, pH 7.4, 1919 µL) and NADPH-cytochrome P450 reductase (31 µL, final concentration 3.2 ng/µL), was added a solution of indolequinone-based probe (50 µL, final concentration 25 µM). An aliquot (500 µL) was collected from the reaction solution and filtered using a syringe filter (Gilson PTFE-4-4, size × porosity: 13 mm × 0.20 µm). The syringe filter was washed with acetonitrile (50 µL) and the flow-through was combined with the aliquot and analyzed using HPLC (this will serve as $T = 0$ (where T = time). After β-NADPH (1 mg, 1.1 µmol) was added and the mixture was left at 37 °C, at ambient conditions. An aliquot (500 µL) was collected at the indicated time, filtered using a syringe filter and the syringe filter was washed with acetonitrile (50 µL). The flow-through was combined with the aliquot and analyzed using HPLC.

Analysis of GSH action on the fluorescent probes: To a 2 mL Eppendorf on ice (0-4 °C) containing buffer (700 µL) and L-glutathione (250 µL, of a 20 mM stock solution in buffer, final concentration 5 mM) was added a solution of probe (50 µL, of a 1 mM stock solution, final concentration 50 µM). Two aliquots (2 × 60 µL) were collected from the reaction solution, the first was diluted with buffer (2940 µL) in a quartz cuvette (final concentration 1 µM) and fluorescence spectra were measured according to the indicated parameters, and the second was analyzed using LRMS. These serve as $T = 0$ (where T = time). After aliquots were collected at the indicated time, treated as above-mentioned and analyzed by LRMS and fluorescence spectroscopy.

The absorption spectra of the enzymatic reductions were recorded at the indicated time. A time-dependent increase of the absorption bands at 571 and 493 nm for probes **2**, and **3-4**, respectively, was observed. This indicates the release of resorufin and Me-Tokyo green, respectively (Figure **S7C**-**S9C**). Concomitantly, the absorption spectra of the enzymatic reductions of **3** and **4** show a decrease of the cofactor β-NADPH absorbance (340 nm) and an increase of $NADP^+$ absorbance (260 nm), Figure S8C and S9C. These data indicate that the release of the fluorophores and the oxidation reaction of β-NADPH to $NADP^+$ take place within the bioreduction time, suggesting the reduction of the bioreductive group is mediated by NADPH-cytochrome P450 reductase activity. In contrast, only modest fluorescence and absorption intensities increase were measured in normoxia (21% O_2, Figure 3B, E and H, and Figure S7C-S9C). Overall, these data indicate that probes **2-4** release their corresponding fluorescent reporters in an oxygen-dependent manner catalyzed by NADPH-cytochrome P450 reductase activity.

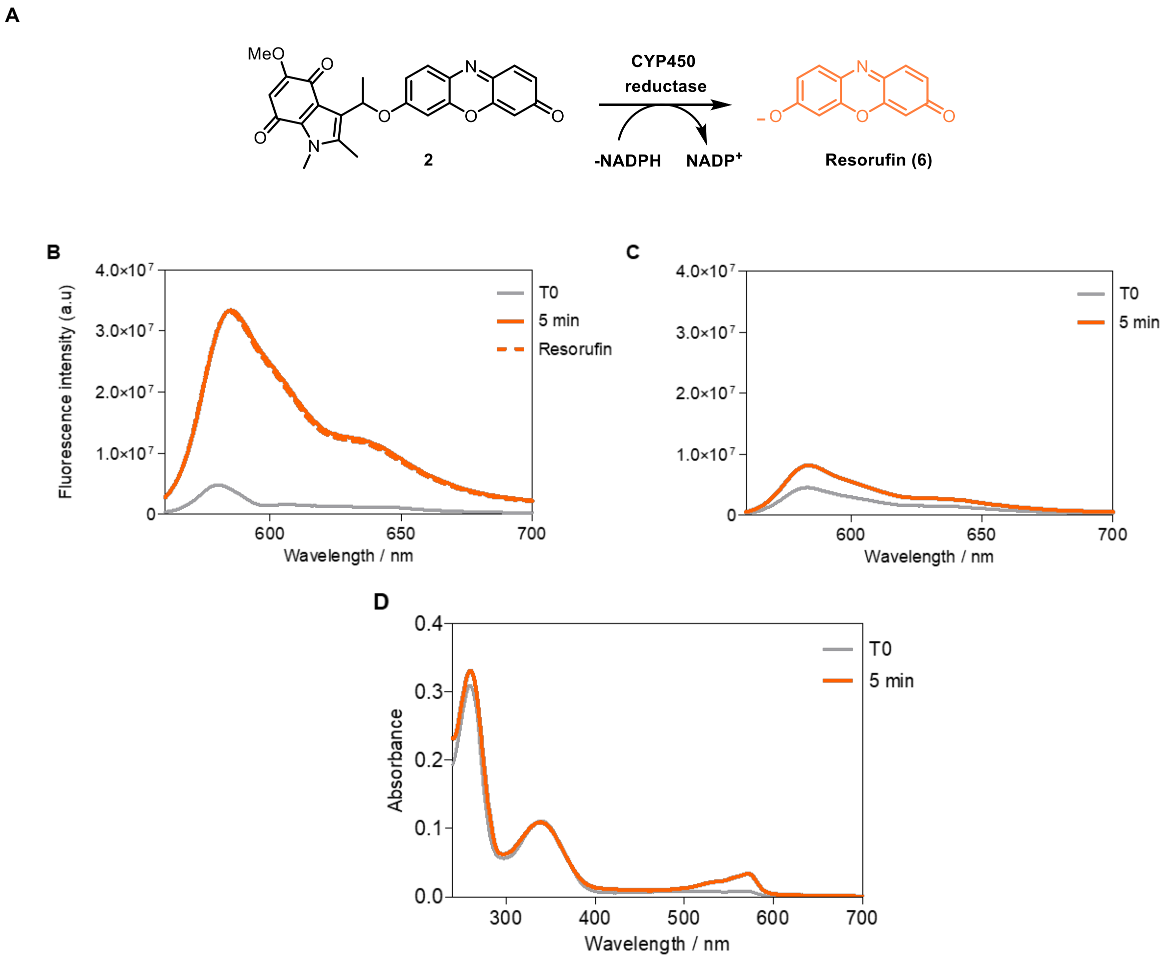

Figure S7. Compound 2 undergoes oxygen-dependent cytochrome P450 reductase (PH51)-catalyzed reduction to the corresponding fluorescent reporter resorufin (6). (**A**) Enzymatic reduction of compound **2** to **6** using PH51 and β-NADPH. (**B**) Compound **2** (1 μM) was treated with cytochrome P450 reductase (PH51, C=0.72 ng/μL) and β-NADPH (20 mM) under hypoxia (0.1% O_2) over 5 mins as described in the general procedure for fluorescence-based assay. Fluorescence intensity data were collected after the time indicated in the figure with excitation at 545 nm. Slits 3, 3 nm (a representative graph is shown, n = 3). The dashed line shows fluorescence intensity of resorufin (1 μM). Quantification = (surface area fluorescence at 5 min/surface area fluorescence of 1 μM resorufin) × 100. (**C**) Normoxic (21% O_2) conditions as in **D** (a representative graph is shown, n = 3). (**D**) Hypoxic conditions as in **A**, UV-visible spectra were collected after the time indicated in the figure (a representative graph is shown, n = 3).

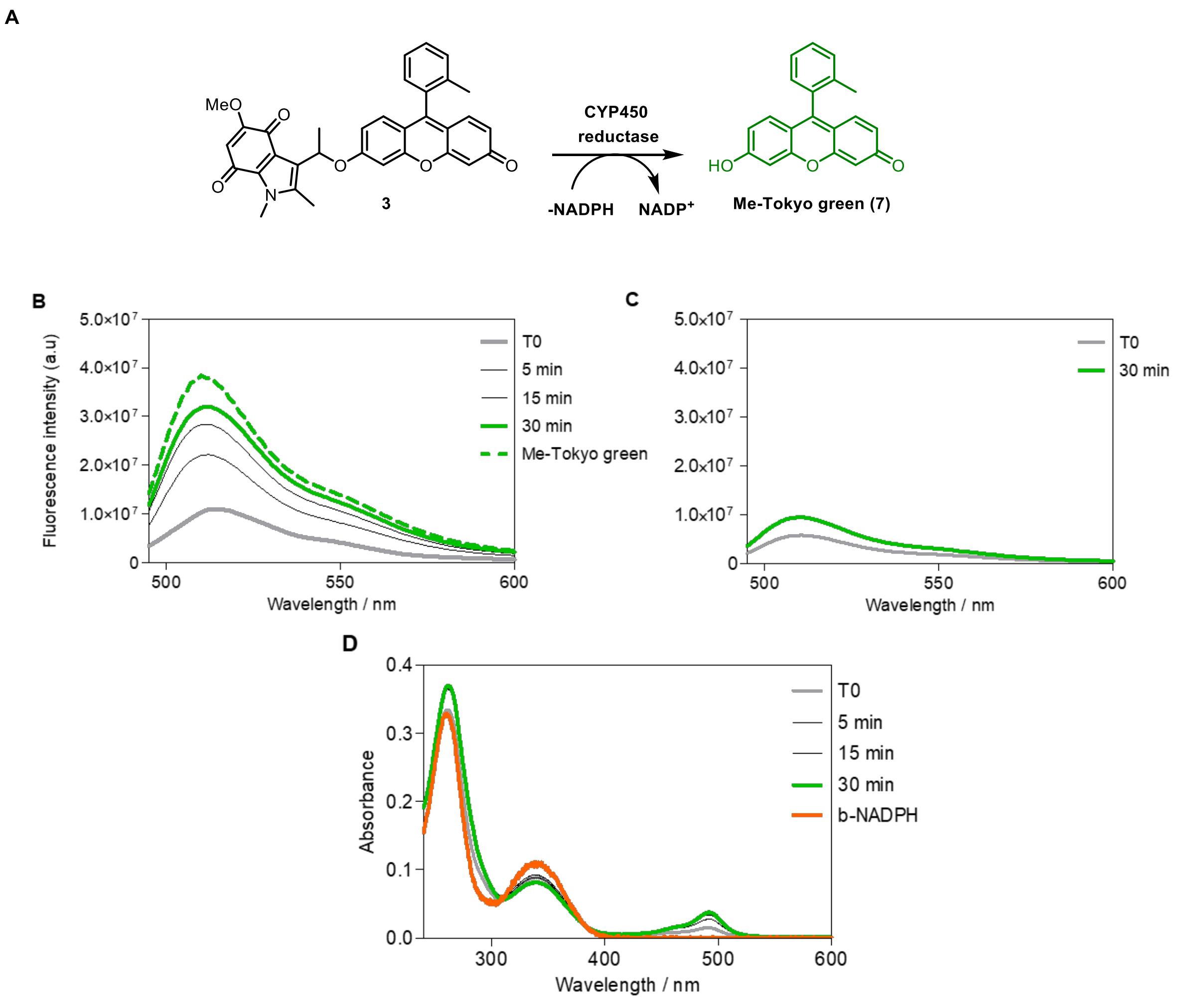

Figure S8. Compound 3 undergoes oxygen-dependent cytochrome P450 reductase (PH51)-catalyzed reduction to the corresponding fluorescent reporter Me-Tokyo green (7). (**A**) Enzymatic reduction of compound **3** to **7** using PH51 and β-NADPH. (**B**) Compound **3** (1 μM) was treated with cytochrome P450 reductase (PH51, C=0.72 ng/μL) and β-NADPH (20 mM) under hypoxia (0.1% O_2) over 150 mins as described in the general procedure for fluorescence-based assay. Fluorescence intensity data were collected after the time indicated in the figure with excitation at 480 nm. Slits 2, 2 nm (a representative graph is shown, n = 3). The dashed line shows fluorescence intensity of Me-Tokyo green (1 μM). Quantification = (surface area fluorescence at 30 min/surface area fluorescence of 1 μM Me-Tokyo green) × 100. (**C**) Normoxic (21% O_2) conditions as in **D** (a representative graph is shown, n = 3). (**D**) Hypoxic conditions as in **B**, UV-visible spectra were collected after the time indicated in the figure (a representative graph is shown, n = 3). β-NAPDH absorption spectrum (20 mM, orange line).

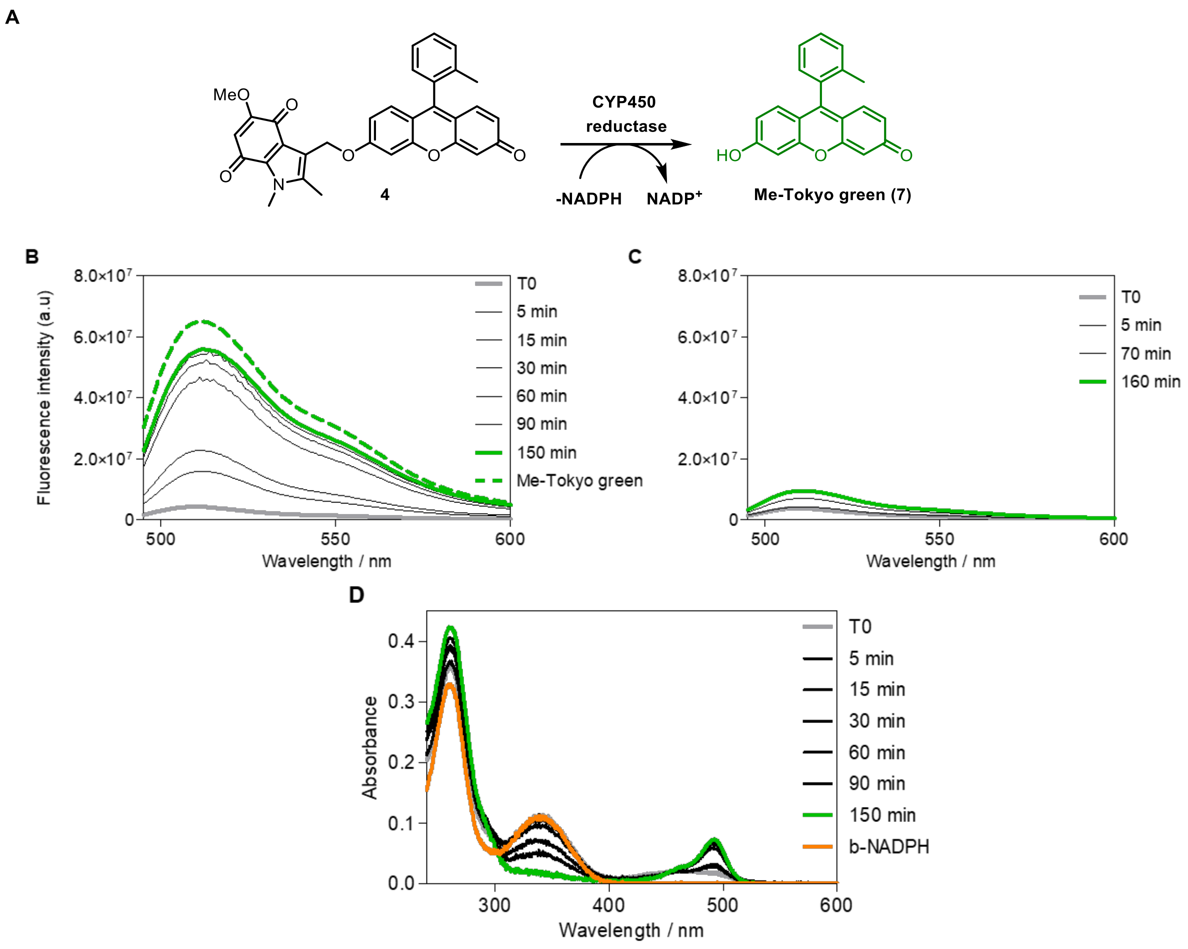

Figure S9. Compound 4 undergoes oxygen-dependent cytochrome P450 reductase (PH51)-catalyzed reduction to the corresponding fluorescent reporter Me-Tokyo green (7). (**A**) Enzymatic reduction of compound **4** to **7** using PH51 and β-NADPH. (**B**) Compound **4** (1 µM) was treated with cytochrome P450 reductase (PH51, C=0.72 ng/µL) and β-NADPH (20 mM) under hypoxia (0.1% O_2) over 150 mins as described in the general procedure for fluorescence-based assay. Fluorescence intensity data were collected after the time indicated in the figure with excitation at 480 nm. Slits 2, 2 nm (a representative graph is shown, n = 3). The dashed line shows the fluorescence intensity of Me-Tokyo green (1 µM). Quantification = (surface area fluorescence at 150 min/surface area fluorescence of 1 µM Me-Tokyo green) × 100. (**C**) Normoxic (21% O_2) conditions as in **D** (a representative graph is shown, n = 3). (**D**) Hypoxic conditions as in **B**, UV-visible spectra were collected after the time indicated in the figure (a representative graph is shown, n = 3). β-NAPDH absorption spectrum (20 mM, orange line).

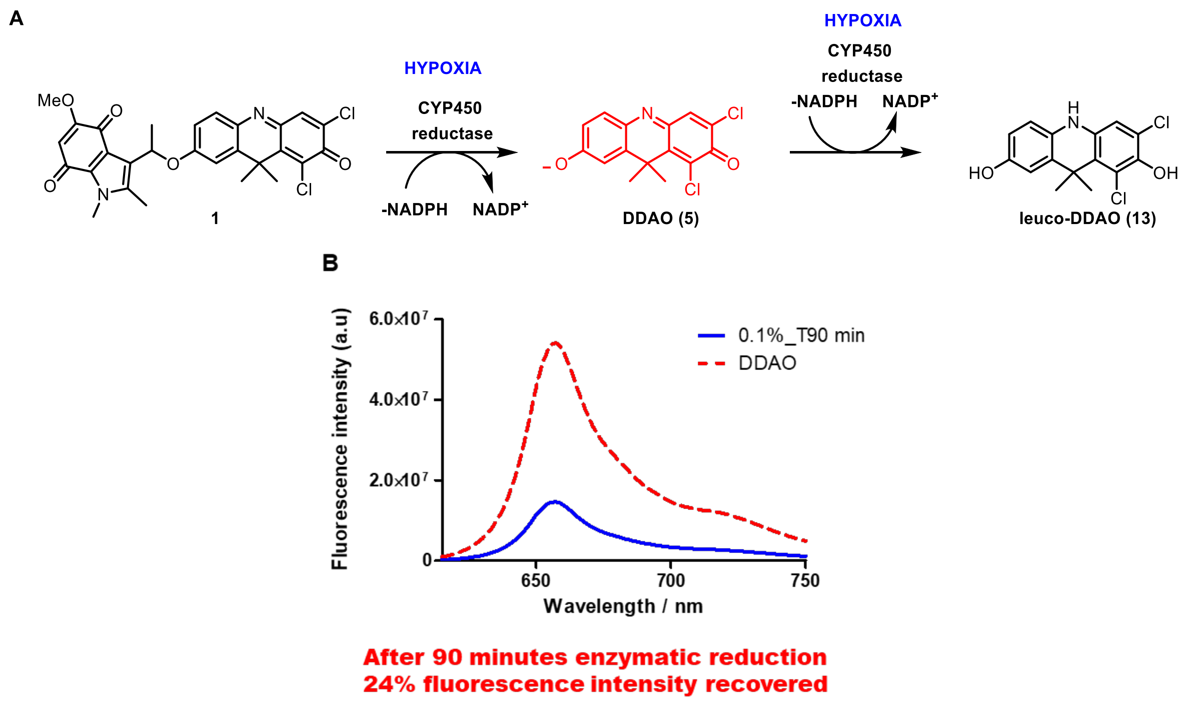

Figure S10. Compound 1 undergoes oxygen-dependent cytochrome P450 reductase (PH51)-catalyzed reduction to the corresponding non-fluorescent leuco-DDAO (13). (**A**) Enzymatic reduction of compound **1** to **13** using PH51 and β-NADPH. (**B**) Compound **1** (4 µM) was treated with cytochrome P450 reductase (PH51, C=0.72 ng/µL) and β-NADPH (20 mM) under hypoxia (0.1% O_2) over 90 mins as described in the general procedure for fluorescence-based enzymatic assay positive control (plain line). The dashed line shows the fluorescence intensity of DDAO (4 µM). Fluorescence intensity data were collected after the time indicated in the figure with excitation at 600 nm. Slits 3, 3 nm. Quantification = (surface area fluorescence at 0.1% T90 min/surface area fluorescence Positive control, DDAO) × 100. n=1.

A

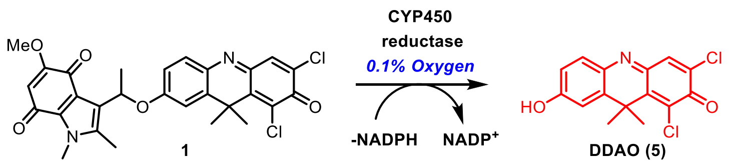

B

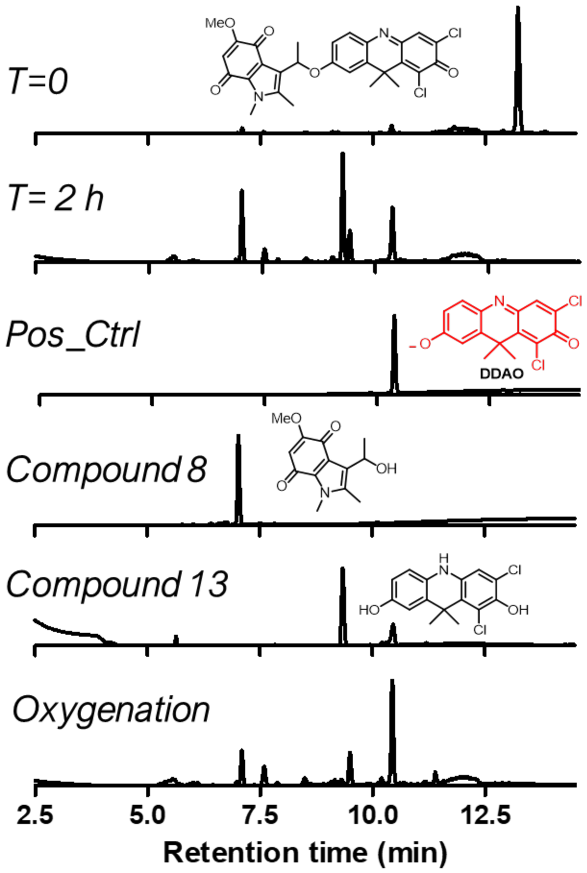

Figure S11. Compound 1 undergoes oxygen-dependent cytochrome P450 reductase (PH51)-catalyzed reduction to the corresponding fluorescent reporter DDAO (5) and the non-fluorescent leuco-DDAO (13). (**A**) Enzymatic reduction of compound **1** to **5** using PH51 and β-NADPH. (**B**) Compound **1** (25 µM) was treated with NADPH-cytochrome P450 reductase (PH51, C=3.2 ng/µL) and β-NADPH (1 mg) over 120 mins as described in the general procedure for HPLC-based assay. Aliquots were taken at the time indicated and analyzed by HPLC. Aliquot was taken at $T = 0$. Aliquot was taken at $T = 2$ hours. DDAO (25 µM). Compound **8** (25 µM). Compound **13** obtained by submitting DDAO (**5**) to conditions described in the general procedure for HPLC-based assay positive control for 120 mins after which time an aliquot was taken. Oxygenation: Compound 13 was submitted to normoxic conditions (21% O_2) at 37 °C and under ambient conditions for 40 mins, after which time an aliquot was taken. Analytical HPLC (Dionex Acclaim™ 120 C18 column [5 µm, 120 Å, 4.6 × 150 mm]; 95:5 H_2O: MeCN → 5:95 H_2O: MeCN: H_2O with 0.1% TFA modifier, 10 min; 5 min hold; 1.0 mL min^{-1}]. Absorbance was recorded at 254 nm. The injection peak was omitted, and the chromatogram started at 2.5 mins for clarity. n=3.

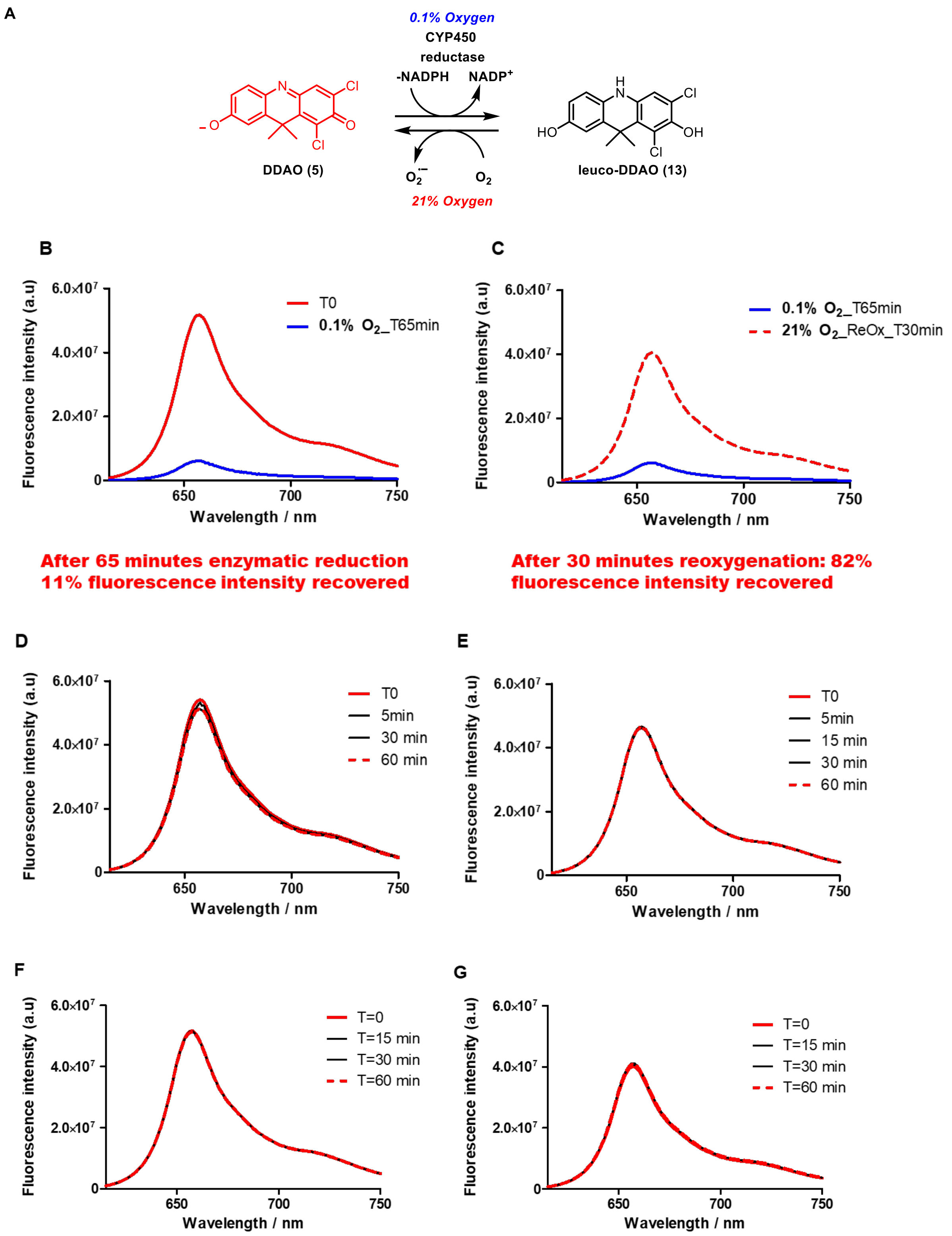

Figure S12. DDAO undergoes oxygen-dependent cytochrome P450 reductase (PH51)-catalyzed reduction to the corresponding non-fluorescent leuco-DDAO (13). (**A**) Enzymatic reduction of **DDAO** (**1**) to **13** using PH51 and β-NADPH. (**B**) DDAO (1 μM) was treated with cytochrome P450 reductase (PH51, C=0.72 ng/μL) and β-NADPH (20 mM) under hypoxia (0.1% O_2) for 65 mins, as described in the general procedure for fluorescence-based enzymatic assay positive control. Fluorescence intensity data were collected after the time indicated in the figure with excitation at 600 nm. Slits 3, 3 nm. Quantification = (surface area fluorescence at T = 0/surface area fluorescence at 0.1% T = 65 min) × 100. (**C**) Product of **B** was submitted to normoxic conditions

(21% O_2) at room temperature and under ambient conditions over 30 mins. Quantification = (surface area fluorescence at 21% O_2_Reox_T30 min/surface area fluorescence at 0.1% O_2_T65 min) × 100. (**D**) DDAO (1 μM) was treated with cytochrome P450 reductase (PH51, C=0.9 ng/μL) and β-NADPH (20 mM) under normoxia (21% O_2) over 60 mins, as described in the general procedure for fluorescence-based enzymatic assay normoxia control. Fluorescence intensity data were collected after the time indicated in the figure with excitation at 600 nm. Slits 3, 3 nm. (**E**) DDAO (1 μM) was treated with β-NADPH (20 mM) over 60 mins. (**F**) DDAO (1 μM) in phosphate buffer (pH 7.4) under hypoxia over 60 min. (**G**) DDAO (1 μM) in phosphate buffer (pH 7.4) under normoxia over 60 min. Fluorescence intensity data were collected after the time indicated in the figure with excitation at 600 nm. Slits 3, 3 nm. n=1

A

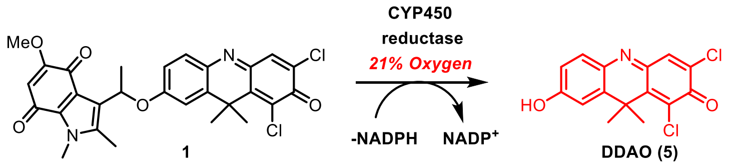

B

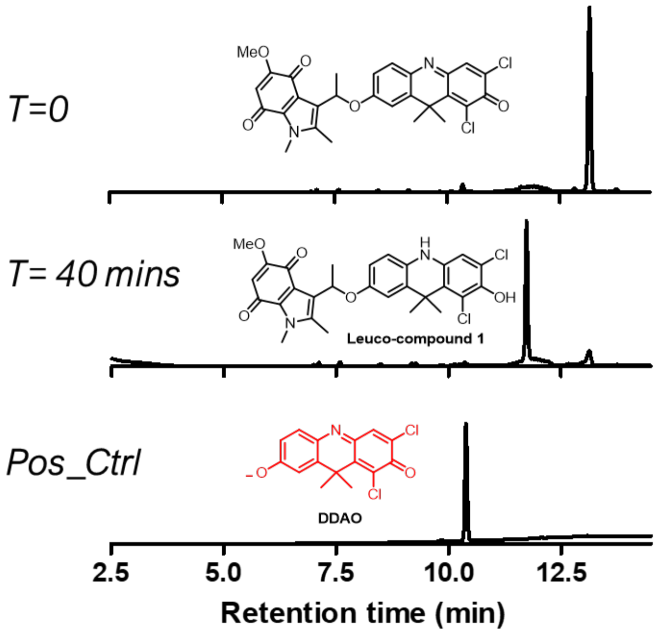

C

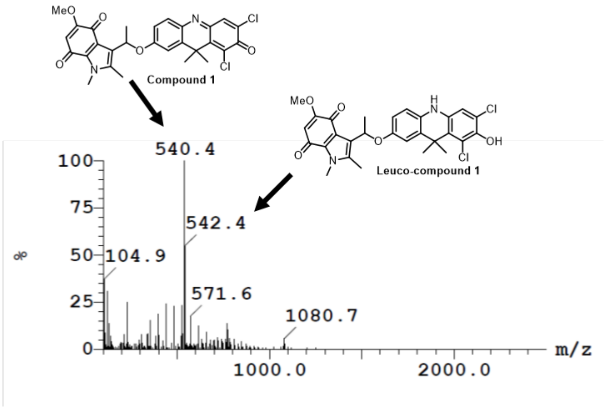

Figure S13. Compound 1 undergoes cytochrome P450 reductase (PH51)-catalyzed reduction to the non-fluorescent leuco-DDAO under normoxia. (**A**) Enzymatic reduction of **DDAO** (**1**) to **5** using PH51 and β-NADPH. (**B**) Compound **1** (25 µM) was treated with NADPH-cytochrome P450 reductase (PH51, C=3.2 ng/µL) and β-NADPH (1 mg) over 120 mins as described in the general procedure for HPLC-based assay. Aliquots were taken at the time indicated and analyzed by HPLC. Aliquot was taken at $T = 0$. Aliquot was taken at $T = 40$ mins. DDAO (25 µM). Analytical HPLC (Dionex Acclaim™ 120 C18 column [5 µm, 120 Å, 4.6 × 150 mm]; 95:5 H_2O: MeCN → 5:95 H_2O: MeCN: H_2O with 0.1% TFA modifier, 10 min; 5 min hold; 1.0 mL min^{-1}]. Absorbance was recorded at 254 nm. The injection peak was omitted, and the chromatogram started at 2.5 mins for clarity. (**C**) LRMS spectrum of the aliquot taken from panel **B** showing compound **1** and its corresponding leuco derivative formed after enzymatic reduction under normoxic conditions.

5. Stability and selectivity of the indolequinone-based probes (compounds 1-4)

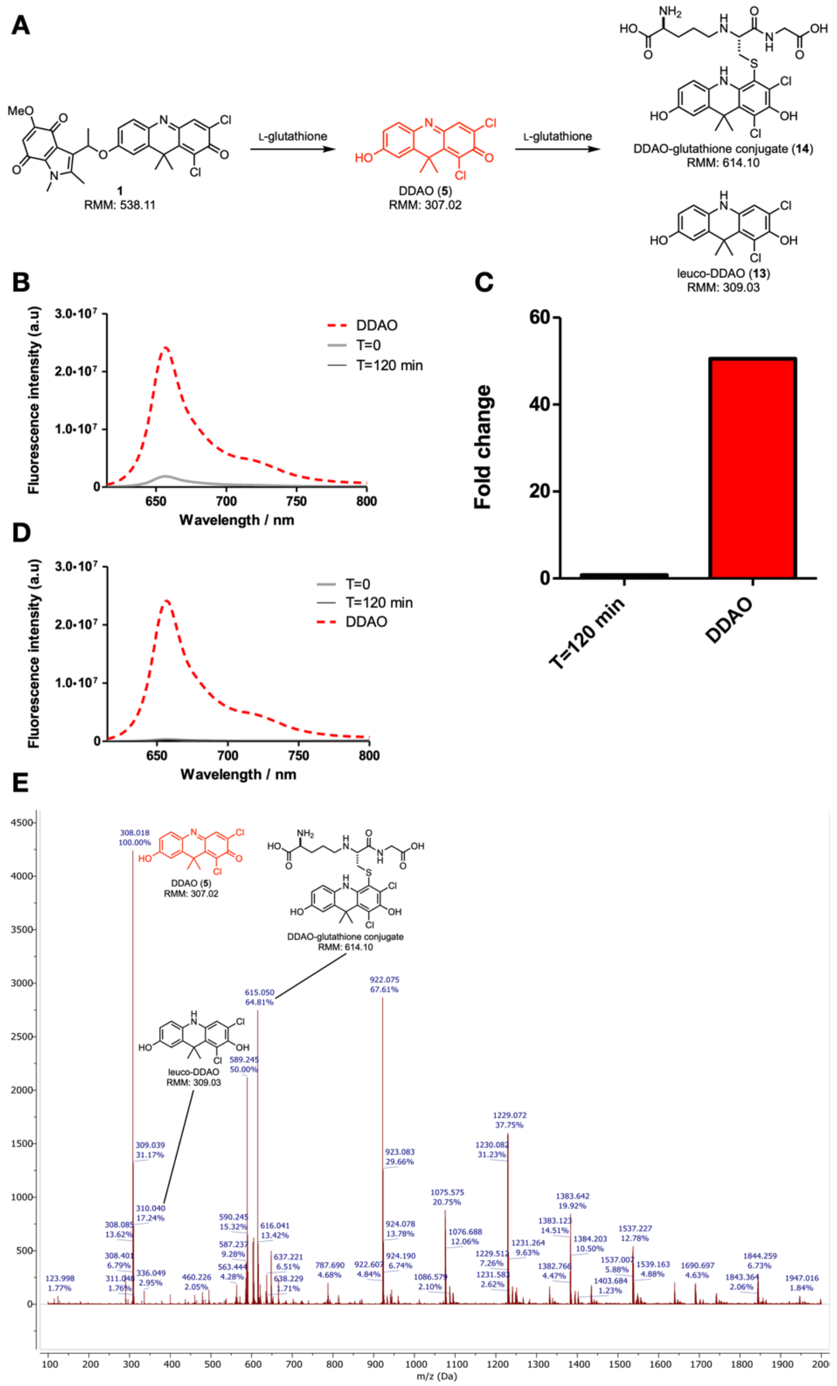

Figure S14. The reaction of compound 1 with L-glutathione (GSH) gives leuco-DDAO (13) and a DDAO-glutathione conjugate (14). (**A**) The proposed reaction of compound **1** with L-glutathione (GSH) to give DDAO (**5**) and then leuco-DDAO (**13**) and the DDAO-GSH conjugate (**14**). (**B**) Compound **1** was treated with GSH (5 mM) in MilliQ water (pH 7) for 120 min, as described in the general procedure. Fluorescence intensity data were collected after the time indicated in the figure with excitation at 600 nm. Slits 3, 3 nm. (**C**) Quantification of the fluorescence increase shown by compound **1**, showing a 1.5% increase in fluorescence after 120 min (where the fluorescence of untreated DDAO is 100%). (**D**) DDAO (**5**) was treated GSH (5 mM) in MilliQ water (pH 7.4) for 120 min, as described in the general procedure, showing only 0.5% of fluorescence remaining (where the fluorescence of untreated DDAO is 100%). Fluorescence intensity data were collected after the time indicated in the figure with excitation at 600 nm. Slits 3, 3 nm. (**E**) LRMS spectrum of the aliquot taken from panel **D**, after 2 h, showing the $[M+H]^+$ peaks for DDAO (**5**), the corresponding leuco-DDAO (**13**), and the glutathione-conjugate (**14**) formed after treatment with GSH in normoxic conditions.

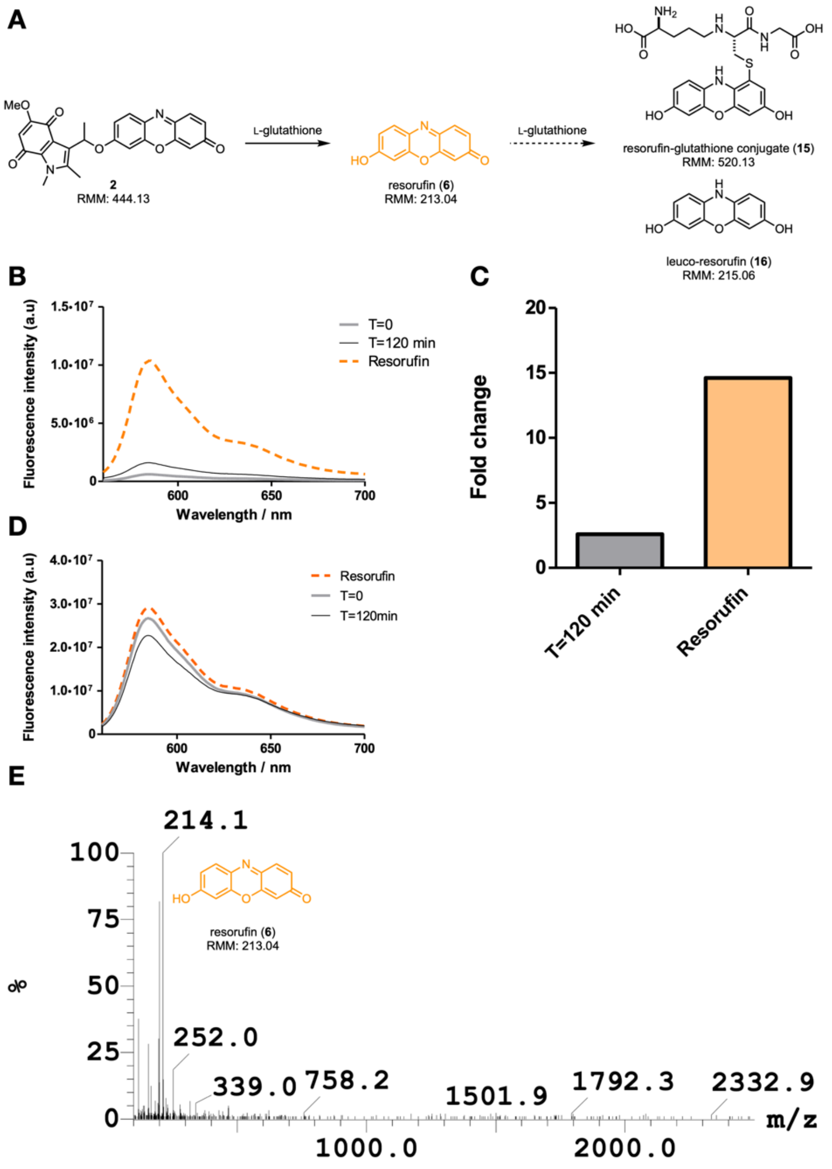

Figure S15. Treatment of probe 2 with L-glutathione (GSH) results in only very modest release resorufin (6). (**A**) The proposed reaction of compound **2** with L-glutathione (GSH) to give modest amounts of (**5**). No evidence of the resorufin-GSH conjugate (**15**) or the leuco-resorufin (**16**) was observed. (**B**) Compound **2** was treated with GSH (5 mM) in MilliQ water (pH 7) for 2 h, as described in the general procedure. Fluorescence intensity data were collected after the time indicated in the figure with excitation at 545 nm. Slits 3, 3 nm. (**C**) Quantification of the fluorescence increase shown by compound **2**, showing an 18% increase in fluorescence after 120 min (where the fluorescence of untreated resorufin is 100%). (**D**) Resorufin (**5**) was treated with GSH (5 mM) in phosphate buffer (pH 7.4) for 2 h, as described in the general procedure, showing 82% of fluorescence remaining (where the fluorescence of untreated resorufin is 100%). Fluorescence intensity data were collected after the time indicated in the figure with excitation at 545 nm. Slits 3, 3 nm. (**E**) LRMS spectrum of the aliquot taken from panel **D** after 2 hours showing resorufin (**6**) after incubation with GSH under normoxic conditions.

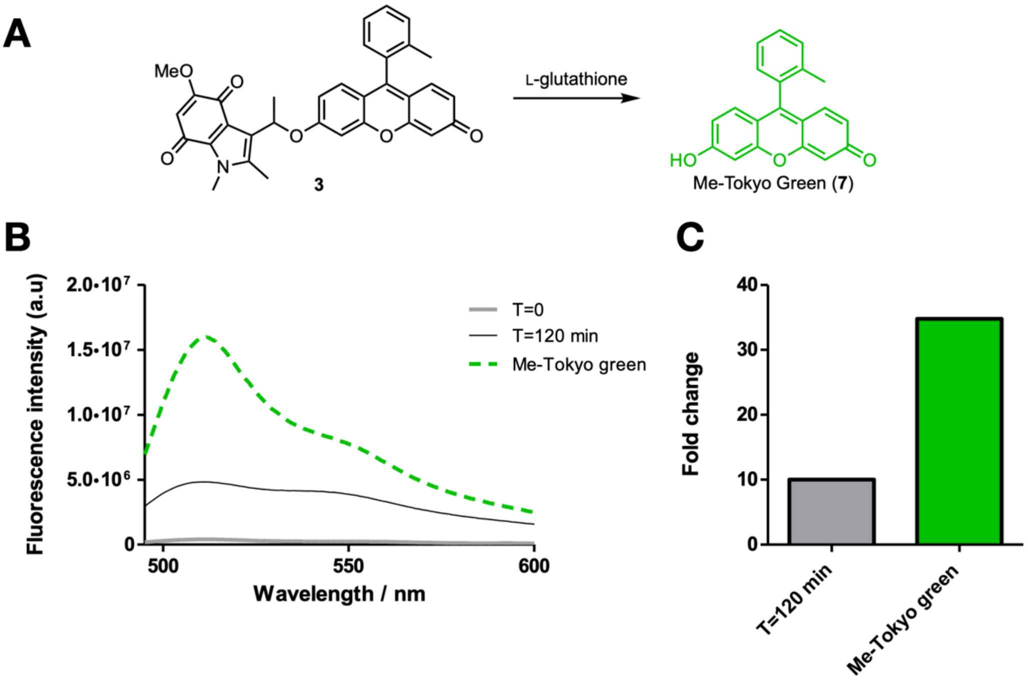

Figure S16. Treatment of probe 3 with GSH results in release of Me-Tokyo Green (7). (**A**) Reduction of compound **3** to **7** using L-glutathione reduced. (**B**) Compound **3** was treated with L-glutathione reduced (5 mM) in MilliQ water (pH 7) for 2 hours as described in the general procedure. Fluorescence intensity data were collected after the time indicated in the figure with excitation at 480 nm. Slits 2, 2 nm. (**C**) Quantification of the fluorescence increase shown by compound **3.**

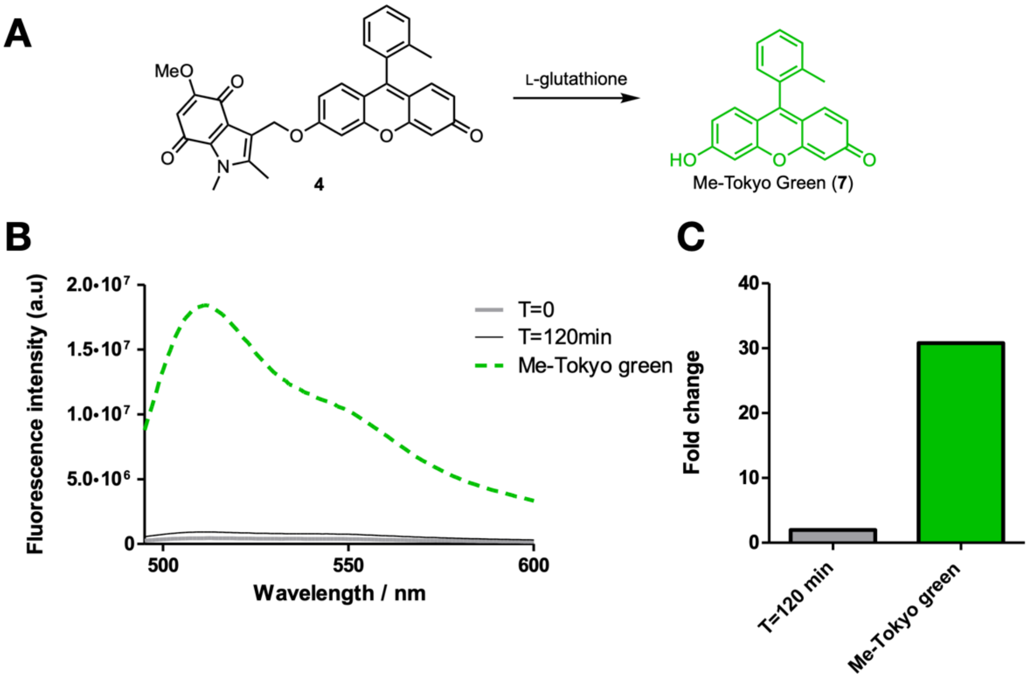

Figure S17. Non-fluorescent compound 4 undergoes glutathione reduction to fluorescent compound 7. (**A**) Reduction of compound **4** to **7** using L-glutathione reduced. (**B**) Compound **4** was treated with L-glutathione reduced (5 mM) in MilliQ water (pH 7) for 2 hours as described in the general procedure. Fluorescence intensity data were collected after the time indicated in the figure with excitation at 480 nm. Slits 2, 2 nm. (**C**) Quantification of the fluorescence increase shown by compound **4.**

6. Biological methods

Cell Lines and Reagents

Colorectal HCT116 (provided by Prof. Bert Vogelstein, Johns Hopkins Medicine) and lung adenocarcinoma A549 cells (ATCC) were grown in DMEM media supplemented with 10% FBS. Cells were maintained in an incubator set at 37 °C and 5% CO_2. All cell lines were routinely tested for mycoplasma using MycoStrip™ (InvivoGen). BioTracker 520 Green Hypoxia Dye (Merck) was re-suspended in 43 µL DMSO and used at a final concentration of 5 µM according to the manufacturer's instructions.

Hypoxic Treatments

Hypoxia treatments at <0.1% O_2 were carried out in a Bactron II anaerobic chamber (Shel Labs) while all oxygen concentrations from 0.5-4% O_2 were carried out in a M35 variable atmosphere workstation (Don Whitley Scientific). Oxygen concentrations were periodically validated using anaerobic oxygen indicator strips (ThermoFisher). Cells were seeded on glass dishes and fixed inside the chamber with equilibrated solutions.

MTT assay

5,000 A549 cells per well were seeded in 96 well plates and allowed to adhere overnight. Cells were treated with the indicated probe and concentration for 6 or 24 hours. Cells were incubated with 0.5 mg/mL MTT reagent (Sigma) in complete media for 3 hours at 37 °C protected from light. MTT was removed and formazan crystals were solubilized with 100 µL of DMSO for 15 minutes at 37 °C protected from light. Absorbance was read at 570 nm (Clariostar, BMG). Data are shown as percentage viability relative to untreated control.

Immunofluorescence

Cells were seeded onto autoclaved cover slips (Menzel-Glaser) before treatment. Cells were fixed inside the chamber with 4% PFA (*w/v* paraformaldehyde in PBS) for 10 minutes. Samples were washed three times with 1 x PBS and mounted onto microscopy slides (Menzel Glaser) with ProLong™ Gold Antifade Mountant with DAPI (Invitrogen™). Cells were visualized with an LSM710 confocal microscope (Carl Zeiss Microscopy Ltd) at 63x magnification and at least 100 cells were counted per condition for quantification.

Flow cytometry

Cells were seeded onto glass 6 cm^2 dishes. Cells were treated with indicated concentration of probe used for 2 hours at the indicated oxygen concentration. Cells were scraped in 1 mL of 1x PBS into an 1.5 mL tube and fixed inside the hypoxia chamber with 4% PFA for 10 minutes. Samples were washed three times with 1 x PBS. Samples were run on a CytoFLEX (Beckman Coulter) and data analyzed using FlowJo software. Filter sets used for probe 2 were PC5.5 (ex 561 nm / em 690/50 nm), for probe 3 and 4 the FITC filter set was used (ex 488 nm / em 525/40 nm)

Spheroid Culture

HCT116 spheroids were formed using the forced aggregation method, 1000-2000 cells were seeded per well into ultra-low-adherent round bottom 96 well plates (Corning) and centrifuged at 2,000 × g. Spheroids were imaged daily using the Celigo Imaging Cytometer (Nexcelom Bioscience).

Spheroid Immunofluorescence Microscopy

Spheroids were fixed in 4% PFA for 2 hours, washed in PBS three times and treated with 30% sucrose PBS [w/v] for 3 hours before mounting in OCT (Fisher Scientific) embedding medium. Spheroids were sectioned as 5 µm slices using a cryostat (Leica), mounted onto slides, and stored at −80 °C. Sections were then rehydrated in 0.1% PBS-Tween [*v/v*] for 10 minutes at room temperature and blocked with 1% bovine serum albumin PBS-Tween (BSA) [*v/v*] for 2 hours at room temperature. Sections were washed with 0.1% PBS-Tween for 10 minutes twice before treatment with Pimonidazole FITC conjugated primary antibody (Hypoxyprobe) for 2 hours at a dilution of 1:100 in 1% BSA PBS [*v/v*]. After this time, sections were washed for 10 minutes twice before mounting using Prolong Gold Antifade medium with DAPI (Invitrogen). Slides were left to dry for 24 hours before imaging with an LSM780 confocal microscope at 63x magnification (Carl Zeiss Microscopy Ltd). For staining with GLUT1, spheroids were re-hydrated and blocked, followed by overnight incubation at 4 °C with GLUT1 antibody (Abcam) at a dilution of 1:250 in 1% BSA PBS [*v/v*]. Spheroids were washed for 10 minutes twice before incubation with goat anti-rabbit Alexa Fluor® 488 or 647 secondary antibody (Abcam) diluted in 1% BSA PBS [*v/v*] for 1 hour at room temperature. Spheroids were washed, mounted, and imaged as described above. Imaging of live intact spheroids was carried out using the EVOS™ M7000 Imaging System (ThermoFisher Scientific).Spheroids were treated with probe for 2 hours. Spheroids were then directly imaged in ultra-low-adherent 96 well plates (Corning). For Tokyo Green derivatives, standard filter sets for FITC were used, for resorufin derivatives standard filter sets for RFP were used. Spheroids were imaged at x5 magnification.

Spheroid Analysis

A custom pipeline was created using CellProfiler based on the method published by Spoerri *et al.*, 2021[1]. Briefly, DAPI staining was used to identify the nuclei as a "primary object", which is then used as a "seed region" together with the probe staining (green or red), to outline the cytoplasmic area and then define the cell (nucleus + cytoplasm). Probe fluorescent intensity signal is then measured for each cell. The DAPI stain was used to create a mask which outlined the whole spheroid. This was used to measure the distance of each cell from the surface of the spheroid. The data obtained was used to generate mean fluorescent intensity at different depths in the spheroid. CellProfiler generated a visual representation of the depth curves overlaid onto the spheroid mask to indicate the distance from the spheroid surface. Bin number 1 indicates the center of the spheroid, bin number 6 indicates the peripheral areas of the spheroid.

[1] *Front. Digit. Health* **2021**, *3*, 668390

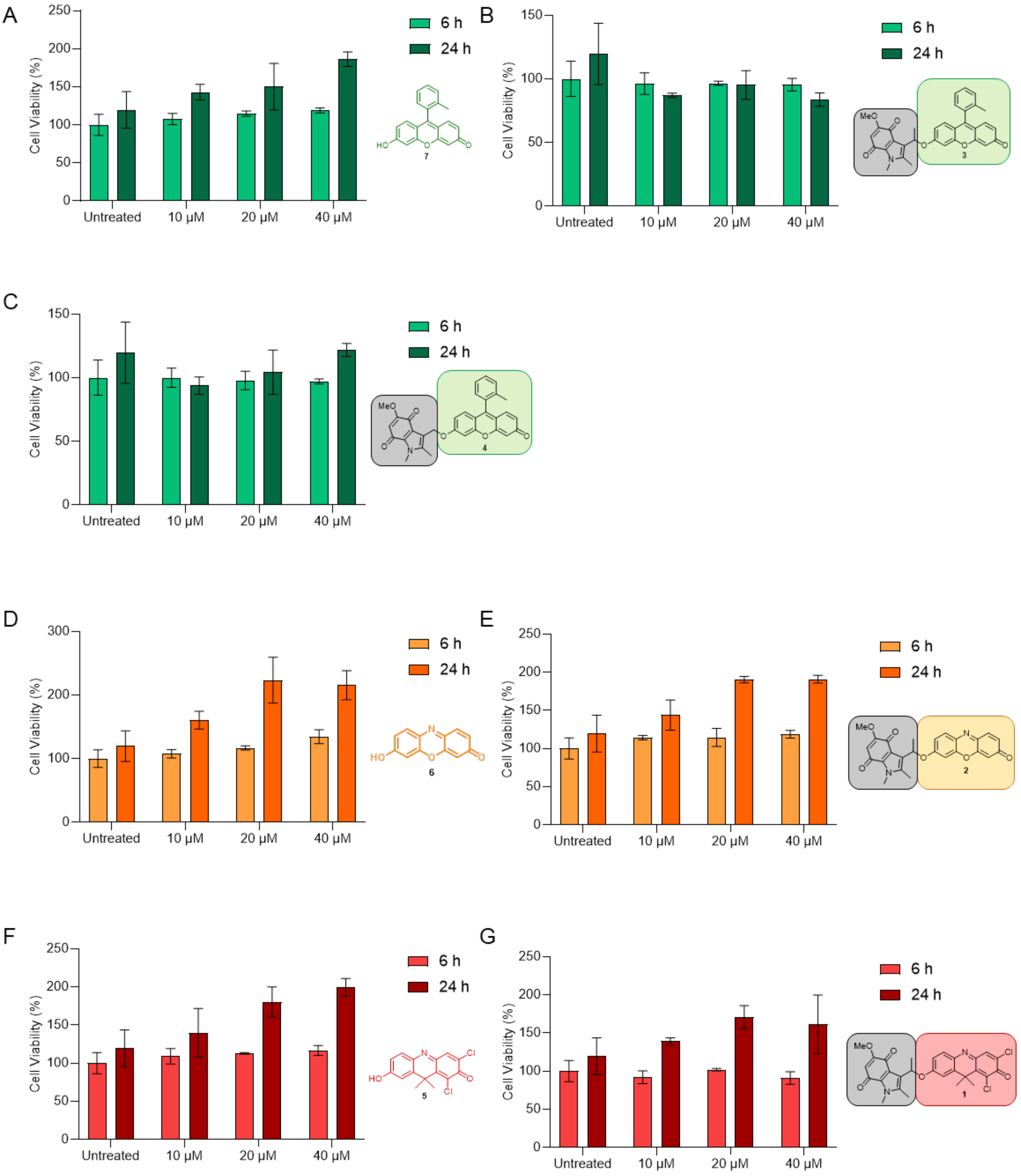

Figure S18. Cellular toxicity of fluorescent reporters and probes. A549 cells were treated with indicated concentration of reporter or probe for 2 hours. Probe was removed and cell viability was assessed 6 hours and 24 hours post treatment using an MTT assay. (**A**) Me-Tokyo Green fluorophore. (**B**) compound **3**. (**C**) compound **4**. (**D**) Resorufin fluorophore. (**E**) compound **2**. (**F**) DDAO fluorophore. (**G**) compound **1**. Data presented as % cell viability relative to untreated control. Error bars represent SD. n=3.

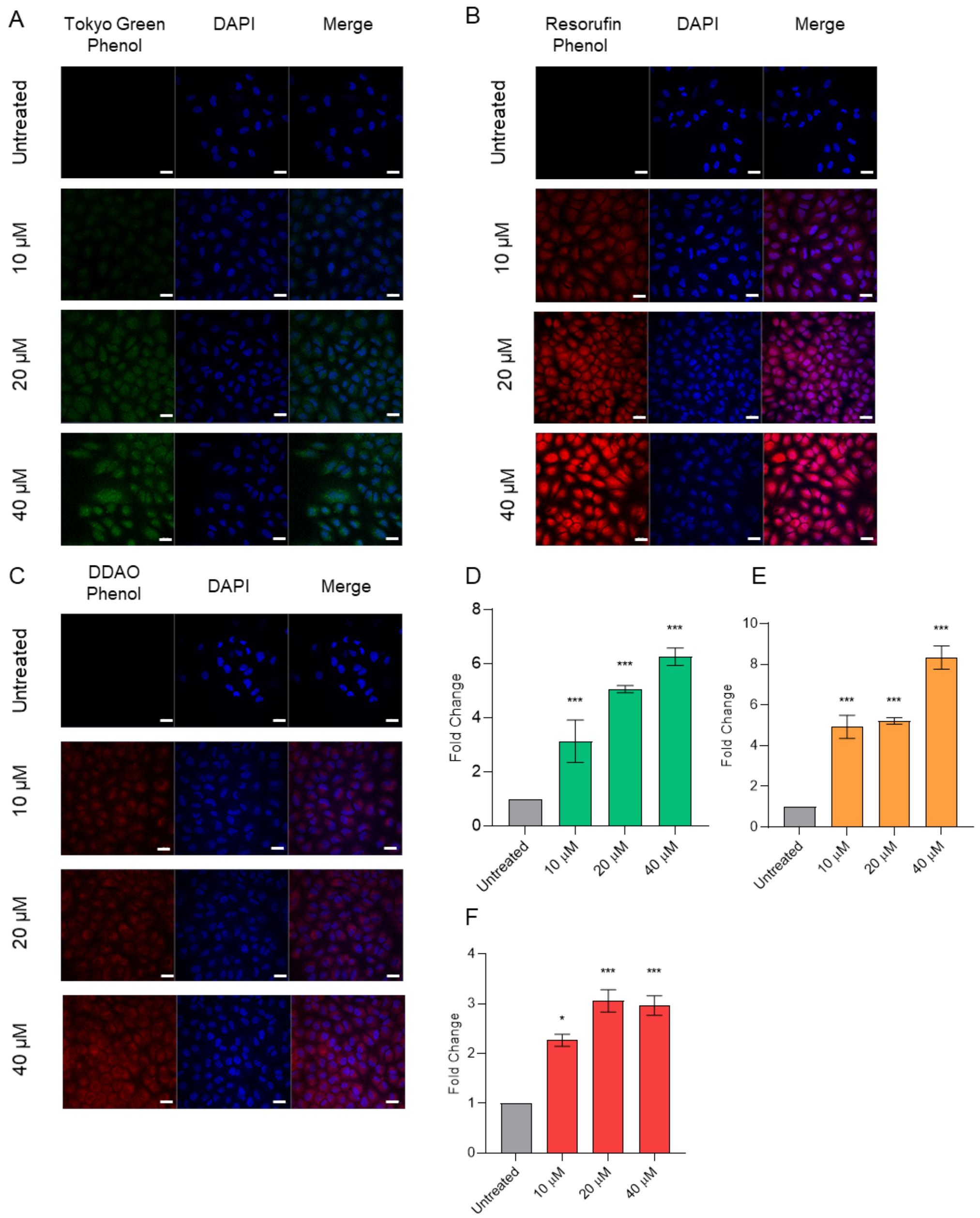

Figure S19. Cell permeability of fluorescent reporters. A549 cells were treated with the indicated concentration of fluorophore for 2 hours. Scale bar = 20 µm. (**A**) Representative images of Me-Tokyo Green. (**B**) Representative images of Resorufin. (**C**) Representative images of DDAO. (**D**) Quantification of microscopy of images in panel **A**, data presented relative to untreated control. (**E**) Quantification of microscopy of images in panel **B**, data presented relative to untreated control. (**F**) Quantification of microscopy of images in panel **C**, data presented relative to untreated control. Error bars represent SD.* $p<0.05$ ** $p<0.01$ and *** $p<0.001$. n=3.

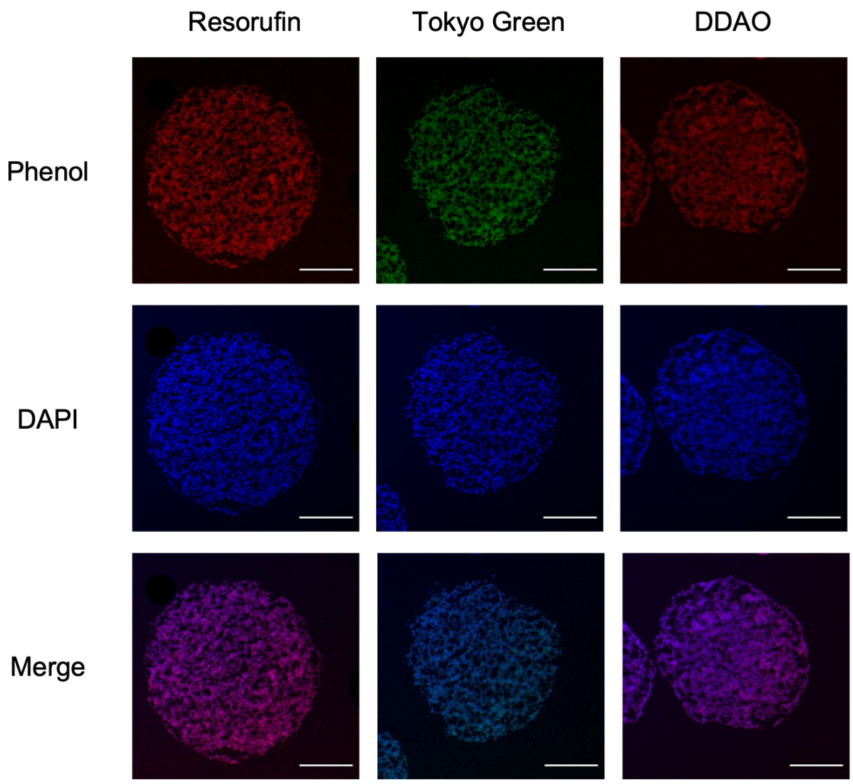

Figure S20. Imaging of phenol controls in spheroids. Representative images of cryosectioned HCT116 spheroids treated with indicated fluorescent dye control for 2 h (20 µM). Scale bar indicates 200 µm. n=7

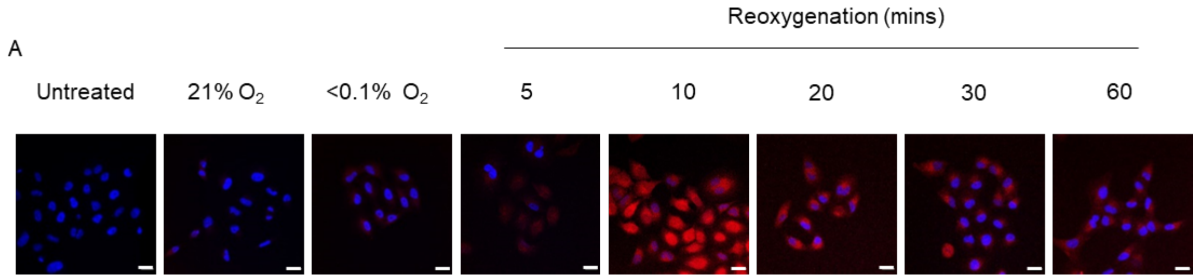

Figure S21. Requirement for reoxygenation of probe **1**. A549 cells were treated with compound **1** (40 µM) for 2 hours and then fixed inside the hypoxia chamber or exposed to 21% O_2. (**A**) Cells were treated with compound **1** and exposed to reoxygenation for the times indicated. Merged images for probe **1** and DAPI are shown. Scale bar = 20 µm. n=1.

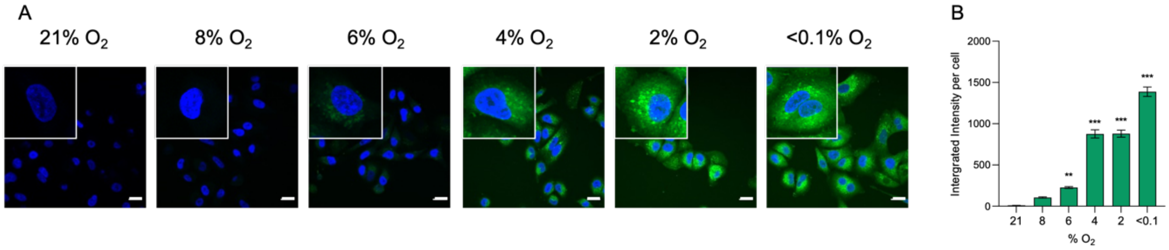

Figure S22 (A). Representative images of cells incubated with BioTracker 520 Green (5 µM). (**B**) Changes in fluorescence intensity of BioTracker 520 plotted as integrated intensity per cell (n=3). Scale bar = 20 µm. Error bars represent SD.* $p<0.05$ ** $p<0.01$ and *** $p<0.001$.

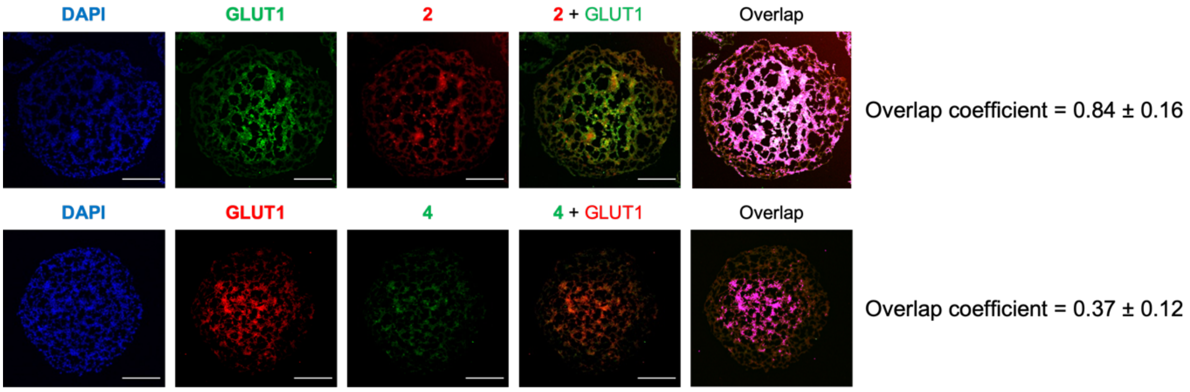

Figure S23. Representative images showing areas of overlap between GLUT1 and the probe indicated, with overlap indicated highlighted in pink and white. (**F**) Probe **2** has a Mander's overlap coefficient value of 0.84 ± 0.16 with GLUT1, while probe **4** has a Mander's overlap coefficient value of 0.34 ± 0.12 with GLUT1 (n=7).

7. Chemistry experimental section

General chemistry experimental

Chemicals were purchased from Sigma Aldrich UK, Alfa Aesar UK, and Fluorochem, and were used as supplied unless stated. Brine refers to a saturated aqueous solution of sodium chloride. Anhydrous solvents were obtained from an MBRAUN Solvent Purification System 5 and stored under an argon atmosphere over 3 Å molecular sieves. Petroleum ether refers to the fraction of light petroleum ether boiling in the range 40–60 °C. *In vacuo* refers to the removal of solvent using a Buchi® rotary evaporator under reduced pressure in a water bath at 40 °C.

^{1}H NMR spectra were recorded on Bruker AVC500 (500 MHz) or Bruker AVH400 (400 MHz) spectrometers using deuterochloroform (unless indicated otherwise) as a reference for the internal deuterium lock. The chemical shift data for each signal are given as δ_H in units of parts per million (ppm) relative to tetramethylsilane (TMS) where δ_H (TMS) = 0.00 ppm. The multiplicity of each signal is indicated by s (singlet); d (doublet); dd (doublet of doublets); or m (multiplet). The number of protons (n) for a given resonance signal is indicated by nH. Coupling constants (*J*) are quoted in Hz and are recorded to the nearest 0.1 Hz. Identical proton coupling constants (*J*) are averaged in each spectrum and reported to the nearest 0.1 Hz. The coupling constants are determined by analysis using MestreNova software. Bruker Topspin was used to plot the spectra. Spectra were assigned using COSY, NOESY, HSQC, and HMBC experiments as necessary.

^{13}C NMR spectra were recorded on a Bruker AVC500 (126 MHz) or AVH400 (100 MHz) spectrometers in the stated solvents, with broadband proton decoupling and an internal deuterium lock. The chemical shift data for each signal are given as δ_C in units of parts per million (ppm) relative to tetramethylsilane (TMS) where δ_C (TMS) = 0.00 ppm. The shift values of resonances are quoted to 1 decimal place and were determined using Bruker MestreNova software. Bruker Topspin was used to plot the spectra. Spectra were assigned using HSQC, HMBC and NOESY experiments as necessary.

When two isomers are present in the sample, A and B denote each of the two isomers without distinguishing between them. A is arbitrarily assigned to the isomer with the highest ppm shift and B to the isomer with the lowest ppm shift, in ^{1}H NMR and ^{13}C NMR.

Mass spectra were acquired on a VG platform spectrometer and an Agilent 6120 spectrometer (low resolution). Electro-spray ionization spectra were obtained on Micromass LCT Premier and Bruker MicroTOF spectrometers, operating in a positive or negative mode, as indicated, from solutions of MeOH or MeCN. *m/z* values are reported in Daltons and followed by their percentage abundance in parentheses. Electron ionization/field ionization (EI/FI) was carried out on a Waters GCT with a temperature-programmed solids probe inlet. Samples were introduced in glass tips directly into the source where they were vaporized and analyzed. In EI the ionization is by electron impact, electrons being provided by a filament. In FI the ionization is in an intense electric field which causes quantum electron tunneling of a valence electron.

Melting points were determined using a Griffin capillary tube melting point apparatus and are uncorrected.

Infrared spectra were obtained from neat samples, either as solids or liquids, using a diamond ATR module. The spectra were recorded on a Bruker Tensor 27 spectrometer. Absorption maxima are recorded in wavenumbers (cm^{-1}) and reported as s (strong), m (medium), w (weak), or br (broad).

Analytical thin-layer chromatography (TLC) was carried out on normal phase Merck silica gel 60 F254 aluminum-supported chromatography sheets. Visualization was done by absorption of UV light (λ_{max} 254 and 365 nm) or thermal development after staining in an aqueous solution of potassium permanganate. UV light was provided by a LF – 206.LS 230V – 50 Hz from UVltec Limited

Flash column chromatography was performed manually using Geduran® silica gel 60 (40-63 µm) eluting with solvents as supplied, under a positive pressure of air or nitrogen on a Biotage® Selekt Flash Pure Purification System using Biotage® KP-Sil SNAP or Biotage® Sfär Silica cartridges.

Fluorescence spectroscopy: HORIBA Jobin Yvon FluoroLog3 fluorimeter (Hamamatsu R928 detector and a double-grating emission monochromator) was used to acquire the luminescence spectra. The standard conditions for acquiring emission and excitation spectra are room temperature and steady-stated mode unless otherwise stated. HORIBA Jobin Yvon FluoroLog3 fluorimeter system equipped with a Xenon flash lamp was used to acquire emission lifetimes. Fluorescence spectra were obtained by using GraphPad Prism 8 software (GraphPad Software Inc.).

UV-visible spectra were recorded on a V-770 UV-Visible/NIR Spectrophotometer equipped with Peltier temperature controller and stirrer using disposable polystyrene cuvettes of 1 cm path length. Experiments were conducted at 25 °C unless otherwise stated. UV-visible spectra were plotted using GraphPad software.

Synthetic procedures

1-(5-Methoxy-2-methyl-1*H*-indol-3-yl)ethan-1’-one (S1)

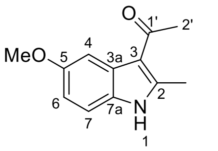

A solution of 5-methoxy-2-methylindole (2.50 g, 15.5 mmol, 1.0 eq.) in anhydrous diethyl ether (20.0 mL) was added dropwise, with vigorous stirring, to a solution of ethyl magnesium bromide (10.0 mL, 30.2 mmol, 1.9 eq.) in anhydrous diethyl ether (12.5 mL) under an argon atmosphere. The solution was heated under reflux for 1 hour, cooled to 0 °C and a solution of acetyl chloride (6.62 mL, 93.0 mmol, 6.0 eq.) in anhydrous diethyl ether (10.0 mL) added with vigorous stirring. The resulting suspension was heated under reflux for a further 1 hour, cooled to room temperature, and saturated aqueous ammonium chloride (150 mL) was added. The solution was extracted with ethyl acetate (4 × 100 mL), and the combined organic components were washed with saturated aqueous sodium bicarbonate solution (200 mL) and brine (200 mL), then approximately 75% of the solvent was removed by evaporation. The resulting precipitate was collected and washed with diethyl ether to give the title compound (**S1**) as an off-white solid (3.0 g, 95%): R_f 0.23 (petroleum ether:ethyl acetate, 1:1) m.p. 216–221 °C (from diethyl ether) [lit.[2] 219–224 °C]; ^1H NMR (400 MHz, DMSO) δ 7.55 (d, *J* = 2.5, 1H, *H-4*), 7.25 (d, *J* = 8.7, 1H, *H-7*), 6.76 (dd, *J* = 8.7, 2.5 , 1H, *H-6*), 3.77 (s, 3H, OCH_3), 2.65 (s, 3H, *H-2’*), 2.48 (s, 3H, (C-2)CH_3; LRMS *m/z* (ESI^+) 204 ($[M+H]^+$, 100%). The spectroscopic data are in good agreement with literature values.[2]

[2] *J. Chem. Soc., Perkin Trans. 2*, **2001**, 843-860

1-(5-Methoxy-1,2-dimethyl-1*H*-indol-3-yl)ethan-1'-one (S2)

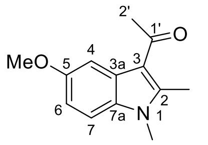

Compound **S1** (1.75 g, 8.61 mmol, 1.0 eq.) was added to a stirred suspension of sodium hydride (730 mg, 60% *w/w* dispersion in oil, 18.3 mmol, 2.1 eq.) in anhydrous tetrahydrofuran (35 mL). The solution was stirred at 50 °C for 30 minutes, cooled to room temperature and iodomethane (4.6 mL, 73.6 mmol, 8.5 eq.) was added slowly. The solution was heated under reflux for 1 hour, cooled to room temperature, and added to a cold solution of sodium thiosulfate (10%, 50 mL). The solution was extracted with ethyl acetate (2 × 150 mL). The combined organic components were washed with saturated sodium bicarbonate solution (100 mL), dried (sodium sulfate), filtered, and concentrated *in vacuo*. The crude material was purified using silica gel column chromatography eluting with ethyl acetate:hexane (1:1) to give the title compound as light yellow solid (1.65 g, 89%): R_f 0.35 (ethyl acetate:hexane, 1:1); m.p. 94–96 °C; ^{1}H NMR (400 MHz, $CDCl_3$) δ 7.52 (d, *J* = 2.4 Hz, 1H, *H-4*), 7.20 (dd, *J* = 8.9, 0.5, 1H, *H-7*), 6.89 (dd, *J* = 8.9, 2.4, 1H, *H-6'*), 3.89 (s, 3H, OCH_3), 3.66 (s, 3H, NCH_3), 2.73 (s, 3H, *H-2'*), 2.63 (s, 3H, (C-2)CH_3); LRMS *m/z* (ESI^+) 218 ($[M+H]^+$, 37.68%), 235 ($[2M+H]^+$, 3.96%). The spectroscopic data are in good agreement with literature values.[2]

3-Acetyl-5-methoxy-1,2-dimethyl-1*H*-indole-4,7-dione (S3)

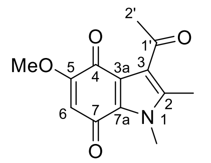

Step 1: Compound **S2** (1.00 g, 4.92 mmol, 1.0 eq.) was dissolved in glacial acetic acid (16 mL) and cooled to 0–4 °C. Fuming nitric acid (3.69 mL) in acetic acid (10.61 mL) was then added slowly with stirring at 0–4 °C, for over 1 hour. The solution was then stirred at 0–4 °C for a further 1 hour. Crushed ice (107 g) was then added to the solution and the resulting suspension was stirred for 30 minutes. The resulting pale orange solid was filtered, washed with water, and dried *in vacuo* for 12 hours to give 1-(5-methoxy-1,2-dimethyl-4-nitro-1*H*-indol-3-yl)ethan-1'-one (1.08 g) as a yellow solid which was used without further purification.

Step 2: 1-(5-Methoxy-1,2-dimethyl-4-nitro-1*H*-indol-3-yl)ethan-1'-one (1.08 g, 4.12 mmol, 1.0 eq.) (above), was dissolved in ethanol (75 mL) and powdered tin (2.57 g, 21.6 mmol, 5.2 eq.) was added, followed by hydrochloric acid (9.0 M, 32 mL). The solution was heated under reflux (80 °C) for 1 hour. After this time water (250 mL) was added, and the pH was adjusted to 8 with a saturated aqueous solution of sodium hydrogen carbonate. The aqueous phase was extracted with chloroform (4 × 300 mL), the combined organic components were washed with brine (250 mL), dried (sodium sulfate), filtered, and concentrated *in vacuo* to yield the desired compound as a brown solid 960 mg which was used without further purification.

Step 3: To a solution of 1-(4-amino-5-methoxy-1,2-dimethyl-1*H*-indol-3-yl)ethan-1'-one (912 mg, 3.92 mmol, 1.0 eq.) in acetone (300 mL) was added a solution of Fremy's salt (5.26 g, 19.6 mmol, 5.0 eq.) in aqueous NaH_2PO_4/Na_2HPO_4 buffer (300 mL, 0.4 M, pH 6.0) and the solution was stirred at room temperature for 1 hour. After this time acetone was removed *in vacuo* and the aqueous phase was extracted with ethyl acetate (4 × 300 mL). The combined organic components were dried (sodium sulfate), filtered, and concentrated *in vacuo*. The crude material was purified using silica gel column chromatography eluting with dichloromethane:acetone (9:1) to give the title compound as a yellow/orange solid (464 mg, 48% over three steps): R_f 0.9 (dichloromethane:acetone, 9:1); m.p. 212–214 °C (acetone) [lit.[2] 227–228 °C]. ^{1}H NMR (400 MHz, $CDCl_3$) δ 5.68 (s, 1H, *H-6*), 3.92 (s, 3H, OCH_3), 3.84 (s, 3H, NCH_3), 2.64 (s, 3H, *H-2'*), 2.38 (s, 1H, (C-2)CH_3); LRMS *m/z*

(ESI$^+$) 248 ([M+H]$^+$, 2.87%), 521 ([2M+Na]$^+$, 86.65%). The spectroscopic data are in good agreement with literature values.[2]

3-(1'-Hydroxyethyl)-5-methoxy-1,2-dimethyl-1*H*-indole-4,7-dione (8)

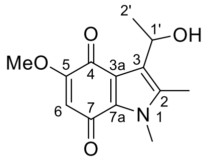

To a suspension of compound **S3** (280 mg, 1.13 mmol, 1.0 eq.) at 0 °C in anhydrous methanol (115 mL) under argon, was added sodium borohydride (1.11 g, 29.4 mmol, 26 eq.). The solution was stirred for 45 min, before water (50 mL) and ethyl acetate (50 mL) were added. The suspension was stirred vigorously, then extracted with ethyl acetate (4 × 100 mL). The combined organic components were washed with saturated sodium bicarbonate solution (100 mL), brine (100 mL), dried (sodium sulfate), filtered, and concentrated *in vacuo*. The crude material was purified using silica gel column chromatography eluting with ethyl acetate:hexane (2:1) to give the title compound as a red solid (262 mg, 93%): R_f 0.75 (ethyl acetate), m.p. 152–154 °C (from ethyl acetate) [lit.[1] 148–150 °C]; ^{1}H NMR (400 MHz, $CDCl_3$) δ 5.60 (d, *J* = 2.0, 1H, *H-6*), 4.79 (q, *J* = 5.7 , 1H, *H-1'*), 3.84 (d, *J* = 2.0, 3H, OCH_3), 3.80 (s, 3H, NCH_3), 2.18 (s, 3H, (C-2)CH_3), 1.40 (d, *J* = 5.7, 3H, *H-2'*); LRMS *m/z* (ESI$^+$) 250 ([M+H]$^+$, 3.71%); 521 ([2M+Na]$^+$, 86.65%). The spectroscopic data are in good agreement with literature values.[2]

5-Methoxy-2-methyl-1*H*-indole-3-carbaldehyde (S4)

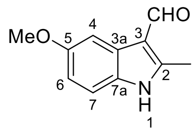

Phosphorus(V) oxychloride (1.74 mL, 18.4 mmol, 1.4 eq.) was added to anhydrous *N*,*N*-dimethylformamide (6.02 mL, 77.8 mmol, 6.0 eq.) at 0 °C while stirring. After addition, the mixture was stirred at 0 °C for 10 minutes to form the presumed Vilsmeier's reagent. 5-Methoxy-2-methylindole (2.08 g, 12.9 mmol, 1.0 eq.) was dissolved in anhydrous *N*,*N*-dimethylformamide (6.02 mL), cooled to –10 °C, the presumed Vilsmeier's reagent was added dropwise over 30 minutes, and the resulting solution was stirred for 30 minutes. The reaction mixture was warmed to 0 °C and stirred further for 15 minutes. The mixture was added to a cooled (0 °C) 2 M sodium hydroxide solution (100 mL) and dichloromethane (200 mL) was added. The aqueous layer was extracted with dichloromethane (2 × 100 mL), the combined organic components were washed with brine (100 mL), 0.5 M aqueous lithium chloride solution (2 × 50 mL), dried (sodium sulfate), filtered, and concentrated *in vacuo*. The crude material was washed with ethyl acetate (3 × 50 mL) at 0 °C to give the title compound as a pale brown solid (1.73 g, 71%): R_f 0.26 (hexane:ethyl acetate, 1:1); m.p. 180–183 °C (from ethyl acetate) [lit.[3] 198–199 °C, lit.[4] 191–194 °C, lit.[5] 120–122 °C]; ^{1}H NMR (500 MHz, $CDCl_3$) δ 10.15 (1H, s, C*HO*), 8.55 (1H, s, N*H*), 7.77 (1H, d, *J* = 2.5, *H-4*), 7.21 (1H, d, *J* = 8.8 Hz, *H-7*), 6.87 (1H, dd, *J* = 8.8, 2.5, *H-6*), 3.87 (3H, s, OCH_3), 2.72 (s, 3H, (C-2)CH_3); LRMS *m/z* (ESI$^+$) 190 ([M+H]$^+$ 60%), 162 ([M−CHO]$^+$, 70%). The spectroscopic data are in good agreement with the literature values.[3-5]

5-Methoxy-1,2-dimethyl-1*H*-indole-3-carbaldehyde (S5)

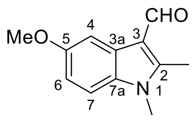

A solution of compound **S4** (900 mg, 4.76 mmol, 1.0 eq.) in anhydrous *N*,*N*-dimethylformamide (6.00 mL) was added gradually to a stirred suspension of sodium hydride (290 mg, 60% *w/w* in mineral oil, 7.15 mmol, 1.5 eq.) in anhydrous *N*,*N*-dimethylformamide

[3] *Org. Lett.* **2010**, *12*, 1384-1387
[4] *Eur. J. Med. Chem.* **2005**, *40*, 505-519
[5] *Bioorg. Med. Chem. Lett.* **2009**, *19*, 5016-5020

(5.50 mL) under an argon atmosphere. The mixture was stirred at room temperature for 2 hours and cooled to 0 °C. Iodomethane (0.360 mL, 5.75 mmol, 1.2 eq.) was added dropwise, and the reaction mixture was warmed to room temperature and stirred for an additional 2 hours. After this time water (50 mL) was added slowly to the mixture and the aqueous layer was extracted with dichloromethane (3 × 40 mL). The combined organic components were washed with water (50 mL), 0.5 M aqueous lithium chloride solution (50 mL), brine (50 mL), dried (sodium sulfate) filtered, and concentrated *in vacuo*. The crude material was purified using silica gel column chromatography, eluting with hexane:ethyl acetate (gradient 20 to 100% ethyl acetate) to give the title compound as a light yellow solid (780 mg, 81%): R_f 0.23 (hexane:ethyl acetate, 1:1); m.p. 115–118 °C (from ethyl acetate) [lit.[6] 132–133 °C]; ^{1}H NMR (500 MHz, $CDCl_3$) δ 10.11 (s, 1H, C*H*O), 7.79 (d, *J* = 2.6, 1H, *H-4*), 7.18 (d, *J* = 8.9, 1H, *H-7*), 6.90 (dd, *J* = 8.9, 2.6, 1H, *H-6*), 3.89 (s, 3H, OC*H₃*), 3.66 (s, 3H, NC*H₃*), 2.65 (s, 3H, (C-2)C*H₃*); LRMS *m/z* (ESI^+) 204 ($[M+H]^+$, 81%), 176 ($[M-CHO]^+$, 100%). The spectroscopic data are in good agreement with the literature values.[6]

4-Amino-5-methoxy-1,2-dimethyl-1*H*-indole-3-carbaldehyde (S6)

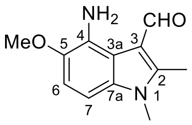

Step 1: Compound **S5** (350 mg, 1.67 mmol, 1.0 eq.) in acetic acid (40 mL) was cooled to 0 °C. A mixture of fuming nitric acid (1.57 mL, 37.6 mmol, 22.5 eq.) in acetic acid (6.31 mL) was added dropwise with stirring over the period of 1 hour. The reaction mixture was stirred further for 3 hours, when the mixture was added to crushed ice water (72 g). The suspension was stirred for 1 hour and the yellow precipitate was collected using suction filtration, washed with water (200 mL), and dried *in vacuo* to afford the desired 5-methoxy-1,2-dimethyl-4-nitro-1*H*-indole-3-carbaldehyde (400 mg) as a yellow solid which was used without further purification.

Step 2: To a solution of 5-methoxy-1,2-dimethyl-4-nitro-1*H*-indole-3-carbaldehyde (400 mg, 1.61 mmol, 1.00 eq.) in ethanol (30.0 mL), was added powdered tin (1.00 g, 8.46 mmol, 5.25 eq.), followed by aqueous hydrochloric acid solution (3.0 M, 12.4 mL, 37.30 mmol, 23.15 eq.). The reaction mixture was heated at 80 °C for 1 hour. Water (100 mL) was added, and the pH adjusted to 8 with solid sodium hydrogen carbonate. The aqueous layer was extracted with chloroform (3 × 100 mL) and the combined organic components were washed with brine (50 mL), dried (sodium sulfate), and concentrated *in vacuo* to yield the title compound as a brown-yellow solid (350 mg, 50%): R_f 0.57 (ethyl acetate); m.p. 152–153 °C (from ethyl acetate); ^{1}H NMR (400 MHz, $CDCl_3$) δ 9.80 (s, 1H, C*H*O), 6.86 (d, *J* = 8.6, 1H, *H-7*), 6.47 (d, *J* = 8.6, 1H, *H-6*), 5.92 (s, 2H, N*H₂*), 3.87 (s, 3H, OC*H₃*), 3.59 (s, 3H, NC*H₃*), 2.60 (s, 3H, (C-2)C*H₃*); LRMS *m/z* (ESI^+) 219 ($[M+H]^+$, 100%). The spectroscopic data are in good agreement with the literature values.[6]

5-Methoxy-1,2-dimethyl-4,7-dioxo-4,7-dihydro-1*H*-indole-3-carbaldehyde (S7)

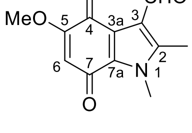

To a solution of compound **S6** (345 mg, 1.58 mmol, 1.0 eq.) in acetone (100 mL), was added a solution of Fremy's salt (2.12 g, 7.90 mmol, 5.0 eq.) in aqueous NaH_2PO_4/Na_2HPO_4 buffer (100 mL, 0.4 M, pH 6.0) and the resulting solution was stirred at room temperature for 1 hour. After this time the acetone was removed *in vacuo* and the aqueous phase was extracted with ethyl acetate (4 × 75 mL). The combined organic components were dried (sodium sulfate), and concentrated *in*

[6] *J. Med. Chem.* **1997**, 40, 2335-2346

vacuo. The crude material was purified using silica gel column chromatography, eluting with dichloromethane:acetone (gradient 2.5 to 10% acetone) to give the title compound as an orange solid (310 mg, 84%): R*f* 0.4 (acetone:dichloromethane 1:2); m.p. 217–219 °C (from acetone) [lit.[5] 239–242 °C]; ^{1}H NMR (400 MHz, $CDCl_3$) δ 10.52 (s, 1H, C*H*O), 5.68 (s, 1H, *H-6*), 3.92 (s, 3H, OC*H₃*), 3.83 (s, 3H, NC*H₃*), 2.60 (s, 3H, (C-2)C*H₃*); LRMS *m/z* (ESI$^+$) 234 ([M+H]$^+$, 100%). The spectroscopic data are in good agreement with the literature values.[6]

3-(Hydroxymethyl)-5-methoxy-1,2-dimethyl-1H-indole-4,7-dione (9)

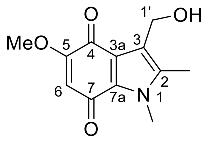

Sodium borohydride (231 mg, 6.10 mmol, 5.0 eq.) was added portion-wise to a suspension of compound **S7** (284 mg, 1.20 mmol, 1.0 eq.) in anhydrous methanol (20 mL) and tetrahydrofuran (20 mL) under argon and cooled to 0 °C, while a dry argon atmosphere was maintained. The solution was stirred for 45 minutes at 0 °C before water (20 mL) was added slowly. The resulting solution was extracted with dichloromethane (3 × 50 mL), dried (sodium sulfate), filtered, and concentrated *in vacuo*. The crude material was purified using silica gel column chromatography, eluting with hexane:ethyl acetate (gradient 50 to 100% ethyl acetate) to give the title compound as a red solid (176 mg, 62%): R*f* 0.45 with ethyl acetate; m.p. 195–198 °C (from ethyl acetate) [lit.[7] 199–200 °C, lit.[6] 215–216 °C]; ^{1}H NMR (400 MHz, $CDCl_3$) δ 5.61 (s, 1H, *H-6*), 4.60 (d, *J* = 7.0, 2H, *H-1'*), 3.86 (s, 3H, OC*H₃*), 3.81 (s, 3H, NC*H₃*), 2.22 (s, 3H, (C-2)C*H₃*); LRMS *m/z* (ESI$^+$) 236.0 ([M+H]$^+$, 100%). The spectroscopic data are in good agreement with the literature values.[6,7]

3-(Chloromethyl)-5-methoxy-1,2-dimethyl-1*H*-indole-4,7-dione (S8)

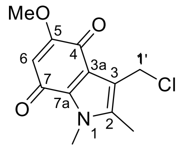

Thionyl chloride (2.10 mL, 28.9 mmol, 34 eq.) was added to a solution of compound **6** (200 mg, 0.850 mmol, 1 eq.) in dichloromethane (1.5 mL) and stirred for 1 hour at room temperature. After this time, concentration *in vacuo* and crystallization from ethyl acetate (10 mL) gave the titled compound as an orange solid (133 mg, 62% yield): m.p. 201–203 °C (from ethyl acetate) [lit.[8] 202–204 °C]; ^{1}H NMR (400 MHz, $CDCl_3$) δ 5.61 (s, 1H, *H-6*), 4.86 (s, 2H, *H-1'*), 3.89 (s, 3H, OC*H₃*), 3.80 (s, 3H, NC*H₃*), 2.28 (s, 3H, (C-2')C*H₃*);); LRMS *m/z* (ESI$^+$ not detected). The spectroscopic data are in good agreement with literature values.[8]

[7] *J. Med. Chem.* **1994**, *37*, 3834-3843
[8] *J. Med. Chem.* **1998**, 41, 2720-2731

3-(2'-Hydroxypropan-2'-yl)phenol (S9)

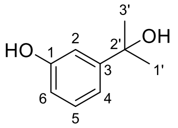

Methyl magnesium chloride (40.0 mL, 119 mmol, 2.2 eq., 3 M in diethyl ether) was added dropwise to a solution of 1-(3-hydroxyphenyl)ethanone (7.4 g, 54.0 mmol, 1.0 eq.) in anhydrous tetrahydrofuran (300 mL) at 0 °C under an argon atmosphere. The reaction mixture was stirred at room temperature for 18 hours and then heated under reflux for 2 hours. After this time the reaction mixture was cooled to 0 °C and saturated aqueous ammonium chloride (50 mL) was added, followed by aqueous hydrochloric acid solution (120 mL, 1 M solution). The aqueous phase was extracted with ethyl acetate (2 × 150 mL). The combined organic components were washed with brine (2 × 200 mL), dried (sodium sulfate), filtered, and concentrated *in vacuo*. The crude material was purified using silica gel column chromatography eluting with ethyl acetate:petrol ether (gradient 10% to 25% ethyl acetate) to give the title compound as a pale-yellow solid (3.6 g, 44%): R_f 0.35 (petroleum ether/ethyl acetate, 1:1); m.p. 99–101 °C (from ethyl acetate) [lit.[9] 101–103 °C]. ^{1}H NMR (400 MHz, $CDCl_3$) δ 7.21 (dd, J = 7.8, 0.6 Hz, 1H, *H-4*), 7.06–6.98 (m, 2H, *H-2* and *H-6*), 6.72 (ddd, J = 7.8, 2.5, 0.9 Hz, 1H, *H-5*), 5.09 (s, 1H, O*H*), 1.57 (s, 6H, *H-3'* and *H-1'*); LRMS *m/z* (ESI$^+$ not detected). The spectroscopic data are in good agreement with literature values.[9]

1,3-Dichloro-7-hydroxy-9,9-dimethylacridin-2(9*H*)-one (DDAO, 5)

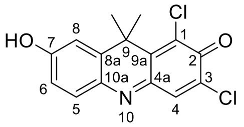

Aqueous sodium hydroxide (21.0 mL, 42.0 mmol, 2.1 eq. 2 M solution) was added dropwise to a solution of compound **S9** (3.04 g, 20.0 mmol, 1.0 eq.) and 2,6-dichloroquinone-4-chloroimide (4.21 g, 20.0 mmol, 1.0 eq.) in tetrahydrofuran (10 mL) and water (10 mL) at 0 °C. The mixture was stirred for 2 hours, then, saturated aqueous ammonium chloride solution (700 mL) was added to the reaction mixture. The aqueous phase was extracted with ethyl acetate (4 × 200 mL). The combined organic components were washed with 10% *w/v* aqueous sodium dithionite solution (2 × 200 mL) and brine (2 × 200 mL). After evaporation of ethyl acetate, the residue was dissolved in methanol (40 mL) and mixed into deoxygenated aqueous hydrochloric acid (500 mL, 2 M solution). The resulting suspension was heated under reflux, under an argon atmosphere for 1.5 hours, during which time a gummy solid formed. The mixture was cooled to room temperature, extracted with ethyl acetate (2 × 400 mL), and the combined organic components were washed with brine (100 mL). A solution of sodium periodate (5.99 g, 28.0 mmol, 1.4 eq.) in water (200 mL) was added to the combined organic components and the resulting solution was stirred vigorously for 16 hours. After this time the aqueous and organic components were separated, and the organic component was washed with brine (200 mL), dried (sodium sulfate), filtered, and concentrated *in vacuo*. The residue was taken up in hot ethanol (800 mL) and concentrated to *ca.* 150 mL by boiling. Upon cooling, the product was filtered by suction and the black solid was washed with cold ethanol (3 × 50 mL). The solid was dried under vacuum to give the title compound as black needles (5.3 g, 86%): R_f 0.5 (dichloromethane/methanol, 95:5); m.p. >290 °C (from ethanol) [lit.[9] >250 °C];^{1}H NMR (400 MHz, D_6-DMSO) δ 7.78 (s, 1H, *H-4*), 7.51 (d, J = 8.6 Hz, 1H, *H-5*), 7.06 (d, J = 2.3 Hz, 1H, *H-8*), 6.85 (dd, J = 8.7, 2.2 Hz, 1H, *H-6*), 1.79 (s, 6H, 2 × C*H*$_3$); LRMS: *m/z* (ESI$^+$) 308.751 ([M+H]$^+$, 26.71%). Analytical HPLC (Dionex Acclaim™ 120 C18 column [5 µm, 120 Å, 4.6 × 150 mm]; 95:5 H_2O: MeCN → 5:95 H_2O: MeCN: H_2O with 0.1% TFA modifier, 10 min; 5 min hold;

[9] *J. Org. Chem.* **1954,** *19*, 1067-1079

1.5 mL min^{-1}] Ret. Time = 10.5 min. Purity: 100%. The spectroscopic data are in good agreement with literature values.[10]

6-Hydroxy-9-(2'-methylphenyl)-3*H*-xanthen-3-one (Me-Tokyo green, 7)

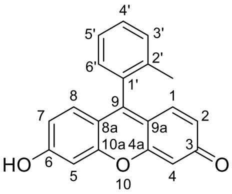

3,6-Dihydroxy-9*H*-xanthen-9-one (0.50 g, 2.19 mmol, 1.0 eq.) was dissolved in anhydrous *N,N*-dimethylformamide (45 mL) and to this was added *tert*-butyldimethylsilyl chloride (1.98 g, 13.1 mmol, 6.0 eq.) and imidazole (1.49 g, 21.9 mmol, 10 eq.). After stirring at room temperature for 4 hours, the reaction mixture was diluted with toluene (100 mL), washed with water (5 × 100 mL), dried (sodium sulfate), and filtered. Concentration *in vacuo* gave 3,6-bis((*tert*-butyldimethylsilyl) oxy)-9*H*-xanthen-9-one as an off-white solid (1.00 g) which was used without further purification.

O-Tolyl magnesium bromide solution (1.82 mL, 3.65 mmol, 1.5 eq.) was cooled to 0 °C, then (6.0 mL, 0.30 mmol) of 3,6-bis((*tert*-butyldimethylsilyl) oxy)-9*H*-xanthen-9-one in anhydrous tetrahydrofuran was added, and the mixture was stirred for 25 minutes. After this time aqueous hydrochloric acid solution (95 mL, 2 M solution) was added, and the suspension was stirred for 30 minutes. The resulting yellow precipitate was collected by filtration, washed with aqueous hydrochloric acid solution (4 × 25 mL, 2 M solution), cold tetrahydrofuran (2 × 50 mL), and concentrated *in vacuo.* The crude material was purified using silica gel column chromatography eluting with dichloromethane:methanol (gradient 0 to 10% methanol) to give the title compound as an orange solid (648 mg, 88%): R_f 0.18 (dichloromethane:methanol, 95:5); m.p. 225–227 °C (from water); ^{1}H NMR (400 MHz, D_6-DMSO) δ 7.60 (dd, *J* = 7.5, 1.4 Hz, 1H, *H-6'*), 7.56 – 7.53 (m, 1H, *H-4'*), 7.49 – 7.48 (m 1H, *H-5'*), 7.37 – 7.16 (m, 5H, *H-1, H-5, H-7, H-8 & H-3'*), 7.16 – 7.08 (m, 2H, *H-2 & H-4*), 2.01 (s, 3H, C*H*$_3$); LRMS *m/z* (ESI$^+$) 303 ([M+H]$^+$, 100%). Analytical HPLC: Dionex Acclaim® 120 C18 column [5 µm, 12 Å, 150 mm × 4.6 mm]; [95:5 H_2O: MeCN → 5:95 H_2O: MeCN: H_2O with 0.1% TFA modifier, 10 min; 5 min hold; 1.5 mLmin^{-1}] Ret. Time = 7.8 min. Purity: 98.4%. The spectroscopic data are in good agreement with literature values.[11]

3-(1-((6,8-Dichloro-9,9-dimethyl-7-oxo-7,9-dihydroacridin-2-yl)oxy)ethyl)-5-methoxy-1,2-dimethyl-1*H*-indole-4,7-dione (1)

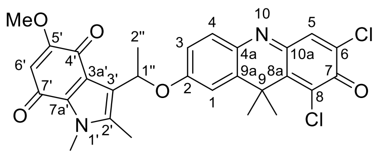

To a solution of compound **5** (45.0 mg, 0.181 mmol, 1.0 eq.) and 1,3-dichloro-7-hydroxy-9,9-dimethylacridin-2(9*H*)-one (138 mg, 0.450 mmol, 2.5 eq.) in anhydrous tetrahydrofuran (2 mL) under argon, was added a solution of diisopropyl azodicarboxylate (0.107 mL, 0.542 mmol, 3.0 eq.) and triphenylphosphine (142 mg, 0.542 mmol, 3.0 eq.) in anhydrous tetrahydrofuran (2 mL) under argon. The solution was stirred at room temperature for 42 hours under an atmosphere of argon. After this time the solvent was evaporated *in vacuo*, and the residue was dissolved in ethyl acetate (50 mL), washed with aqueous sodium hydrogen carbonate (2 × 200mL), water (2 × 100 mL), brine (2 × 50 mL), dried (sodium sulfate), and concentrated *in vacuo*. The crude material was purified using silica gel column chromatography eluting with petroleum ether:ethyl acetate (gradient 10 to 100% ethyl acetate). Then neutral alumina column chromatography eluting with

[10] *Angew. Chem.* **1991**, *103*, 1694-1696
[11] *J. Am. Chem. Soc.* **2005**, *127*, 4888-4894

petroleum ether:ethyl acetate (gradient 10 to 40% ethyl acetate). The residue was dissolved in dichloromethane (2 mL) and precipitated by adding petroleum ether (50 mL). The red precipitate was filtered collected and the precipitation procedure was repeated twice. The precipitate was then dried *in vacuo* to give the title compound as an orange solid (43.5 mg, 15%): R_f 0.4 (petroleum ether:ethyl acetate 1:1); m.p. 133–135 ºC (from petroleum ether); $\bar{\nu}$ max (thin film)/cm^{-1} 2360 (N=C, s), 2345 (m),1640 (C=O, w)), 1263 (C-O, w), 1031; ^{1}H NMR (600 MHz, $CDCl_3$) δ 7.59 (s, 1H, *H-5*), 7.48 (d, *J* = 8.8 Hz, 1H, *H-4*), 7.03 (d, *J* = 2.6 Hz, 1H, *H-1*), 6.86 (dd, *J* = 8.8, 2.6 Hz, 1H, *H-3*), 6.40 (q, *J* = 6.5 Hz, 1H, *H-1''*), 5.64 (s, 1H, *H-6'*), 3.86 (s, 3H, O*CH₃*), 3.82 (s, 3H, N*CH₃*), 2.33 (s, 3H, (C-2')*CH₃*)), 1.82 (s, 3H, (C-9)*CH₃*), 1.71 (s, 3H, (C-9)*CH₃*), 1.68 (d, *J* = 6.5 Hz, 3H, *H-2''*); ^{13}C NMR (151 MHz, $CDCl_3$) δ 178.9 (C-7'), 178.5 (C-4'), 173.3 (C-7), 161.4 (C-2), 159.6 (C-5'), 147.5 (C-3a'), 140.9 (C-8a), 140.8 (C-10a), 139.5 (C-5), 136.7 (C-4a), 135.9 (C-6), 135.7 (C-2'), 134.5 (C-8), 134.2 (C-4), 128.6 (C-7a'), 122.96 (C-3'), 120.2 (C-9a), 115.2 (C-3), 113.8 (C-1), 107.0 (C-6'), 68.95 (C-1''), 56.7 (O*C*H_3), 39.3 (C-9), 32.3 (N*CH₃*), 26.9 (C-9)*C*H_3), 26.66 (C-9)*C*H_3), 22.1 (C-2''), 10.7 ((C-2')*C*H_3); HRMS *m/z* (ESI$^+$) [Found: 561.0955, $C_{28}H_{24}Cl_2N_2NaO_5$ requires [M+Na]$^+$ 561.0954); LRMS *m/z* (ESI$^+$) 540 ([M+H]$^+$, 100%); Analytical HPLC (Dionex Acclaim™ 120 C18 column [5 µm, 120 Å, 4.6 × 150 mm]; 95:5 H_2O: MeCN → 5:95 H_2O: MeCN: H_2O with 0.1% TFA modifier, 10 min; 5 min hold; 1.5 mL min^{-1}] Ret. Time = 13.2 min. Purity: 96.5%.

5'-Methoxy-1',2'-dimethyl-3-(1-((3-oxo-3*H*-phenoxazin-7-yl)oxy)ethyl)-1*H*-indole-4',7'-dione (2)

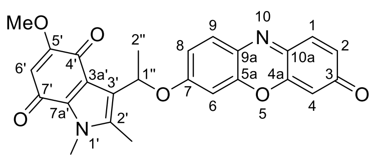

Diisopropyl azodicarboxylate (0.120 mL, 0.602 mmol, 3.0 eq.) was added dropwise to a solution of compound **5** (50.0 mg, 0.201 mmol, 1.0 eq.), resorufin (107 mg, 0.502 mmol, 2.5 eq.) and triphenylphosphine (132 mg, 0.502 mmol, 2.5 eq.) in anhydrous tetrahydrofuran (3 mL) at room temperature under argon. The reaction mixture was stirred vigorously for 24 hours at 50 °C, then concentrated *in vacuo*. The residue was then dissolved in ethyl acetate (50 mL), washed with aqueous sodium hydrogen carbonate (3 × 20 mL), water (3 × 20 mL), aqueous hydrochloric acid (50 mL, 1 M solution), brine (2 × 20 mL), dried (sodium sulfate), and concentrated *in vacuo*. The crude material was purified using silica gel column chromatography eluting with petroleum ether:ethyl acetate (gradient 10 to 100% ethyl acetate). The residue was dissolved in ethyl acetate (2.5 mL) and precipitated by adding hexane (50 mL). The orange precipitate was filtered, washed with hexane (50 mL), and the precipitation procedure was repeated twice. The precipitate was then dried *in vacuo* to give the title compound as an orange solid (36 mg, 40%): R_f 0.22 (petroleum ether:ethyl acetate, 1:1); m.p. 78 °C (hexane); $\bar{\nu}$max (thin film)/cm^{-1}: 2360 (N=C, s), 2341 (m),1636 (C=O, w)), 1263 (C-O, w); ^{1}H NMR (600 MHz, $CDCl_3$) δ 7.62 (d, *J* = 8.9 Hz, 1H, *H-9*), 7.38 (d, *J* = 9.8 Hz, 1H, *H-1*), 6.92 (dd, *J* = 8.9, 2.6 Hz, 1H, *H-8*), 6.80 (dd, *J* = 9.8, 2.0 Hz, 1H, *H-2*), 6.78 (d, *J* = 2.6 Hz, 1H, *H-6*), 6.36 (q, *J* = 6.5 Hz, 1H, *H-1''*), 6.29 (d, *J* = 2.0 Hz, 1H, *H-4*), 5.66 (s, 1H, *H-6'*), 3.86 (s, 3H, O*CH₃*), 3.82 (s, 3H, N*CH₃*), 2.30 (s, 3H, (C-2')*CH₃*)), 1.68 (d, *J* = 6.5 Hz, 3H, *H-2''*); ^{13}C NMR (151 MHz, $CDCl_3$) δ 186.3 (C-3), 178.7 (C-4'), 178.5 (C-4), 161.8 (C-7), 159.5 (C-5'), 149.8 (C-4a), 145.6 (C-10a), 145.5(C-5a), 135.2 (C-1), 134.6 (C-2), 134.2 (C-2'), 131.6 (C-9), 128.7 (C-9a), 128.4 (C-7a'), 122.4 (C-3'), 120.0 (C-3a'), 114.4 (C-8), 106.95 (C-6'), 106.7 (C-4), 101.7 (C-6), 69.8 ,(C-1'') 56.6 (O*C*H_3), 32.1 (N*CH₃*), 22.1 (C-2''), 10.5 ((C-2')*C*H_3); HRMS *m/z* (ESI$^+$) [Found: 467.1213, $C_{25}H_{20}N_2NaO_6$ requires [M+Na]$^+$ 467.1214); LRMS *m/z* (ESI$^+$) 445 ([M+H]$^+$, 90%); Analytical HPLC (Dionex Acclaim™ 120 C18 column [5 µm, 120 Å, 4.6 × 150 mm]; 95:5 H_2O: MeCN → 5:95 H_2O: MeCN: H_2O with 0.1% TFA modifier, 10 min; 5 min hold; 1.5 mL min^{-1}] Ret. Time = 10.6 min. Purity: 93.2%.

5-Methoxy-1,2-dimethyl-3-(1-((3-oxo-9-(*O*-tolyl)-3*H*-xanthen-6-yl)oxy)ethyl)-1*H*-indole-4,7-dione (3)

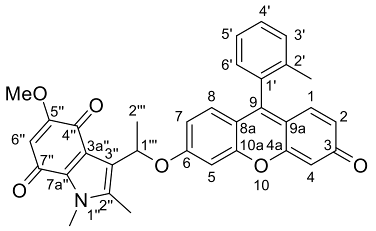

Diisopropyl azodicarboxylate (0.178 mL, 0.903 mmol, 3.0 eq.) was added dropwise to a solution of compound **5** (75.0 mg, 0.301 mmol, 1.0 eq.), 6-hydroxy-9-(2'-methylphenyl)-3*H*-xanthen-3-one (182 mg, 0.602 mmol, 2.0 eq.) and triphenylphosphine (237 mg, 0.903 mmol, 3.0 eq.) in anhydrous tetrahydrofuran (2 mL) at room temperature and under argon. The reaction mixture was stirred vigorously for 24 hours at 50 °C, then concentrated *in vacuo*. The residue was dissolved in ethyl acetate (20 mL), washed with saturated aqueous sodium hydrogen carbonate (3 × 100 mL), water (3 × 100 mL), aqueous hydrochloric acid (100 mL, 1 M solution), brine (2 × 20 mL), dried (sodium sulfate), and concentrated *in vacuo*. The crude material was purified using neutral alumina column chromatography eluting with petroleum ether:ethyl acetate (gradient 10 to 100% ethyl acetate), then with silica gel column chromatography using a Biotage system with dichloromethane:methanol (gradient 0 to 15% methanol). The residue was dissolved in ethyl acetate (2.5 mL) and precipitated by adding hexane (50 mL). The orange precipitate was filtered, washed with hexane (50 mL), and the precipitation procedure was repeated twice. The precipitate was then dried *in vacuo* to give the title compound as an orange/red solid (80 mg, 50%): R_f 0.43 (ethyl acetate), m.p. 140 ºC (dec., from hexane); $\bar{\nu}$max (thin film)/cm^{-1}: 2360 (N=C, w), 2341 (s), 1638 (C=O, m), 1595 (s), 1207 (C-O), 753 (m); ^{1}H NMR (600 MHz, $CDCl_3$) δ 7.44 – 7.40 (m, 2H, Rotamer A&B, *H-5'*), 7.39 – 7.35 (m, 2H, Rotamer A&B, *H-6'*), 7.34 – 7.31 (m, 2H, Rotamer A&B, *H-4'*), 7.13 – 7.10 (m, 2H, Rotamer A&B, *H-3'*), 6.91 – 6.89 (m, 3H, *H-5, H-8, H-1*, Rotamer A), 6.89 – 6.86 (m, 3H, *H-5, H-8, H-1*, Rotamer B), 6.79 – 6.73 (m, 1H, *H-7*, Rotamer A), 6.74 – 6.72 (m, 1H, *H-7*, Rotamer B), 6.54 – 6.53 (m, 1H, *H-2*, Rotamer A), 6.52 – 6.51 (m, 1H, *H-2*, Rotamer B), 6.41 – 6.39 (m, 2H, *H-4*, Rotamer A&B), 6.35 (q, *J* = 6.5 Hz, 1H, *H-1'''*, Rotamer A), 6.34 (q, *J* = 6.5 Hz, 1H, *H-1'''*, Rotamer B), 5.64 (s, 1H, *H-6''*, Rotamer A), 5.63 (s, 1H, *H-6''*, Rotamer B), 3.84 (s, 3H, OCH_3, Rotamer A), 3.83 (s, 3H, OCH_3, Rotamer B), 3.81 (s, 3H, NCH_3, Rotamer A), 3.81 (s, 3H, NCH_3, Rotamer B), 2.30 (s, 3H, (C-2')CH_3), Rotamer A), 2.29 (s, 3H, (C-2')CH_3), Rotamer B), 2.03 (s, 3H, Rotamer A, (C-2'')CH_3)), 2.02 (s, 3H, Rotamer B, (C-2'')CH_3)), 1.67 (d, *J* = 6.5 Hz, 6H, Rotamer A&B, *H-2'''*). ^{13}C NMR (151 MHz, $CDCl_3$) δ 185.9 (Rotamer A, C-3), 185.9 (Rotamer B, C-3), 178.7 (Rotamer A, C-7''), 178.6 (Rotamer B, C-7''), 178.5 (Rotamer A&B, C-4''), 162.6 (Rotamer A&B, C-6), 159.6 (Rotamer A, C-5''), 159.6 (Rotamer B, C-5''), 159.0 (Rotamer A, C-4a), 159.0 (Rotamer B, C-4a), 154.6 (Rotamer A, C-10a), 154.5 (Rotamer B, C-10a), 149.5 (Rotamer A, C-9), 149.5 (Rotamer B, C-9), 136.2 (Rotamer A, C-2''), 136.2 (Rotamer B, C-2''), 135.3 (Rotamer A, C-1'), 135.3 (Rotamer B, C-1'), 132.6 (Rotamer A&B, C-2'), 130.6 (Rotamer A, C-1), 130.6 (Rotamer B, C-1), 130.1 (Rotamer A, C-2), 130.1 (Rotamer B, C-2), 129.6 (Rotamer B, C-5'), 129.6 (Rotamer B, C-5'), 129.5 (Rotamer A&B, C-7a''), 129.2 (Rotamer A, C-3'), 129.1 (Rotamer B, C-3') 128.7 (Rotamer A, C-8), 128.7 (Rotamer B, C-8), 126.1 (Rotamer A&B, C-4'), 122.5 (Rotamer A, C-3a''), 122.5 (Rotamer B, C-3a''), 120.1(Rotamer A, C-3''), 120.1 (Rotamer B, C-3''), 118.3 (Rotamer A, C-9a), 118.2 (Rotamer B, C-9a), 114.5 (Rotamer A, C-8a), 114.5 (Rotamer A, C-8a), 113.8 (Rotamer A, C-7), 113.4 (Rotamer B, C-7), 107.0 (Rotamer A, C-6''), 107.0 (Rotamer B, C-6''), 105.9 (Rotamer A, C-4), 105.9 (Rotamer B, C-4), 102.7 (C-5), 102.4 (C-5), 69.8 (Rotamer A, C-1'''), 69.7 (Rotamer B, C-1'''), 56.7 (Rotamer A, OCH_3), 56.7 (Rotamer B, OCH_3), 32.2 (Rotamer A&B, NCH_3), 22.2 (Rotamer A&B C-2''), 19.7 (Rotamer A (C-2')CH_3), 19.7 (Rotamer B (C-2')CH_3), 10.6 (Rotamer A&B (C-2'')CH_3). HRMS *m/z* (ESI$^+$) [Found: 534.1910, $C_{33}H_{27}NO_6$ requires $[M+H]^+$ 534.1911); LRMS *m/z* (ESI$^+$) 534 ($[M+H]^+$, 93%); Analytical HPLC (Agilent 1260 Infinity II® Poroshell 120 EC-C18 column

[4 µm, 4.6 × 100 mm]; [95:5 H_2O: MeCN → 5:95 H_2O: MeCN: H_2O with 0.1% FA modifier, 10 min; 5 min hold; 1 mL min^{-1}]. Ret. Time = 9.8 min. Purity: 91.5%.

5-Methoxy-1,2-dimethyl-3-(((3-oxo-9-(*O*-tolyl)-3*H*-xanthen-6-yl)oxy)methyl)-1*H*-indole-4,7-dione (4)

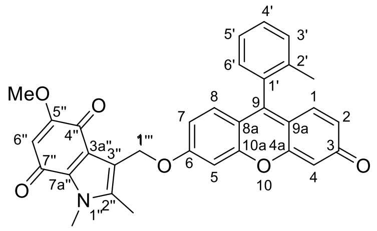

6-Hydroxy-9-(2'-methylphenyl)-3*H*-xanthen-3-one (42.0 mg, 0.138 mmol, 1.0 eq.), 3-(chloromethyl)-5-methoxy-1,2-dimethyl-1*H*-indole-4,7-dione (35.0 mg, 0.138 mmol, 1.0 eq.), cesium carbonate (90.0 mg, 0.276 mmol, 2.0 eq.) and tetrabutyl ammonium iodide (51.0 mg, 0.138 mmol, 1.0 eq.) were dissolved in anhydrous *N,N*-dimethylformamide (2 mL) at 0 °C under argon. The mixture was stirred at room temperature for 20 hours, after which time saturated aqueous ammonium chloride (50 mL) was added, and the mixture was extracted with ethyl acetate (3 × 50 mL). The organic components were washed with saturated aqueous bicarbonate (6 × 50 mL), water (2 × 50 mL), dried (sodium sulfate), and concentrated *in vacuo*. The crude material was purified using silica gel column chromatography eluting petroleum ether:ethyl acetate (gradient 0 to 100% ethyl acetate) then acetone. The residue was dissolved in chloroform (2.5 mL) and precipitated by adding hexane (50 mL). The orange precipitate was filtered, washed with hexane (50 mL), and the precipitation procedure was repeated twice. The precipitate was then dried *in vacuo* to give the title compound as an orange solid (49 mg, 68%): R_f 0.25 (ethyl acetate), m.p. 135–138 ºC (from hexane); $\bar{\nu}$max (thin film)/cm^{-1}: 2360 (N=C, s), 2341 (s), 1638 (C=O, m), 771 (s); ^{1}H NMR (600 MHz, $CDCl_3$) δ 7.46 – 7.43 (m, 1H, *H-5'*), 7.38 (d, *J* = 7.6 Hz, 1H, *H-6'*), 7.37 – 7.34 (m, 1H, *H-4'*), 7.15 (dd, *J* = 7.6, 1.4 Hz, 1H, *H-3'*), 7.09 (d, *J* = 2.4 Hz, 1H, *H-5*), 6.95 (d, *J* = 8.9 Hz, 1H, *H-8*), 6.93 (d, *J* = 9.7 Hz, 1H, *H-1*), 6.82 (dd, *J* = 8.9, 2.4 Hz, 1H, *H-7*), 6.56 (dd, *J* = 9.7, 1.9 Hz, 1H, *H-2*), 6.45 (d, *J* = 1.9 Hz, 1H, *H-4*), 5.64 (s, 1H, *H-6''*), 5.43 – 5.36 (m, 2H, *H-1'''*), 3.91 (s, 3H, OCH_3), 3.81 (s, 3H, NCH_3), 2.32 (s, 3H, (C-2'')CH_3), 2.06 (s, 3H, (C-2')CH_3); ^{13}C NMR (151 MHz, $CDCl_3$) δ 185.9 (C-3), 178.8 (C-7''), 178.3 (C-4''), 163.4 (C-6), 159.7 (C-5''), 159.1 (C-4a), 154.7 (C-10a), 149.5 (C-9), 138.2 (C-2''), 136.3 (C-1'), 132.7 (C-2'), 130.70 (C-1), 130.6 (C-6'), 130.2 (C-2), 129.6 (C-5'), 129.6 (C-7a''), 129.2 (C-3'), 129.2 (C-8), 126.2 (C-4'), 121.4 (C-3a''), 118.4 (C-9a), 115.8 (C-3''), 114.6 (C-8a) , 113.7 (C-7), 106.9 (C-6''), 106.0 (C-4), 101.9 (C-5), 61.3 (C-1'''), 56.6 (OCH_3), 32.6 (NCH_3), 19.7 ((C-2')CH_3), 10.0 ((C-2'')CH_3). HRMS *m/z* (ESI$^+$) [Found: 520.1752, $C_{32}H_{25}NO_6$ requires [M+H]$^+$ 520.1755); LRMS *m/z* (ESI$^+$) 520 ([M+H]$^+$ 100%); Analytical HPLC (Dionex Acclaim™ 120 C18 column [5 µm, 120 Å, 4.6 × 150 mm]; 95:5 H_2O: MeCN → 5:95 H_2O: MeCN: H_2O with 0.1% TFA modifier, 10 min; 5 min hold; 1.5 mL min^{-1}] Ret. Time = 9.7 min. Purity: 97.3%.

5'-Methoxy-1',2'-dimethyl-3'-(1''-((4-methyl-2-oxo-2*H*-chromen-7-yl)oxy)ethyl)-1*H*-indole-4',7'-dione (11)

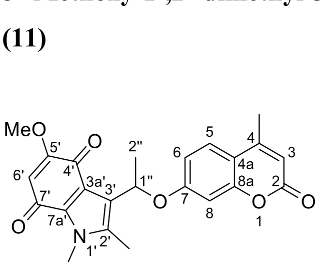

Diisopropyl azodicarboxylate (0.12 mL, 0.60 mmol, 3.0 eq.) was added dropwise to a solution of compound **5** (50.0 mg, 0.20 mmol, 1.0 eq.), 7-hydroxy-4-methyl coumarin (88.0 mg, 0.50 mmol, 2.5 eq.) and triphenylphosphine (131.5 mg, 0.50 mmol, 2.5 eq.) in anhydrous tetrahydrofuran (1.25 mL) at room temperature and under argon. The solution was stirred vigorously for 2.5 hours, then concentrated *in vacuo*. The residue was dissolved in ethyl acetate (50 mL) and washed with aqueous sodium hydroxide solution (50 mL, 1 M). The organic components were washed with aqueous hydrochloric acid solution (50 mL, 1 M), brine (50 mL), dried (sodium

sulfate), filtered, and concentrated *in vacuo*. The crude material was purified using silica gel column chromatography eluting with petroleum ether:ethyl acetate (15 to 100% gradient ethyl acetate). The residue was dissolved in ethyl acetate (5 mL) and precipitated by adding hexane (50 mL). The orange precipitate was filtered, washed with hexane (50 mL) and the precipitation procedure was repeated twice. The precipitate was dried *in vacuo* to give the title compound as orange solid (27 mg, 33%): R_f 0.1 (petroleum ether:ethyl acetate, 3:7); m.p. 195–198 °C (from ethyl acetate) [lit.[12] 213–215 °C]. ^{1}H NMR (400 MHz, $CDCl_3$) δ 7.41 (d, *J* = 8.8 Hz, 1H, *H-5*), 6.85 (dd, *J* = 8.8, 2.5 Hz, 1H, *H-6*), 6.73 (d, *J* = 2.5 Hz, 1H, *H-8*), 6.28 (q, *J* = 6.5 Hz, 1H, *H-1''*)), 6.07 (q, *J* = 1.2 Hz, 1H, *H-3*), 5.64 (s, 1H, (*H-6'*), 3.84 (s, 3H, OCH_3), 3.79 (s, 3H, NCH_3), 2.34 (d, *J* = 1.2 Hz, 3H, (C-4)CH_3)), 2.27 (s, 3H, (C-2')CH_3)), 1.65 (d, *J* = 6.5 Hz, 3H, (*H-2''*)). LRMS *m/z* (ESI$^+$) 408 ([M+H]$^+$, 6.74%), 430 ([M+Na]$^+$, 12.09%), 815 ([2M+H]$^+$, 32.66%); 837 ([2M+Na]$^+$,100.00%). The spectroscopic data are in good agreement with literature values.[12]

[12] *Biochem. Pharm.* **2002**, *63*, 1629-1639

8. NMR spectra

^{1}H NMR. 3-(1-((6,8-Dichloro-9,9-dimethyl-7-oxo-7,9-dihydroacridin-2-yl)oxy)ethyl)-5-methoxy-1,2-dimethyl-1*H*-indole-4,7-dione (1)

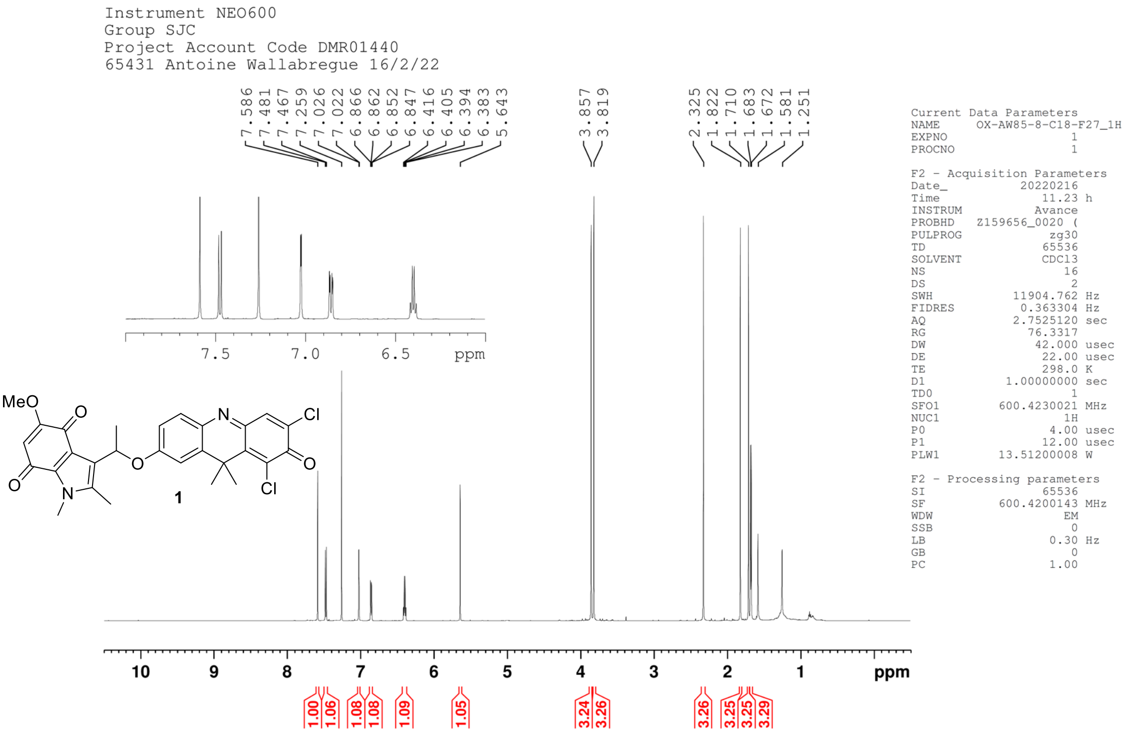

^{13}C NMR. 3-(1-((6,8-Dichloro-9,9-dimethyl-7-oxo-7,9-dihydroacridin-2-yl)oxy)ethyl)-5-methoxy-1,2-dimethyl-1*H*-indole-4,7-dione (1)

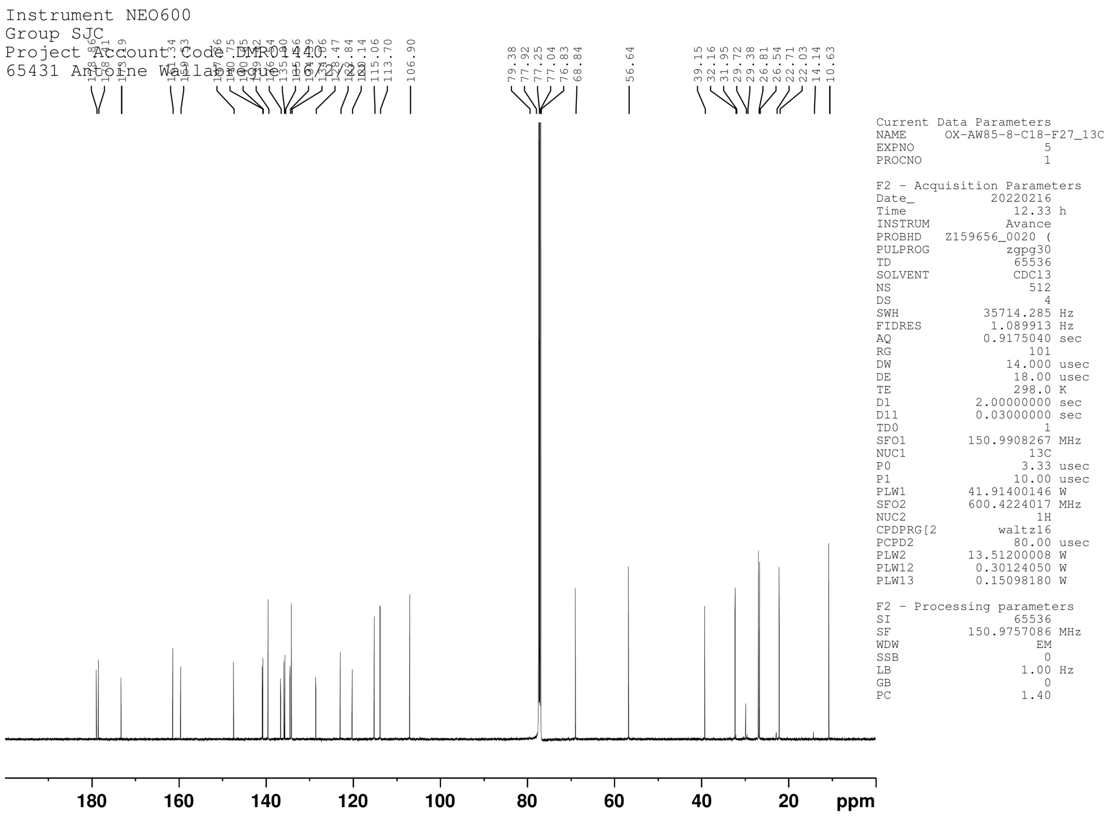

NOESY. **3-(1-((6,8-Dichloro-9,9-dimethyl-7-oxo-7,9-dihydroacridin-2-yl)oxy)ethyl)-5-methoxy-1,2-dimethyl-1*H*-indole-4,7-dione (1)**

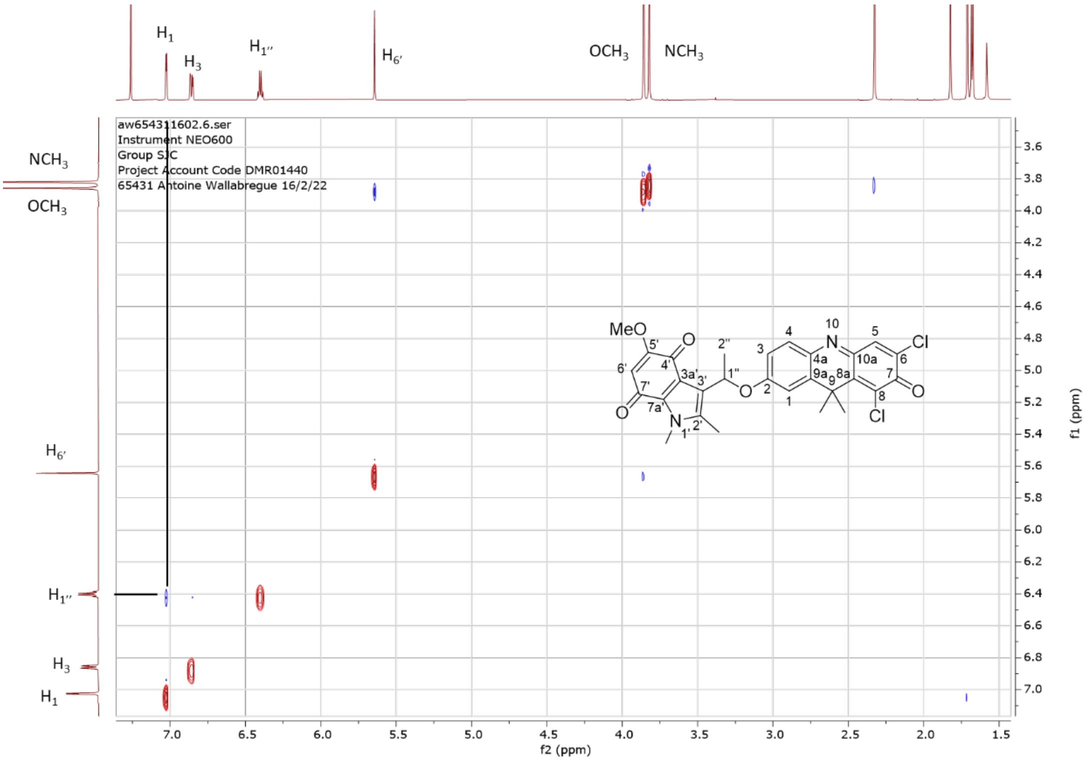

HMBC. **3-(1-((6,8-Dichloro-9,9-dimethyl-7-oxo-7,9-dihydroacridin-2-yl)oxy)ethyl)-5-methoxy-1,2-dimethyl-1*H*-indole-4,7-dione (1)**

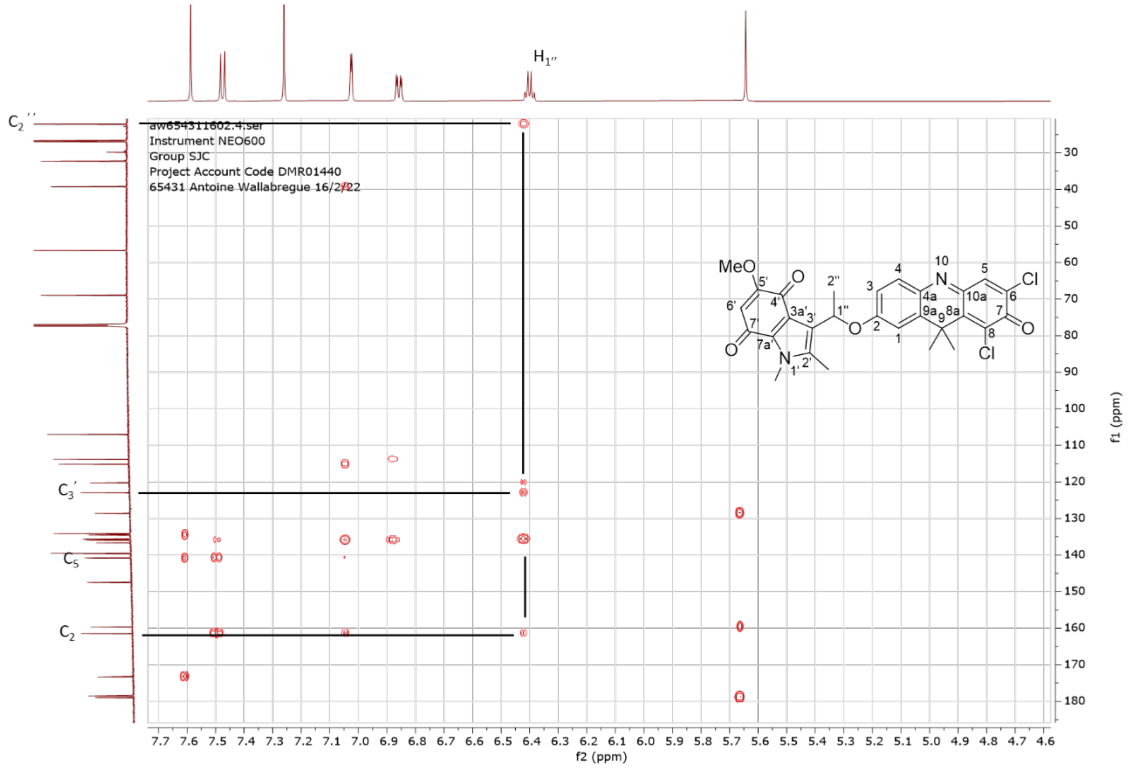

^{1}H NMR. 5'-Methoxy-1',2'-dimethyl-3-(1-((3-oxo-3*H*-phenoxazin-7-yl)oxy)ethyl)-1*H*-indole-4',7'-dione (2)

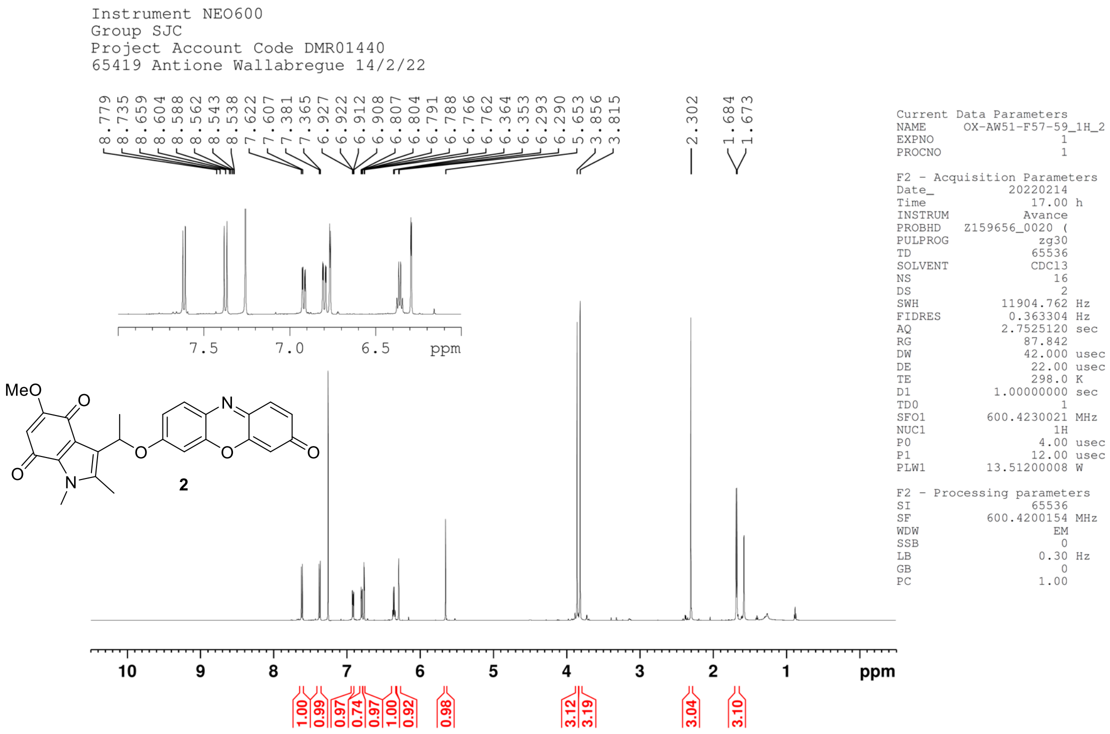

^{13}C NMR. 5'-Methoxy-1',2'-dimethyl-3-(1-((3-oxo-3*H*-phenoxazin-7-yl)oxy)ethyl)-1*H*-indole-4',7'-dione (2)

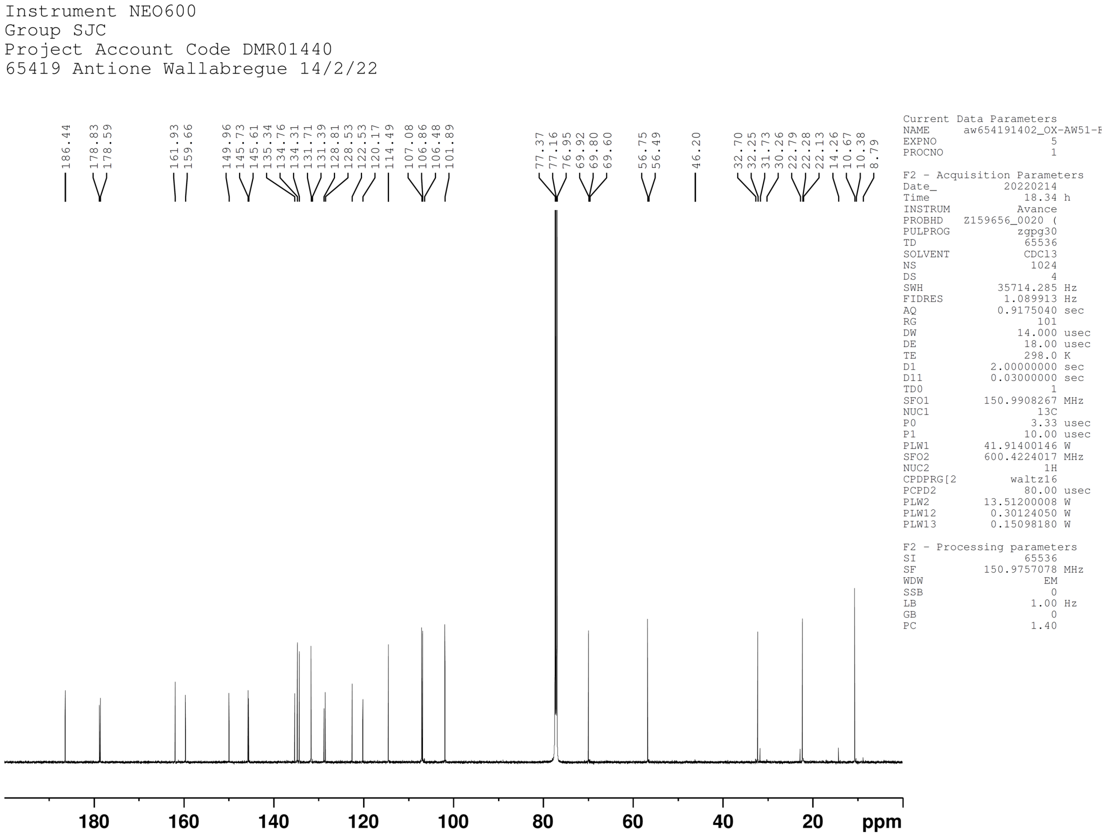

^{1}H NMR. 5-Methoxy-1,2-dimethyl-3-(1-((3-oxo-9-(*O*-tolyl)-3*H*-xanthen-6-yl)oxy)ethyl)-1*H*-indole-4,7-dione (3)

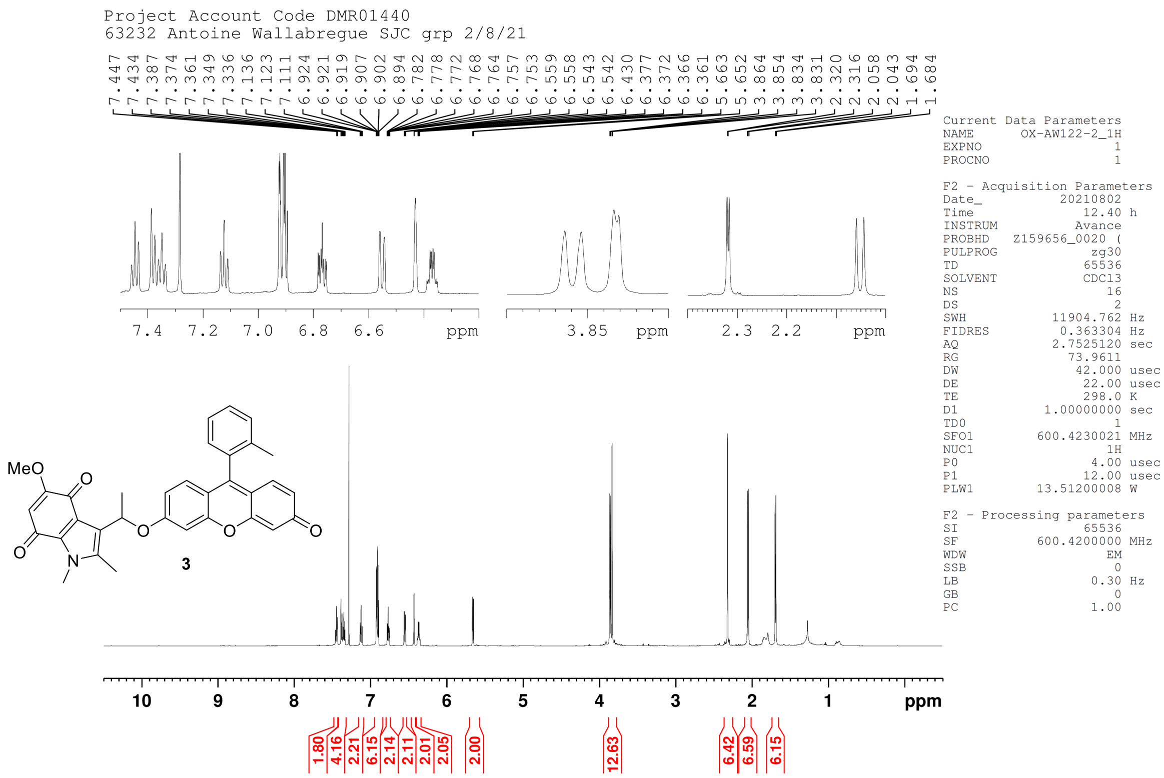

^{13}C NMR. 5-Methoxy-1,2-dimethyl-3-(1-((3-oxo-9-(*O*-tolyl)-3*H*-xanthen-6-yl)oxy)ethyl)-1*H*-indole-4,7-dione (3)

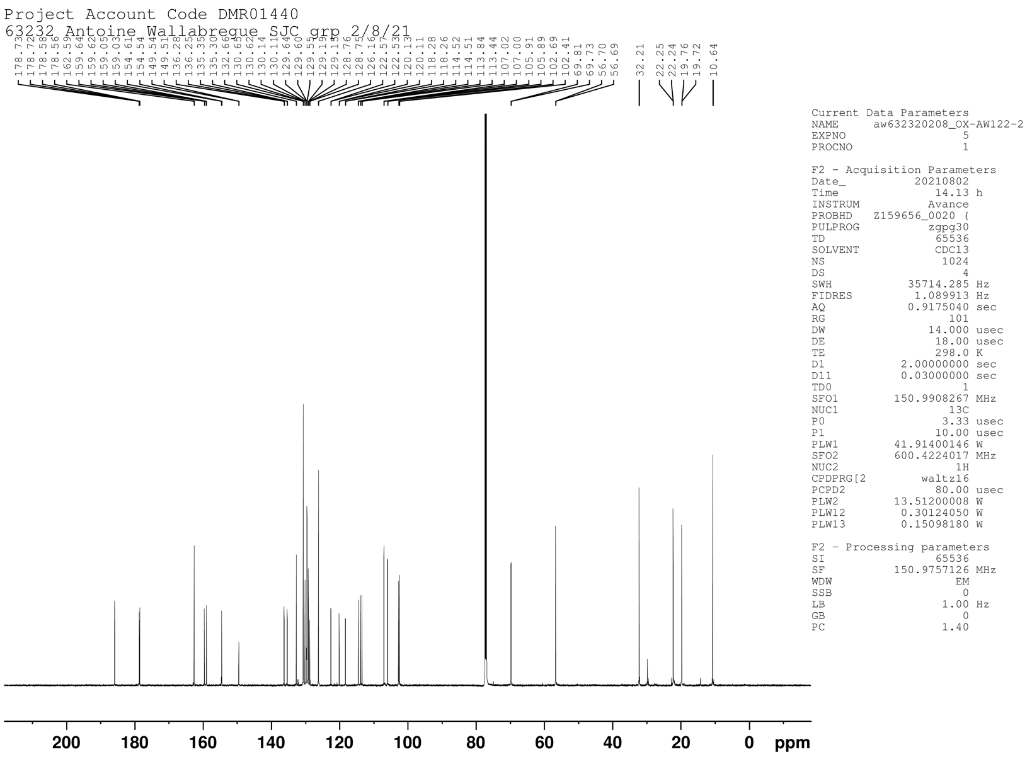

^{1}H NMR. 5-Methoxy-1,2-dimethyl-3-(((3-oxo-9-(*O*-tolyl)-3*H*-xanthen-6-yl)oxy)methyl)-1*H*-indole-4,7-dione (4)

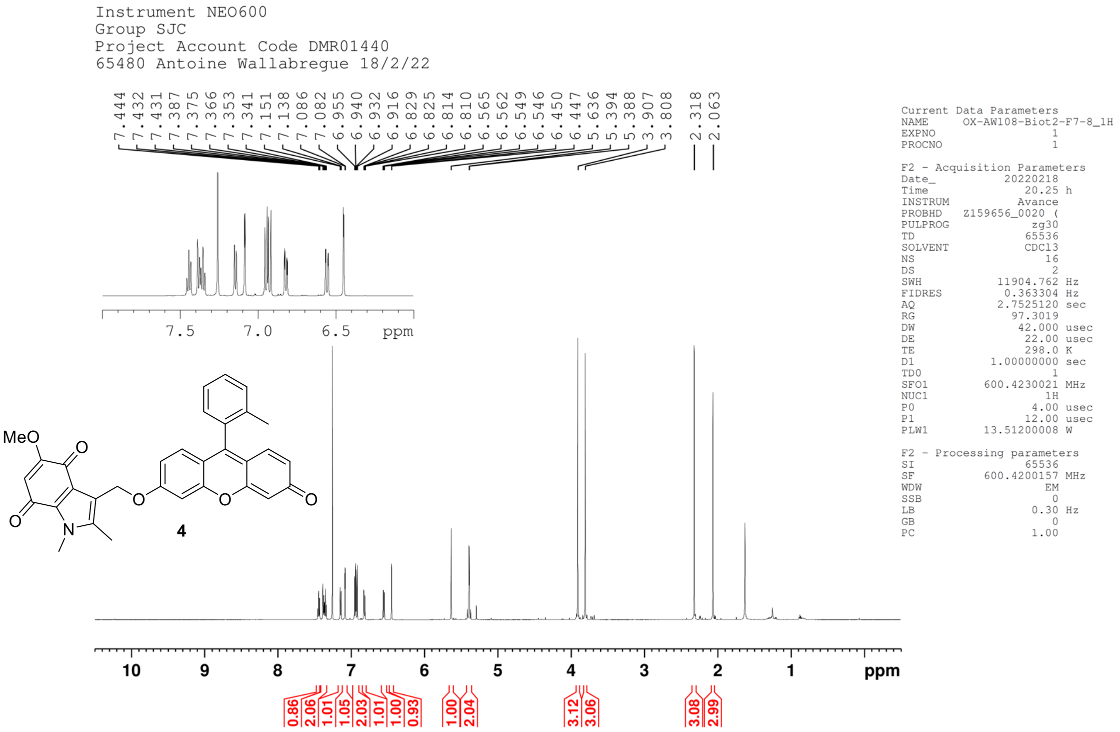

^{13}C NMR. 5-Methoxy-1,2-dimethyl-3-(((3-oxo-9-(*O*-tolyl)-3*H*-xanthen-6-yl)oxy)methyl)-1*H*-indole-4,7-dione (4)

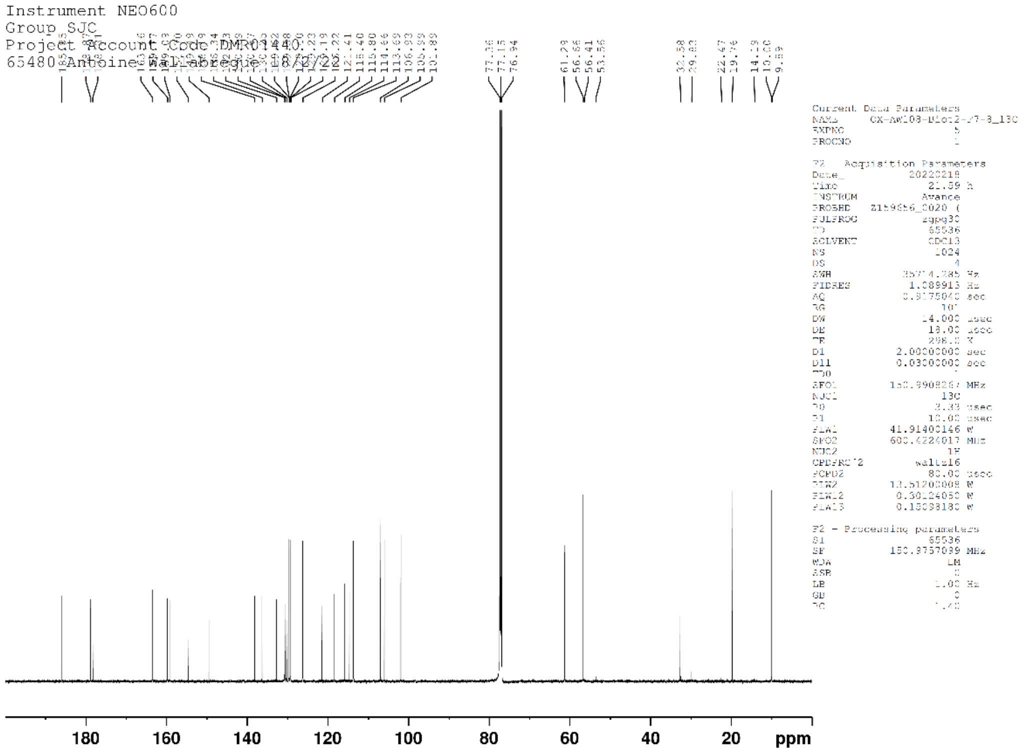

9. HPLC Traces

3-(1-((6,8-Dichloro-9,9-dimethyl-7-oxo-7,9-dihydroacridin-2-yl)oxy)ethyl)-5-methoxy-1,2-dimethyl-1*H*-indole-4,7-dione (1) @254nm

OXAW85-6 **10/2/2021**

Acquisition Method	Purity short run @254 nm
Acquisition Date/Time	9/13/2021 5:42 pm
Injection Volume	40
Sample Name	OXAW85-6
Sample Description	
Batch Description	

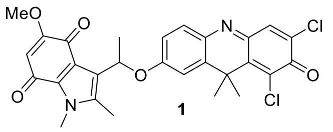

OXAW85-6 : Injection 1

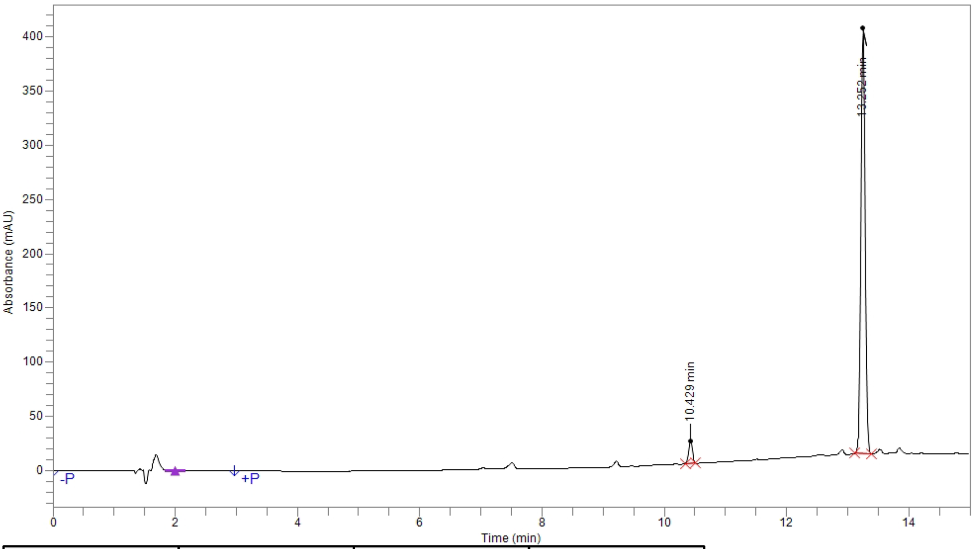

Time	Height	Area	Area %
10.429	20,383.0	83,264.0	4.49
13.252	393,495.6	1,769,114.8	95.51
**Total**		1,852,378.8	100.00

3-(1-((6,8-Dichloro-9,9-dimethyl-7-oxo-7,9-dihydroacridin-2-yl)oxy)ethyl)-5-methoxy-1,2-dimethyl-1*H*-indole-4,7-dione (1) @280nm

Acquisition Method	Purity short run @280 nm
Acquisition Date/Time	9/13/2021 6:03 pm
Injection Volume	40
Sample Name	OXAW85-6
Sample Description	
Batch Description	

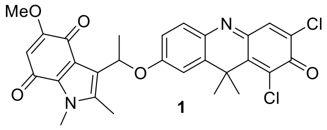

OXAW85-6 : Injection 1

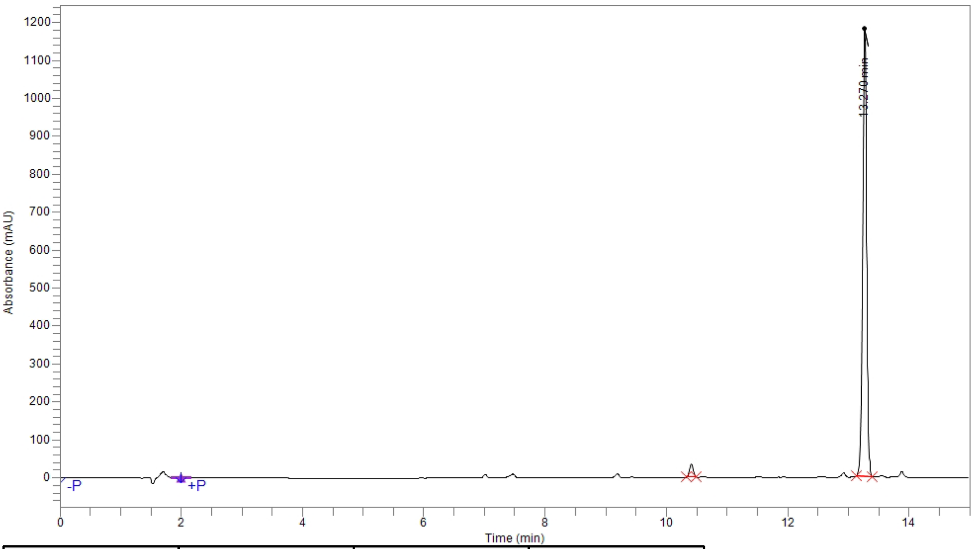

Time	Height	Area	Area %
10.411	35,411.5	140,326.0	2.57
13.270	1,181,818.0	5,328,023.5	97.43
**Total**		5,468,349.5	100.00

5'-Methoxy-1',2'-dimethyl-3-(1-((3-oxo-3*H*-phenoxazin-7-yl)oxy)ethyl)-1*H*-indole-4',7'-dione (2) @254 nm

OXAW51-2 **10/2/2021**

Acquisition Method	Purity short run @254 nm
Acquisition Date/Time	9/13/2021 4:46 pm
Injection Volume	40
Sample Name	OXAW51-2
Sample Description	
Batch Description	

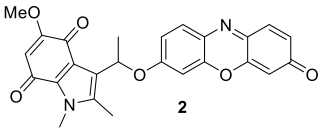

2

OXAW51-2 : Injection 1

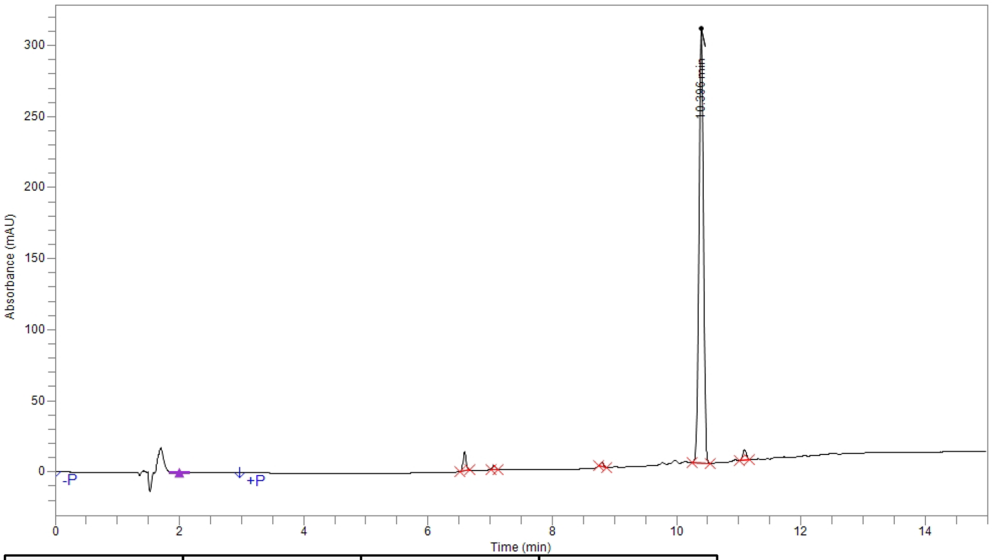

Time	Height	Area	Area %
6.588	13,446.3	47,468.8	3.13
7.065	2,714.3	8,841.8	0.58
8.798	2,791.6	11,067.8	0.73
10.396	305,851.7	1,417,074.4	93.53
11.093	7,270.8	30,670.2	2.02
**Total**		1,515,123.0	100.00

5’-Methoxy-1’,2’-dimethyl-3-(1-((3-oxo-3*H*-phenoxazin-7-yl)oxy)ethyl)-1*H*-indole-4’,7’-dione (2) @280 nm

OXAW51-2 **10/2/2021**

Acquisition Method	Purity short run @280 nm
Acquisition Date/Time	9/13/2021 5:07 pm
Injection Volume	40
Sample Name	OXAW51-2
Sample Description	
Batch Description	

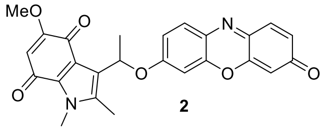

OXAW51-2 : Injection 1

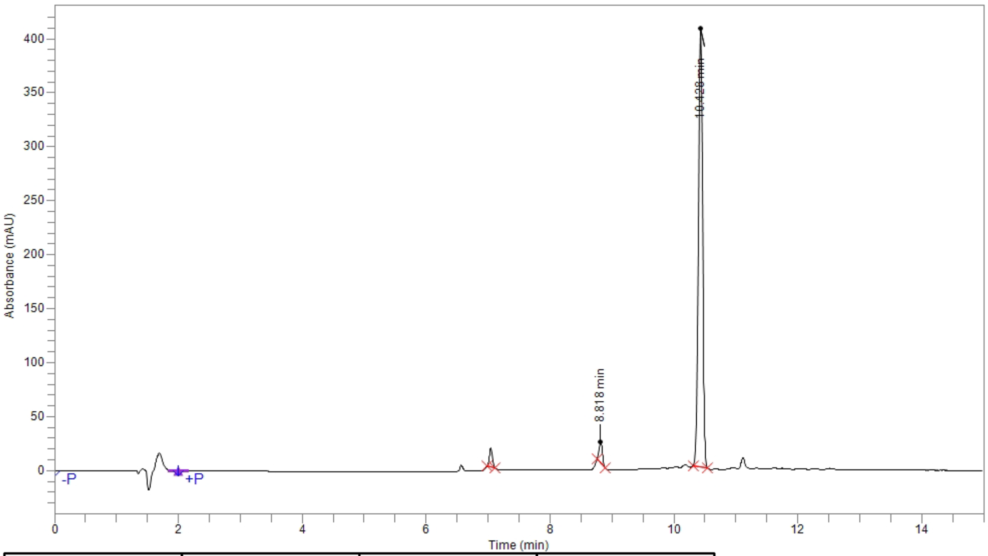

Time	Height	Area	Area %
7.046	17,402.4	58,785.8	2.97
8.818	20,198.7	81,013.5	4.09
10.428	406,839.7	1,839,633.6	92.94
**Total**		1,979,432.9	100.00

5-Methoxy-1,2-dimethyl-3-(1-((3-oxo-9-(*O*-tolyl)-3*H*-xanthen-6-yl)oxy)ethyl)-1*H*-indole-4,7-dione (3) @254 and 280 nm

Data File C:\USERS\P...INE\2022\02\AW122-2 F19 2022-02-21 17-51-55\001-P1-F6-AW122-2 F19.D
Sample Name: AW122-2 F19

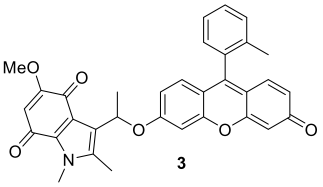

3

===

Acq. Operator : SYSTEM Seq. Line : 1
Sample Operator : SYSTEM
Acq. Instrument : Analytical HPLC Location : P1-F-06
Injection Date : 21/02/2022 5:52 pm Inj : 1
Inj Volume : 10.000 µl
Different Inj Volume from Sample Entry! Actual Inj Volume : 5.000 µl
Acq. Method : C:\Users\Public\Documents\ChemStation\1\Data\AW122-2 F19 2022-02-21 17-51-55\IOD_PURITY_TEST.M
Last changed : 15/11/2021 5:51 pm by SYSTEM
Analysis Method : C:\USERS\PUBLIC\DOCUMENTS\CHEMSTATION\1\METHODS\STANDARD METHODS\SHUTDOWN.M
Last changed : 08/04/2022 5:05 pm by SYSTEM
(modified after loading)
Method Info : Stores the column in 100% MeCN + 0.1% FA (Line B) and turns the system off at end of method

Additional Info : Peak(s) manually integrated

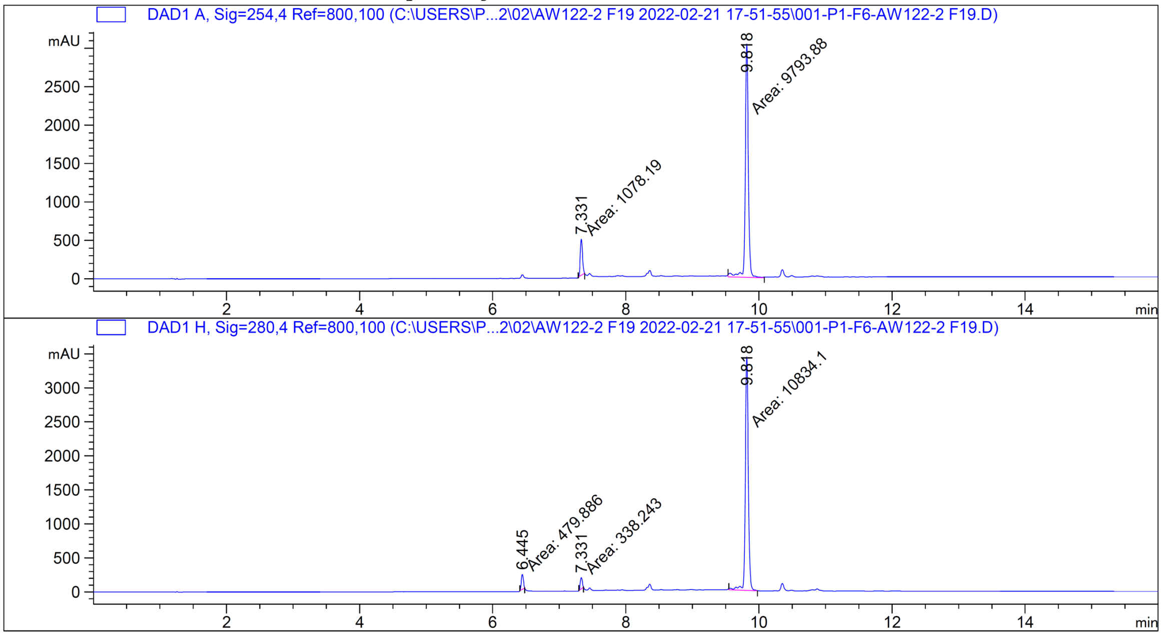

===

Area Percent Report

===

Sorted By : Signal
Multiplier : 1.0000
Dilution : 1.0000
Do not use Multiplier & Dilution Factor with ISTDs

Signal 1: DAD1 A, Sig=254,4 Ref=800,100

Peak #	RetTime [min]	Type	Width [min]	Area [mAU*s]	Height [mAU]	Area %
1	7.331	MM	0.0381	1078.18713	472.20682	9.9170
2	9.818	MM	0.0536	9793.87988	3043.80957	90.0830

Totals : 1.08721e4 3516.01639

Data File C:\USERS\P...INE\2022\02\AW122-2 F19 2022-02-21 17-51-55\001-P1-F6-AW122-2 F19.D
Sample Name: AW122-2 F19

Signal 2: DAD1 H, Sig=280,4 Ref=800,100

Peak #	RetTime [min]	Type	Width [min]	Area [mAU*s]	Height [mAU]	Area %
1	6.445	MM	0.0362	479.88556	221.09337	4.1184
2	7.331	MM	0.0338	338.24295	166.78864	2.9028
3	9.818	MM	0.0525	1.08341e4	3438.86914	92.9788

Totals : 1.16523e4 3826.75114

===

*** End of Report ***

5-Methoxy-1,2-dimethyl-3-(((3-oxo-9-(*O*-tolyl)-3*H*-xanthen-6-yl)oxy)methyl)-1*H*-indole-4,7-dione (4)@220 nm

Acquisition Method	Purity short run @220 nm
Acquisition Date/Time	6/10/2021 1:13 pm
Injection Volume	10
Sample Name	OX-AW108@220
Sample Description	
Batch Description	

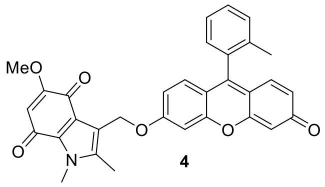

4

OX-AW108@220 : Injection 1

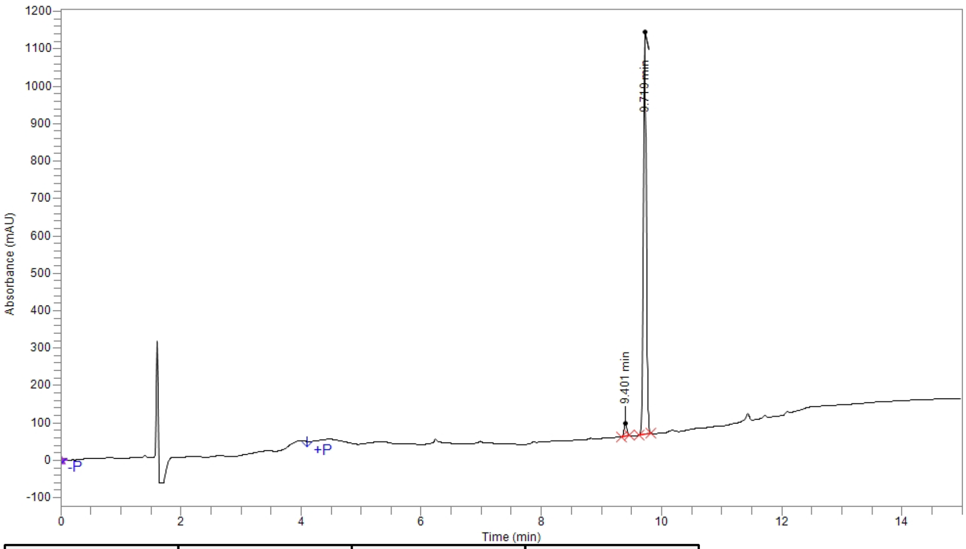

Time	Height	Area	Area %
9.401	33,678.6	121,625.0	3.25
9.719	1,077,043.4	3,616,400.3	96.75
**Total**		3,738,025.3	100.00

5-Methoxy-1,2-dimethyl-3-(((3-oxo-9-(*O*-tolyl)-3*H*-xanthen-6-yl)oxy)methyl)-1*H*-indole-4,7-dione (4) @254 nm

Acquisition Method	Purity short run @254 nm
Acquisition Date/Time	6/10/2021 12:53 pm
Injection Volume	10
Sample Name	OX-AW108@254
Sample Description	
Batch Description	

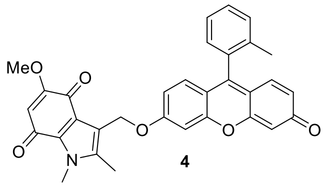

4

OX-AW108@254 : Injection 1

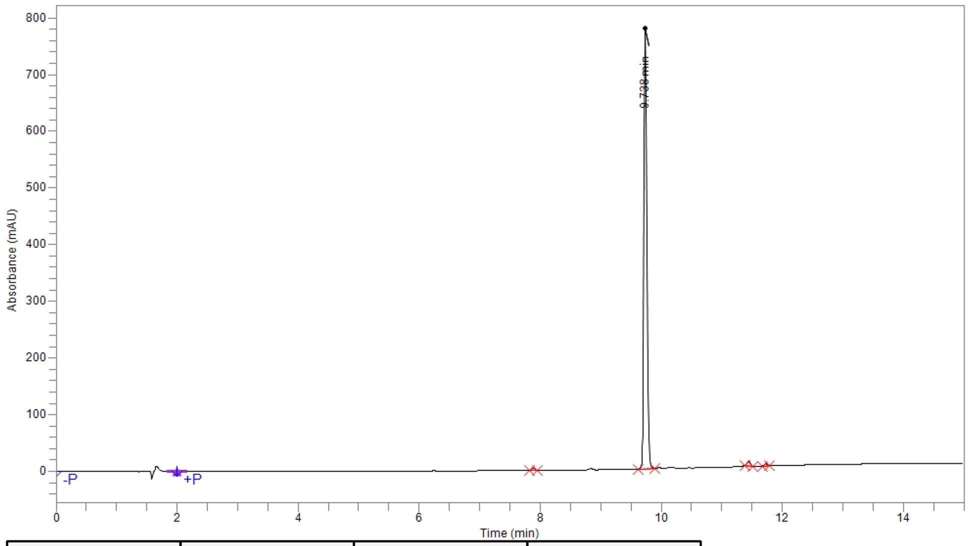

Time	Height	Area	Area %
7.886	3,546.4	13,117.2	0.50
9.738	779,830.6	2,575,207.6	97.84
11.445	8,458.1	29,547.9	1.12
11.729	3,833.7	14,068.6	0.53
**Total**		2,631,941.3	100.00

Compound 5 @220 nm

OX-AW83@220 **6/15/2021**

Acquisition Method	Purity short run @220 nm
Acquisition Date/Time	6/7/2021 6:23 pm
Injection Volume	10
Sample Name	OX-AW83@220
Sample Description	
Batch Description	

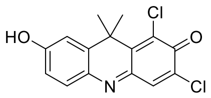

5

OX-AW83@220 : Injection 1

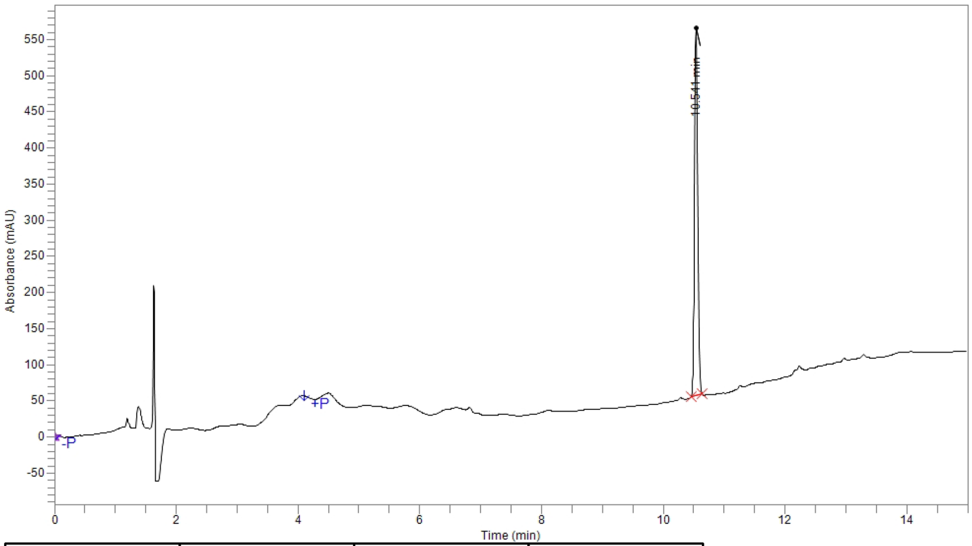

Time	Height	Area	Area %
10.541	509,904.8	1,847,231.4	100.00
**Total**		1,847,231.4	100.00

Compound 5 @254 nm

OX-AW83@254 6/15/2021

Acquisition Method	Purity short run @254 nm
Acquisition Date/Time	6/7/2021 6:03 pm
Injection Volume	10
Sample Name	OX-AW83@254
Sample Description	
Batch Description	

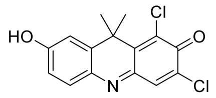

5

OX-AW83@254 : Injection 1

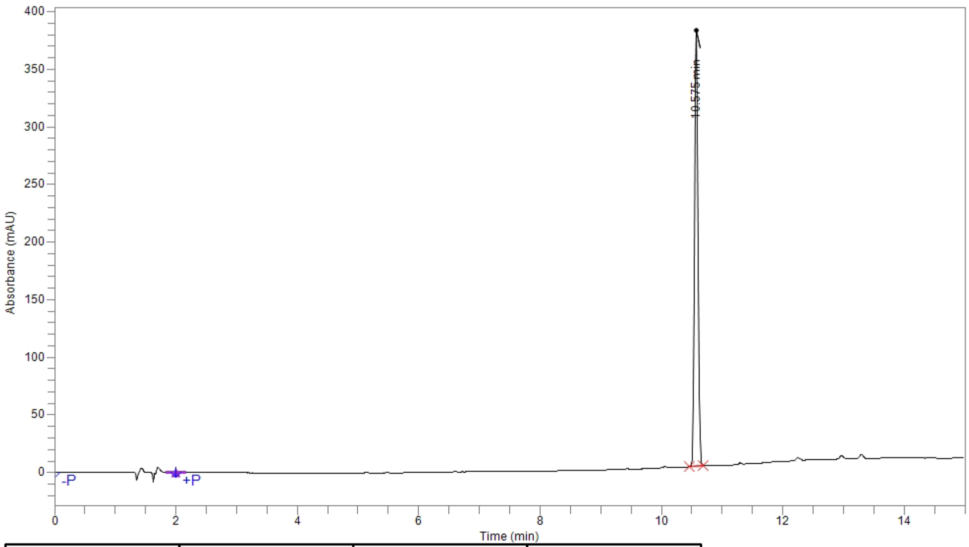

Time	Height	Area	Area %
10.575	379,025.3	1,373,353.7	100.00
**Total**		1,373,353.7	100.00

Compound 7 @220 nm

Acquisition Method	Purity short run @220 nm
Acquisition Date/Time	6/7/2021 5:27 pm
Injection Volume	10
Sample Name	OX-AW80@220
Sample Description	
Batch Description	

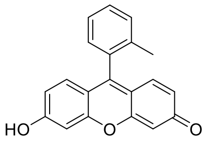

7

OX-AW80@220 : Injection 1

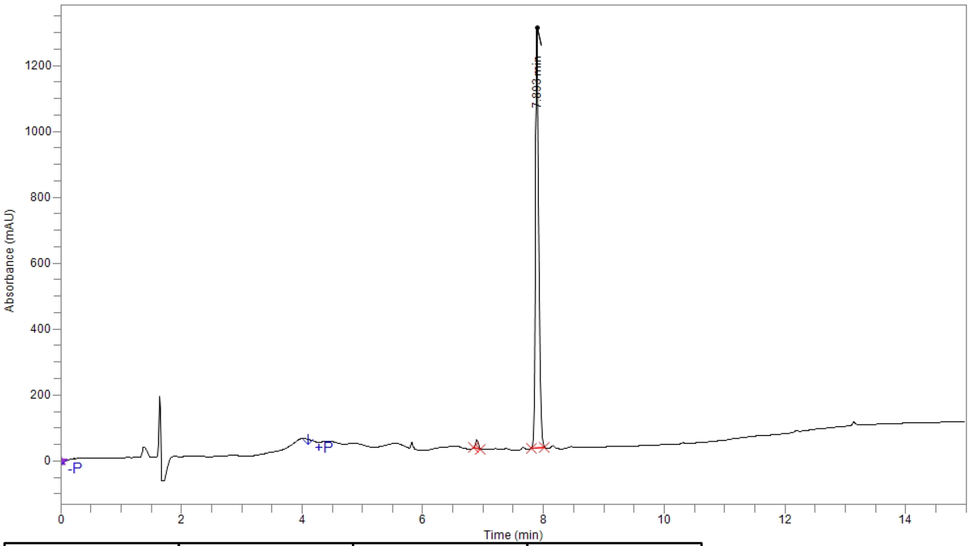

Time	Height	Area	Area %
6.896	26,300.2	79,552.5	1.49
7.893	1,277,280.0	5,247,633.0	98.51
**Total**		5,327,185.5	100.00

Compound 7 @254 nm

Acquisition Method	Purity short run @254 nm
Acquisition Date/Time	6/7/2021 5:07 pm
Injection Volume	10
Sample Name	OX-AW80@254
Sample Description	
Batch Description	

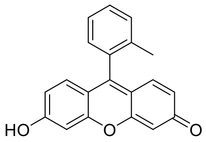

7

OX-AW80@254 : Injection 1

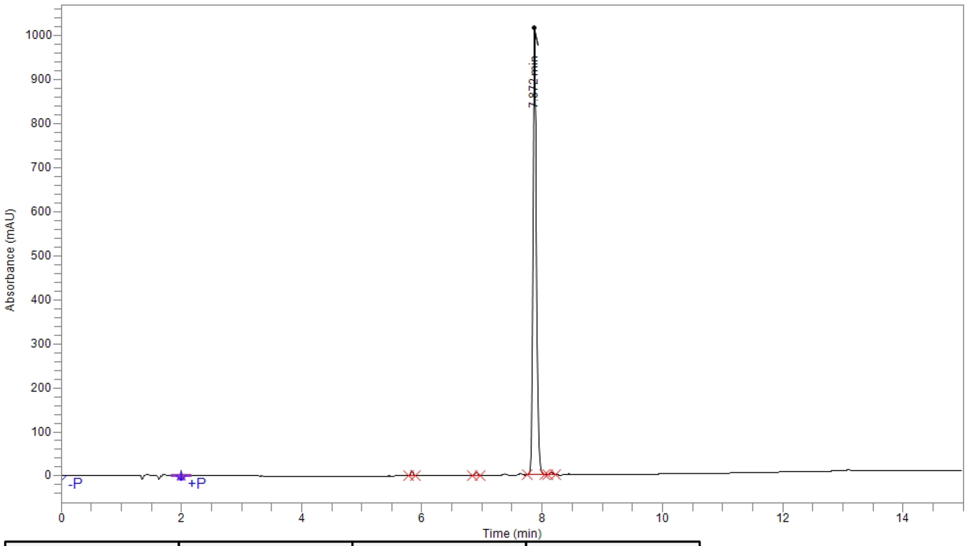

Time	Height	Area	Area %
5.835	11,311.5	33,287.5	0.75
6.907	7,261.2	23,725.9	0.53
7.872	1,017,438.1	4,374,422.8	98.30
8.155	5,137.0	18,577.6	0.42
**Total**		4,450,013.7	100.00
